# Comparative Secretome Analysis Reveals Perturbation of Host Secretion Pathways by a Hypovirus

**DOI:** 10.1038/srep34308

**Published:** 2016-10-04

**Authors:** Jinzi Wang, Liming Shi, Xipu He, Lidan Lu, Xiaoping Li, Baoshan Chen

**Affiliations:** 1 State Key Laboratory for Conservation and Utilization of Subtropical Agro-bioresources and Key Laboratory for Microbial and Plant Genetic Engineering, Ministry of Education, College of Life Science and Technology, Guangxi University, Nanning 530004, China

## Abstract

To understand the impact of a hypovirus infection on the secretome of the chestnut blight fungus, *Cryphonectria parasitica*, a phytopathogenic filamentous fungus, two-dimensional electrophoresis (2-DE) and isobaric tag for relative and absolute quantitation (iTRAQ) technology were employed to identify and quantify the secreted proteins. A total of 403 unique proteins were identified from the secretome of the wild type virus-free strain EP155. Of these proteins, 329 were predicted to be involved in known secretory pathways and they are primarily composed of metabolic enzymes, biological regulators, responders to stimulus and components involved in plant-pathogen interactions. When infected with the hypovirus CHV1-EP713, 99 proteins were found to be differentially expressed as compared to the wild type strain EP155. These proteins were mainly related to plant cell wall degradation, response to host defense, fungal virulence and intracellular structure. The effects of CHV1 on secreted proteins may reveal a relationship between physiological pathways and hypovirulence.

The chestnut blight fungus, *Cryphonectria parasitica* is a well-known forest pathogenic fungus which destroyed billions of American chestnut. Physiological and pathogenetic aspects of this fungus have been investigated deeply^1^. Transcriptional research based on EST library and cDNA microarrays revealed a wide range of pathogenicity-related genes^2^,^3^. A useful model system for the study of mycovirus-host interactions and fungal pathogenesis has been established based on the hypovirus, associated with *C. parasitica*^1^. Recently, the proteomic analysis was also carried out and some useful information about perturbation of host proteins and splicing forms of viral proteins was found^4^. However, the proteomic study of this model system was just in start-up phase.

Secreted proteins have been implicated for pathogenesis in bacteria and fungi^5^,^6^, and large scale of secretome research has been performed in certain organisms^7^,^8^. A study on the secretome could aid in elucidating the interactions between organisms and their environment^9^,^10^. In the kingdom of fungi, the secretome of yeast got relatively comprehensive study and 81 unique proteins were identified by physical and computational analysis in *Kluyveromyces lactis*^11^. Due to the complicated and difficult preparation of extracellular samples, the secretome research on phytopathogenic fungi was still limited and only a small part of secreted proteins have been effectively identified^6^,^12^,^13^. A part of secreted proteins have been confirmed as filamentous plant pathogen effectors and essential for pathogen invasion through gene function research^14^. Sufficient experimental data and information from proteomic analysis will be helpful to better understand the secretory pathway for filamentous fungi^15^.

One of the most studied and well-known secreted protein of *C. parasitica* is a fungal hydrophobin, cryparin. This protein is most abundant and essential for stromal pustule eruption^16^,^17^. Cryparin contains a signal peptide which directs it to the vesicle-mediated secretory pathway and post-translationally processes by Kex2 endoprotease^18^. In a further study, cryparin-GFP fusion protein was used as a marker to monitor secretion in wild-type and viral infected strains^19^. Meanwhile, the sub-proteomic study of fungal secretory vesicle was carried out^4^. These experimental results suggested that the virus perturbed trans-Golgi network mediated secretory pathway which was important in fungal development and virulence.

In this study, we used modified sevag method to prepare high quality secreted proteins from *C. parasitica*, that were suitable for proteomic analysis^20^. Two-dimensional electrophoresis (2-DE) and isobaric tag for relative and absolute quantitation (iTRAQ) technology were selected to analyze secreted proteins. The identified secreted proteins were classified and searched via BLAST against the Fungal Secretome Database (FSD)^21^. The investigation of the regulated fungal secretome upon hypovirus infection was also carried out. The current study provides important experimental information on the secretome for this pathogenic filamentous fungus and gives direct experimental evidence to interpret the relationship between hypovirulence and secreted proteins.

## Results

### Time course of protein secretion, and 2-DE and Mass spectrometry analyses

**Figure 1:**
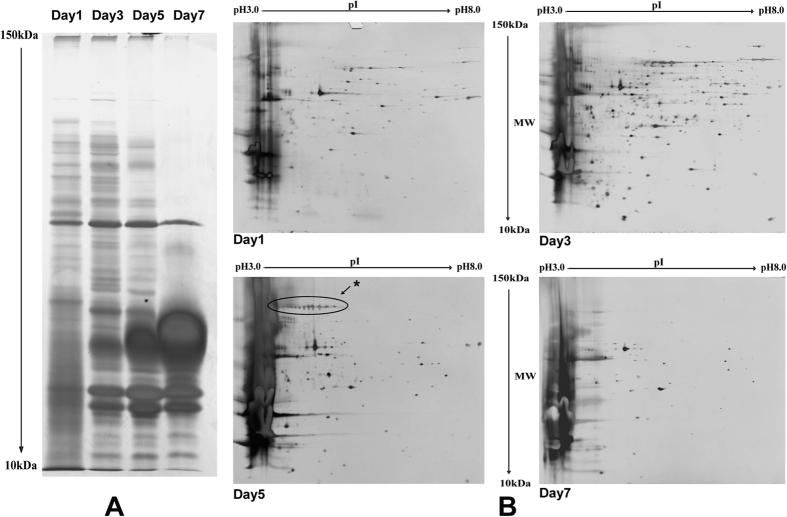
Time course of protein secretion. (**A**) SDS-PAGE analysis of secreted proteins from different culture time. Ten μg of secreted protein was loaded in each lane of SDS-PAGE gel. (**B**) 2-DE analysis of secreted proteins. Equivalent amounts (200 μg) of secreted protein were loaded on 2-DE system. The culture time of secreted protein samples were marked beside the corresponding lane and 2-DE gel. The protein spots marked (*) in 2-DE gel of day 5 were identified as laccase A.

Protein samples prepared using the modified sevag method^20^ yielded a high quality PAGE and 2-DE separation (Fig. 1). As seen on 2-DE analysis of wild type EP155, maximum number of proteins could be recovered from the medium at day 3. As the culture time progressed to day 5, the number of proteins dropped and a few proteins accumulated to a much higher abundance at day 7. At this stage, most of proteins with higher molecular weight (MW) disappeared while proteins with lower MW accumulated to a higher level, mainly because of the over expression and accumulation of several high abundant secreted proteins with lower MW (Fig. 1). These characteristics of protein secretion time course were confirmed by 2-DE analysis: 130 ± 10 proteins was found from day 1 sample, 382 ± 20 from day 3 sample, 145 ± 10 from day 5 sample, and 82 ± 10 from day 7 sample (Fig. 1B). Since the largest number of proteins was recovered on day 3, this time point was set to be the prime time to collect secreted proteins in this study. The protein spots appeared on 2-DE gels were extracted and trypsin-digested for MS identification. A total of 101 unique proteins were successfully identified (Fig. S-1, Table S-1). The highest abundant secreted protein (No. 107, 22 kDa glycoprotein) could be erased from secretome by knockout of the coding gene (Fig. S-2).

### Identification and quantification of secreted proteins

iTRAQ MS/MS technology was used to identify secreted proteins. A total of 403 secreted proteins labeled with iTRAQ kit were successfully identified in all three independent experiments. Among these, ninety-nine proteins were classified as differentially expressed with change of ±1.5-fold or more in abundance upon hypovirus infection (Tables 1 and 2, Table S-2 for detailed information, Table S-8 with single peptide information). Proteins that did not show a significant change upon hypovirus infection were listed in Table S-3 and the identified proteins which did not show up in all of the three independent experiments were listed in Table S-4. As the negative control, fresh EP complete medium was also concentrated and analyzed by mass spectrometry to exclude possible protein contaminants. Three proteins were identified from EP medium under the same experimental conditions and only one of them appeared in iTRAQ identification with no significant change between virus-free strain EP155 and virus-infected EP713 (Table S-5).

### Classification and characterization of the secreted proteins

**Figure 2:**
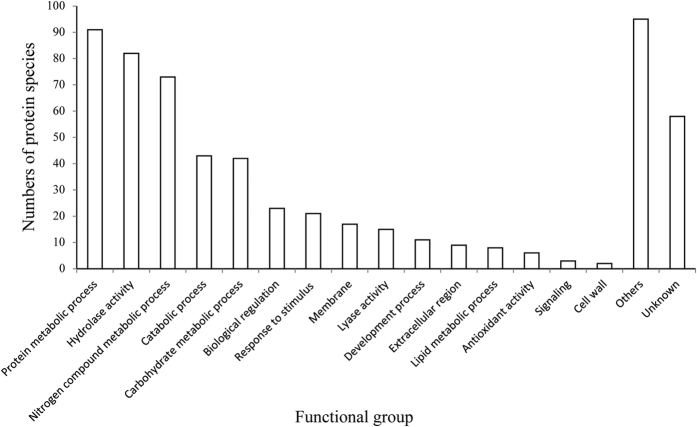
Distribution of secreted proteins according to functions. Note: some proteins may have been considered more than one time and included in more than one pathway.

Secreted proteins identified were subject to GO annotations by QuickGO^22^ to form the original plot. As could be seen in Fig. 2, a series of biological metabolic activities occurred in the extracellular medium to aid the fungus in nutrient utilization and survival. Fifty-eight of the 403 proteins failed to get GO annotations.

Fungal Secretome Database (FSD) is an integrated platform for annotation of fungal secretomes with multiple prediction tools^21^. A BLAST search against FSD revealed that 326 proteins, approximately 80% of the proteins identified, had matched sequences at expectation value 1e-^50^. Forty-eight percent of the proteins belongs to Class SP^3^ type, 22% to Class SP, 6% to Class SL, 6% to Class NS, and 18% has no match in the FSD (Table S-6). The non-matched proteins include mainly structural proteins, intracellular enzymes, and uncharacterized proteins.

### Confirmation of the expression level of secreted proteins

**Figure 3:**
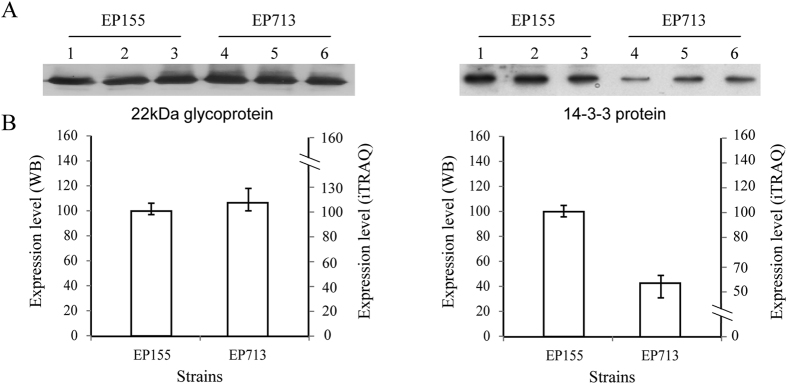
Western blot quantification of the 22 kDa glycoprotein and 14-3-3 protein. The protein was detected with specific polyclonal antibodies. An amount of 50 μg of protein per sample from three independent extractionswas loaded and separated on 12% PAGE. After transfer to a PVDF membrane, the blot was detected by 22 kDa glycoprotein-specific antibody or 14-3-3 protein-specific antibody. Lanes 1–3 represent samples from independent preparations (**A**). Semi-quantification of the blots indicated that the 22 kDa glycoprotein was expressed basically at the same level in the fungal strains, EP155 and EP713, while 14-3-3 protein was significantly down-regulated in EP713 (−2.5 fold) as compared with that of EP155, consisting well with the quantification results of iTRAQ analysis (**B**).

To confirm the accuracy of secreted protein expression level detected by iTRAQ, antibodies against non-differentially expressed protein (the 22 kDa glycoprotein) and differentially expressed protein (the 14-3-3 protein) between virus-free strain EP155 and virus-infected strain EP713, were quantified by Western blotting (Fig. 3). The results showed that 22 kDa glycoprotein was at similar level in strains EP155 and EP713, whereas 14-3-3 protein was at a lower level in EP713 compared with EP155, demonstrating the highly accordance between iTRAQ and Western blot analysis.

### Comparison of protein level in and out of the cells

**Figure 4:**
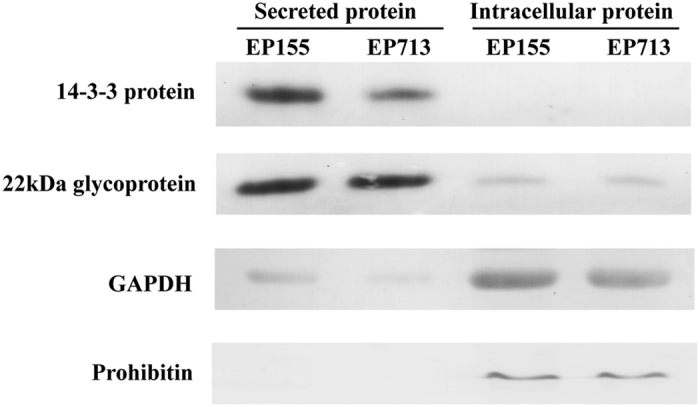
Western blot analysis of the 14-3-3 protein, 22 kDa glycoprotein, GAPDH and prohibitin. Equivalent amounts (20 μg) of proteins from secreted proteins and intracellular proteins were loaded. The changing tendencies of these proteins in different secreted samples were consistent with the iTRAQ analysis. Furthermore, the results of Western blotting showed that fungal cell secreted proteins into the environment in varying degrees. 14-3-3 protein was just observed in secreted samples. Meanwhile prohibitin was only appeared inside cells. The accumulations of 22 kDa glycoprotein and GAPDH showed great difference between intra- and extra- cellular spaces.

Since proteins detected in medium could be the result of active secretion, or passive release due to cell lysis, we performed Western blot analysis of secreted and intracellular proteins. As shown in Fig. 4, 14–3–3 protein and 22 kDa glycoprotein were mostly in the medium, whereas GAPDH and prohibitin which were considered to play their roles intracellularly were mostly in the cells, showing an active secretory mechanism, rather than a random release by cell lysis due to cell death.

### Correlation of mRNA level and protein level

mRNA extracted from the fungal mycelia from one of the three sample replicates for secreted protein preparation were subject to digital quantification by RNA-seq. A comparison of the mRNA level (Supplemental Table 7) and the secreted protein level of EP155 and EP713 revealed that there was a complex correlation in general between the mRNA level and protein level for individual genes (Tables 1 and 2), suggesting that transcription regulation, post transcription regulation, and secretion regulation may all influence the outcome of a secreted protein.

## Discussion

We used 2-DE and iTRAQ technology to analyze the secretome of *C. parasitica* and identified more proteins, as compared with previous reports on the fungal secretome^6^,^12^,^13^. The 2-DE system was convenient and straight forward to observe protein expression level than other proteomic techniques. But with complex samples such as fungal secreted proteins in this study, gel resolution and background were hard to optimize. This situation could lead to low protein spots recognition and low matching rate and further interfere with MS analysis. A better resolution of secretome could be achieved in 2-DE by knocking out the coding gene of the highest abundant secreted protein (Fig. S-2). A comparison of the 2-DE of the wild type and the 22 kDa glycoprotein knockout mutant reveals that some new protein spots appeared while some disappeared, for example, the cell wall related proteins pectin lyase A (No. 42 and 43), PhiA (No. 129) and glucanase (No. 130) were significantly down-expressed, which would seriously impact the normal cell wall construction. Meanwhile, the Rho GDP-dissociation inhibitor (No. 134 and 153) was up-expressed which may result in the activation of the superoxide-forming NADPH oxidase^23^. This phenomenon suggests that 22 kDa glycoprotein as a secreted protein regulates other secreted proteins. Further study on the 22 kDa glycoprotein may provide new insights into the regulation network of secretome in fungi.

We observed that some protein spots, such as No. 137 identified to be 3-phytase A precursor, appeared to be with much lower molecular weight than predicted (11 kDa via 58 kDa). We assume that these proteins may have been processed by a protease either before or after the secretion. Giving the harsh environment in the culture medium, protein breakdown seems to be unavoidable, but the speed of degradation may vary from protein to protein, as shown in the secretion time course (Fig. 1). In this regard, 2-DE coupled with mass spectrometry is a good method to detect and identify the protein isoforms.

To increase the throughput of protein detection and quantitation, iTRAQ technology was employed to analyze the secreted proteins. The number of proteins identified was almost 4 times as many as those identified by the 2-DE (101 proteins, Fig. S-1 and Table S-2) and more than 95% of 2-DE derived proteins were covered by iTRAQ identification (Table S-1). To ensure the quality of secreted protein samples and to exclude possible contaminants, Amicon 10-kDa centrifugal filters were used to remove intracellularly degraded peptides before protein digestion and iTRAQ labeling. This measure also effectively discriminated the possible contamination by the degraded peptides derived from the culture medium.

A large proportion of the secreted proteins were identified to be extracellular enzymes that take part in nutrients utilization and possess hydrolase and lyase activities. Others are involved in interaction between the fungus and the external environment including response to stimulus, antioxidation, cell development and signal transduction (Fig. 2). There were 58 proteins with unknown functions and 95 proteins with no apparent relationship with extracellular functions. By Western blotting analysis of the intracellular and extracellular location specificity of four proteins, we further demonstrated the secretion of proteins in *C. parasitica* was an active but not a passive process (Fig. 4), i.e., proteins in the medium were unlikely released due to the cell death or rupture.

Computational analysis of the experimental data revealed that an integrated platform was necessary for fungal secretome prediction. FSD uses several methods to predict the secretome independently and provides a complete and detailed report of the sequence BLAST information^21^. It was predicted by using the FSD platform that the putative secretome of *C. parasitica* includes 2,084 proteins from 11,184 ORFs. The experimental secretome, containing 403 proteins, is much smaller than the putative secretome. BLAST searching identified 329 proteins as putative secretome proteins from *C. parasitica* (Table S-6). Certainly one can not obtain all secretome information from one set of experiment, as the proteins may secrete at different times and different conditions.

Proteins playing important roles in the infection process, such as cell wall degradation, anti-host defense, virulence and intracellular structural proteins, were identified in the secretome of *C. parasitica* (Tables 1 and 2). Triosephosphate isomerase (TPI), an enzyme that catalyzes dihydroxy acetone phosphate to glyceraldehyde-3-phosphate was among the list. TPI has been shown to perform an adhesion function in the human pathogenic fungus *Paracoccidioides brasiliensis*^23^. We speculate that TPI may play a role in plant fungal pathogens during invasion of host cells. Pectinase, cell wall glucanase, xylanase, chitinase and celluase were all described as cell wall-degrading enzymes^24^. Pectinase can degrade pectic compounds from the plant cell wall to aid mycelium in penetrating and destroying the host cell walls^25^. Glucans and glucanase exist both in plant and fungal cell walls^26^ and their interactions may illustrate the plant-pathogen interactions, including elicitation of plant defenses^27^. Deletion of these enzymes in *Botrytis cinerea* reduced its pathogenicity^28^.

More interestingly, a large part of these infection-related proteins were regulated by hypovirus (Tables 1 and 2). In *C. parasitica*, cutinase which was necessary to degrade plant cuticles and help pathogenic fungus to penetrate into the host cell was confirmed to be suppressed by hypovirus^29^. The activity of extracellular cellulase was detected when cellulose was taken as sole carbon source. Northern blot analysis revealed that hypovirus infection reduced transcript accumulation and enzyme activity of extracellular cellulase^30^. Just like we described above, a large list of cell wall-degrading enzymes appeared to be suppressed in hypovirus-infected strain EP713. Thus, down regulation of a set of cell wall-degrading enzymes is a mechanism of hypovirus perturbation of fungal pathogenicity. This observation may partly explain the failure of previous experiment by knocking out a single gene encoding cell wall-degrading enzyme that did not show a hypovirulent phenotype^31^.

Plants have defense systems to protect themselves when attacked by pathogenic fungi. Pathogens, in turn, respond by unarming the host defense ability to aid its infection. In *Blumeria graminis*, peroxidase/catalase was shown to secrete outside the cell^32^. It reduces the effect of reactive oxygen species (ROS) on fungi, the production of which is the most common defense response of plants^33^,^34^. The same situation appeared in *Fusarium graminearum* with Cu-Zn superoxide dismutase (SOD), which was also detected outside the cell^35^,^36^. In this study, we showed that SOD secreted by the *C. parasitica* was down-regulated by hypovirus, providing a line of evidence that hypovirulence of EP713 may in part result from its lowered ability to encounter ROS stress imposed by the host plant.

The 14-3-3 proteins are a class of highly conserved proteins, which can be found in all eukaryotes^37^. They are able to bind numerous proteins and are involved in many biological processes. In *Candida albicans*, one type of 14-3-3 protein can mediate pathways associated with virulence^38^. Observation of this protein in secretome of *C. parasitica* and suppressed expression level in EP713 suggests that pathogenic fungi adapt to the environment via their own protein-protein interactions^39^ and this process was disrupted by hypovirus infection.

Cyclophilin has been implicated in pathogenesis of the rice blast fungus *Magnaporthe oryzae*, by regulation of appressorium turgor generation, lipid biosynthesis, and the development of asexual spores^40^. A functional homologue of cyclophilin-encoding gene (*cyp1*) is also present in *C. parasitica*, which is initially annotated by the analysis of expressed sequence tags^41^. This gene has been recently shown to be a virulence factor and to have a positive correlation with the expression of key components of the heterotrimeric G-protein signaling pathway^42^. Down-regulation of CYP1 both in intra- and extracellular by hypovirus thus can explain in part the mechanism underlying the hypovirulence of *C. parasitica* upon being infected by a hypovirus.

One of the most important housekeeping enzymes, glyceraldehyde-3-phosphate dehydrogenase (GAPDH), was observed to be differentially secreted in our proteomic results. In the study of pathogenic microorganisms, GAPDH was found to appear on cell surface of *Streptococcus* spp., *Candida albicans* and *Escherichia coli* playing various roles including transferrin binding, surface antigen and signal transduction between pathogens and host cells^43^,^44^,^45^,^46^. The role of GAPDH served as a potential virulence factor has been discussed^47^.

Laccase is a poly-phenol oxidase and related to fungal virulence^48^, the pigmentation of fungal spores^49^, and lignin degradation^50^. In *C. parasitica,* laccase A is extracellularly secreted and suppressed by the presence of hypovirus^51^. This protein was observed in sample of day 5, migrating from about pI 3.5 to pI 5.0 in 2-DE. The pI of the nascent laccase A is 5.4 and this enzyme was reported to function best at pH 2.5^48^,^51^. Thus, we speculate that phosphorylation modification causes this migration pattern (Fig. 1). In addition to laccase A, laccase 3 was identified by iTRAQ in sample of day 3. It seems that laccase 3 and laccase A are secreted at different time, forming a time scenario of secretion of laccase enzymes in *C. parasitica*. Both laccase 3 and laccase A were down-regulated by the hypovirus infection.

Cryparin was not identified in our current study. Cryparin is known to be secreted at high levels^16^. Cryparin is a fungal hydrophobin and could be secreted into the culture medium, but it bounds to the cell wall rapidly and entirely, resulting in little amount in the culture medium^16^,^17^.

A number of intracellular proteins, such as ribosomal proteins and nascent polypeptide-associated complex (NAC), were found up-regulated in the medium with hypovirus-infected strain EP713 (Table 1). How these proteins enter into the medium remains unknown. Observation under light microscope revealed intact mycelia and gel electrophoresis of the extract of the culture medium with fungal mycelia showed no sign of degraded DNA for both EP155 and EP713 (our unpublished data). A second reason not in favor of the assumption of cell death and rupture is that intracellular proteins released were not in proportion to the proteins within the cell. Thus a significant cell death or rupture in EP713 sample of day 3 could be ruled out. It is speculated that release of intracellular proteins in EP713 could be through an unspecified process. *C. parasitica* is composed of a rigid cell wall and broken mycelia of *C. parasitica* in liquid medium is hard to distinguish.

The knowledge of secretory pathway in filamentous fungi was still limited. It was generally divided into classical and non-classical pathway^15^. Fungal cells utilized endoplasmic reticulum (ER) and Golgi compartment to process secretory protein through vesicle-mediate transport system in classical pathway^15^. In previous study, the vesicle-mediate transport system of *C. parasitica* was found to be disturbed by hypovirus infection and the transport efficiency of cargo proteins was reduced^4^. Inspection of mRNA abundance determined by RNA-seq (Supplemental Table 7) revealed a different pattern as compared with protein pattern in the secretome (Tables 1 and 2), suggesting that both mRNA regulation and secretory pathway regulation contribute to protein secretome. Combined, we propose that hypovirus targets both the secretory pathway and vesicle-mediate transport system to regulate the protein secretion in *C. parasitica*.

**Figure 5:**
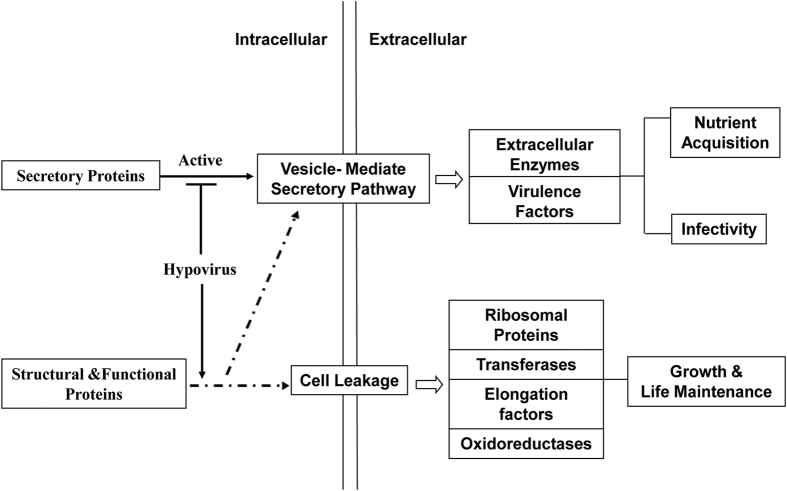
Potential relationship reflected by fungal secretome between hypovirulence and virus infection. The fungal secretome consisted by intracellular components which secreted followed cell leakage, and extracellular components regulated by secretory pathway. After virus infection, intracellular components and secretory pathway were both regulated. This situation was shown by secretome changing tendency and further indicated that the aspects of nutrition acquisition, infective ability, energy metabolism and cell aging and death of this pathogenic fungus were regulated by virus infection which synthetically led to the phenomenon of hypovirulence.

With the identification of the secretome and unveiling the discrepancy between the wild type strain EP155 and hypovirus-infected strain EP713, we suppose that hypovirus perturbs the secretory pathways is one of the major mechanisms responsible for hypovirulence in *C. parasitica*. Viral infection results in reduction of a group of extracellular enzymes vital for the fungus to acquire nutrients from the environment and pathogenicity-related factors to encounter the host defense, and leakage of intracellular functional proteins that would impact the fitness of the fungus (Fig. 5). Finally, the availability of a list of secreted proteins and hypovirus as a tool to manipulate the secretome of *C. parasitica* provides a key to probe the protein secretion mechanisms, including the classical pathway and the non-classical pathway in a pathogenic fungus.

## Methods

### Fungal strains and culture conditions

The fungal strains used in this work were the virus-free strain EP155 (ATCC 38755) and virus-infected strain EP713 (ATCC 52571). The fungal strains were cultured on solid potato dextrose agar (PDA) medium and EP complete liquid medium. The culture condition was described in the previous study^20^.

### Extraction and 2-DE analysis of the fungal proteins

The intracellular fungal proteins were extracted from cultured mycelia in EP liquid medium by using TCA-acetone method. A half gram of fungal mycelia was ground into powder in liquid nitrogen and re-suspended in 1 ml of pre-cold acetone (−20 °C) containing 10% TCA and 0.07% β-mercaptoethanol. After incubation at −20 °C for 30 min, the protein was pelleted at 18,000 g at 4 °C for 20 min. The protein pellet was washed with ice-cold acetone, air-dried, and then re-suspended in 1 ml of lysis buffer (7.5 M urea, 2.5 M thiourea, 12.5% glycerol, 50 mM Tris, 2.5% n-Octylglycoside, 6.25 mM TCEP, and 2% protease inhibitor). After being ultrasonicated at 200 W for 1 min with 12 s/interval 15 s, the protein sample was centrifuged at 18,000 g at room temperature for 20 min. The supernatant was stored at −20 °C and prepared for Western blotting.

The modified sevag method was chosen for extraction and purification of the secreted proteins. A half volume of chloroform/butanol (4:1) was added to the mycelia-free culture medium and mixed thoroughly. The protein-containing interface phase was transferred and centrifuged at 10000 g for 5 min. After removing the supernatant, the pellet was washed 3 times with washing buffer (0.3 M guanidine hydrochloride in 95% ethanol), and one time with anhydrous ethanol. The pellet was dissolved in lysis buffer (7 M urea, 2 M thiourea, 4% CHAPS, 1% DTT, 0.5% cocktail of protease inhibitors) and centrifuged at 18000 g at 4 °C for 20 min. The proteins in the supernatant were precipitated by using TCA-acetone method as described above. The dried protein pellet was solubilized in 100 μl of lysis buffer.

The analysis of 2-DE and in-gel mass spectrometry were carried out as previously described^20^. Each of 200 μg proteins was rehydrated in the rehydration buffer and applied to a non-linear pH 3–10 IEF strip. Isoelectric focusing was carried out on a IPGphor (GE Healthcare, USA) using the following parameters: 30 V, 6 h; 60 V, 6 h; 500 V, 1 h; 1000 V, 1 h; 1000–6000 V, 4 h; and 6000 V, 120000 Vh. After reduction and alkylation procedures, the strips were mounted onto 12.5% polyacrylamide gels for second dimension electrophoresis. Three independent experiments of biological repeats were carried out to check the reproducibility of the protein samples from each time.

### In-gel tryptic digestion and TOF-TOF-MS identification

In-gel digestion of protein was done according to the established protocol^52^. The peptides solution with CHCA matrix solution were analyzed on 4800 plus MALDI-TOF/TOF mass spectrometer (Applied Biosystems, USA) in the m/z range 800–3500. The combined PMF search was carried out using GPS Explorer™ software with the MASCOT search engine against *C. parasitica* database v1.0 (39 genome scaffolds totaling 43.9 MB, 11,184 gene models) from JGI website (http://genomeportal.jgi-psf.org/Crypa1/Crypa1.download.ftp.html) on a local server.

### iTRAQ labeling and strong cation exchange fractionation

Amicon 10 kDa centrifugal filters (Millipore, USA) were used for protein purification and concentration before labeling. An amount of 100 μg pre-treated protein samples were reduced, alkylated, digested and labeled with iTRAQ kit (Applied Biosystem) according to the manufacturer’s protocol. iTRAQ reagent 117 and 116 were used to label protein samples of EP155 and EP713, respectively. Three independent biological samples (fungal culture batches) were used to ensure the reproducibility of the results.

Labeled peptides were subjected to strong cation exchange (SCX) fractionation and separated by Agilent 1100 HPLC (Agilent Technologies, USA) using a Polysulfoethyl 4.6 × 100 mm column (5 μ, 200 Å) (PolyLC Inc, USA). Fractions were collected automatically into a microwell plate with AFC fraction collector using SCX buffer A (10 mM KH_2_PO_4_, 25% ACN) and B (500 mM KCl, 10 mM KH_2_PO_4_, 25% ACN). The following method of gradient elution with buffer B was used: 0–10% B for 7 min, 10–20% B for 10 min, 20–45% B for 5 min, and 45–100% B for 5 min. Collected fractions were dried by vacuum centrifugation and stored at −20 °C.

### RPLC and MS/MS identification

Reversed-phase high performance liquid chromatography (RPLC) analysis of SCX fractions was carried out on Zorbax 300SB-C18 peptide traps (Agilent Technologies, USA) using RP-C18 0.15 × 150 mm column (Column Technology Inc.) with buffer A (0.1% methanoic acid) and B (0.1% methanoic acid, 84% ACN) by gradient elution: 0–4% B for 1 min, 4–50% B for 100 min, 50–100% B for 12 min, and 100% B for 6 min.

After desalination and separation by RPLC, the peptides were analyzed on LTQ Orbitrap Velos (Thermo Scientific, USA) in a data-dependent mode with the MS scan range of m/z 350–1800. The survey scans were acquired through the Orbitrap analyzer at a normal mass resolution of 60,000 at 400 m/z. Precursor ion isolation window width was set to 2 amu. Dynamic exclusion settings were: Repeat count 1, Exclusion list size 500, Exclusion duration 80 s, Exclusion mass width relative to precursor ±10 ppm. Eight of most intense precursor ions were selected for MS/MS in the mode of collision induced dissociation (CID) with 35% normalized collision energy, activation Q 0.7, and activation time 100 ms. Charge state screening was on 1+ and unassigned rejected. MS/MS spectrum was acquired in the Ion Trap analyzer at normal speed. Proteome Discoverer 1.3 (Thermo Fisher Scientific) software was used to search the mass spectrometric data with SEQUEST search engine against *C. parasitica* database v1.0 from JGI website (http://genomeportal.jgi-psf.org/Crypa1/Crypa1.download.ftp.html). Precursor ion mass tolerance was set to 10 ppm and fragment mass tolerance was set to 0.8 Da. Two missed cleavages were allowed using trypsin as endoprotease. iTRAQ modification of lysine residues and peptide N termini was set as fixed modifications and variable modifications respectively. The peptides from known contaminations such as keratin were excluded in search parameters. A decoy database search for determining false discovery rate (FDR) was set for maximum 1%. A protein that appeared in all three independent experiments was considered valid.

### Antibody preparation and Western blot analysis

The peptide CQQSYTGPTAFDLSD of the 22 kDa glycoprotein which was identified as a highly abundant secreted protein in 2-DE gel^20^, was used to generate polyclonal antibody in rabbit (GenScript USA Inc., Chinese branch, Nanjing). The antibodies against 14-3-3 protein, GAPDH and prohibitin were purchased from Bioss Inc (China). Antibodies at 1:1000 dilutions were used for Western blotting. Secreted protein samples were separated in 12% SDS-PAGE and transfered to PVDF membranes (Millipore, USA) in Hoefer^TM^ TE 77 semi-dry transfer unit (Hoefer, USA). Pierce Western blotting substrate (Thermo Scientific, USA) was used to detect immunoblotting, following instruction of the manufacturer.

### Computational analysis of the secreted proteins

The identified proteins by iTRAQ technology were classified according to GO using the QuickGo online tools^22^. BLAST search against secretomes of other species was performed on FSD platform^21^. For comparative analysis of secretomes, the significance level was set at 95% (p < 0.05) for each individual protein. Peptides which may be contained in different proteins were filtered and excluded. A threshold of 1.5-fold change was set to define a regulated expression. An average from three independent experiments for each protein expression level was adopted.

### Fungal RNA extraction and sequencing

Total RNA was extracted according to the established method in previous report^53^. The same sample collected on the same day and time was used for the mRNA extractions. mRNA selection, library preparation and sequencing was performed on an Illumina GAIIx sequencer according to manufacturer specifications. We sequenced two 81-cycle paired-end lanes and analyzed transcriptomic data using TopHat and Cufflinks protocol^54^.

## Additional Information

**How to cite this article**: Wang, J. *et al*. Comparative Secretome Analysis Reveals Perturbation of Host Secretion Pathways by a Hypovirus. *Sci. Rep.* **6**, 34308; doi: 10.1038/srep34308 (2016).

## Supplementary Material

Supplementary Information

## Figures and Tables

**Table 1:** List of up-regulated secreted proteins upon hypovirus infection by HPLC-ESI-OrbiTrap MSMS.

No.	Protein ID^a^	Protein Name	Average change (EP713/EP155)^b^	Standard error	Unique PepCount^c^	Cover Percent^d^	MW^e^	pI^f^
		**Ribosomal proteins**						
491	54955	Small nuclear ribonucleoprotein F	3.3 (−)	0.12	1	9.89%	10327.75	5.3
131	75598	60S ribosomal protein L12	2.2 (−)	0.09	5	34.34%	17645.42	9.41
109	79283	40S ribosomal protein S5	1.8 (↑)	0.07	6	28.84%	23791.27	9.28
154	100785	40S ribosomal protein S14 (CRP2)	1.8 (↑)	0.05	5	39.74%	16045.36	10.68
398	90732	40S ribosomal protein S19 (S16)	1.8 (↑)	0.02	2	13.16%	16681.79	9.18
128	101628	40S ribosomal protein S0 (Ribosome-associated protein 1)	1.7 (↑)	0.05	5	19.30%	31041.9	4.76
173	103417	60S ribosomal protein L30	1.7 (↓)	0.13	4	29.09%	11647.55	9.77
254	103140	60S acidic ribosomal protein P0	1.6 (↑)	0.04	3	9.55%	33639.19	4.62
350	54024	40S ribosomal protein S28	1.6 (↑)	0.1	2	31.34%	7608.81	10.37
		**Oxidoreductase activity**						
61	93945	Indoleamine 2,3-dioxygenase family protein	2.8 (↓)	0.08	9	16.60%	55419.63	5.43
482	55348	NADPH-dependent D-xylose reductase II, III (XR)	1.8 (−)	0.1	1	3.07%	36430.6	6.15
41	99052	Protein disulfide-isomerase precursor (PDI)	1.7 (−)	0.05	11	26.88%	55317.38	4.69
171	91352	Quinone oxidoreductase	1.7 (−)	0.03	4	19.51%	21569.41	5.46
22	99412	Monodehydroascorbate reductase	1.6 (↓)	0.06	13	29.49%	57358.2	6.38
46	87808	Formate dehydrogenase (NAD-dependent formate dehydrogenase) (FDH)	1.6 (−)	0.02	10	21.56%	48658.25	8.65
118	80754	Superoxide dismutase-1(SOD-1)[Cu-Zn]	1.6 (↓)	0.01	5	37.42%	15945.45	5.85
191	87102	Uricase	1.5 (−)	0.07	4	15.49%	33212.42	6.01
226	107257	NADH-cytochrome b5 reductase	1.5 (↓)	0.09	4	13.49%	37374.71	9
		**Transferase activity**						
32	42588	UTP-glucose-1-phosphate uridylyltransferase	1.6 (↓)	0.04	12	20.70%	57401.52	6.19
364	58903	4-aminobutyrate aminotransferase	1.6 (↑)	0.04	2	3.64%	51778.91	5.94
373	85870	Glucosamine-fructose-6-phosphate aminotransferase	1.6 (↓)	0.14	2	3.11%	77900.66	6.19
		**Ion binding**						
161	82952	Pyruvate carboxylase (Pyruvic carboxylase) (PCB)	1.5 (↓)	0.09	5	4.14%	130792.42	6.15
238	70319	Calmodulin (CaM)	1.5 (↑)	0.04	3	11.01%	23424.9	4.45
		**Translation initiation and elongation factors**						
236	102227	Elongation factor 1-beta	2.7 (↑)	0.11	3	14.85%	25127.97	4.4
24	90536	Elongation factor 2 (EF-2)	1.7 (−)	0.05	13	14.03%	93491.05	6.43
340	93992	Eukaryotic translation initiation factor 3 subunit F (eIF3f)	1.5 (↑)	0.08	2	6.68%	39992.56	4.79
		**ATP synthase**						
99	82931	ATP synthase subunit alpha, mitochondrial precursor	1.7 (−)	0.02	6	13.04%	59510.29	9.21
152	88657	ATP synthase subunit 5, mitochondrial precursor (Oligomycin sensitivity conferral protein) (OSCP) (ATP synthase chain 5)	1.5 (−)	0.12	5	19.03%	23687.24	9.25
		**Nascent polypeptide-associated complex**						
167	81890	Nascent polypeptide-associated complex subunit alpha (NAC-alpha) (Alpha-NAC)	2.3 (↑)	0.04	4	22.33%	22291.55	4.72
346	102329	Nascent polypeptide-associated complex subunit beta (NAC-beta) (Beta-NAC)	1.5 (↑)	0.04	2	14.74%	16945.14	5.81
		**Hydrolase activity**						
376	86663	Phosphatidyl inositol-specific phospholipase C, cplc1 (delta-type PLC)	2.2 (−)	0.03	2	2.31%	79759.42	6.22
303	103361	Aspergillopepsin	1.6 (↓)	0.07	2	8.08%	30918.72	3.75
302	108972	Carboxypeptidase S1	1.5 (↑)	0.11	2	3.72%	52979.56	4.09
		**Others**						
267	104299	Ubiquitin-conjugating enzyme E2 variant 1	1.7 (↑)	0.24	3	21.43%	17129.33	5.87
54	89402	Protein kinase C(CPKC)	1.5 (−)	0.03	10	11.92%	98880.94	5.24
338	103579	Guanine nucleotide-binding protein subunit beta-like protein (Cross-pathway control WD-repeat protein cpc-2)	1.5 (↑)	0.04	2	7.26%	35040.34	6.55
		**Unknown**						
337	81491	uncharacterized protein	3.0 (−)	0.06	2	2.03%	110226.61	4.95
188	105888	uncharacterized protein	2.5 (↑)	0.03	4	14.29%	31420.14	4.17
169	96317	uncharacterized protein	2.2 (↓)	0.06	4	24.51%	22018.9	5.58
423	61750	uncharacterized protein	2.2 (↓)	0.08	2	7.35%	29515.04	6.01
163	93265	uncharacterized protein	2.0 (−)	0.03	4	13.42%	31364.56	4.19
126	93693	uncharacterized protein	1.8 (↑)	0.03	5	20.90%	25408.27	5.4
160	90079	uncharacterized protein	1.8 (↓)	0.06	5	14.90%	28492.17	4.9
272	76370	uncharacterized protein	1.7 (−)	0.03	3	8.87%	31368.02	7.2
162	74162	uncharacterized protein	1.6 (↑)	0.03	5	12.85%	46952.71	5.87
477	102698	uncharacterized protein	1.6 (−)	0.07	1	14.46%	9204.46	6.17
110	40356	uncharacterized protein	1.5 (↓)	0.1	6	31.88%	24510.37	4.83
198	84988	uncharacterized protein	1.5 (↓)	0.17	4	17.41%	24885.25	5.05

^a^Accession number from *Cryphonectria parasitica* database v1.0.

^b^Hypovirus-free strain EP155 was set as reference. The average change (EP713/EP155) were from three independent experimental data. ‘(−)’, ‘(↑)’, ‘(↓)’ meant mRNA expression level of the corresponding gene was unchanged, up-regulated, down-regulated respectively upon hypovirus infection using high-throughput sequencing by Genome Analyzer IIx (Illumina).

^c^The total number of unique matched peptides to the identified protein.

^d^The cover percentage of matched peptides to the protein sequence.

^e^Theoretical molecular weight.

^f^Theoretical pI.

**Table 2:** List of down-regulated secreted proteins upon hypovirus infection identified by HPLC-ESI-OrbiTrap MSMS.

**No.**	Protein ID^a^	Protein Name	Average change (EP155/EP713)^b^	Standard error	Unique PepCount^c^	Cover Percent^d^	MW^e^	pI^f^
		**Hydrolase activity**						
172	36288	Endopolygalacturonase 1 precursor (Pectinase) (Clpg1)	3.7 (↑)	0.23	4	9.46%	37662.76	6.58
331	73874	Secreted aspartic proteinase	3.6 (↑)	0.3	2	3.55%	59382.36	4.07
487	95002	Endoglucanase-4 precursor (Endoglucanase IV) (Endo-1,4-beta-glucanase IV) (Cellulase IV) (EGIV)	3.0 (↓)	0.32	1	1.94%	36058.34	4.34
230	108053	Cutinase precursor (Cutin hydrolase)	2.8 (−)	0.42	3	18.42%	29225.54	4.19
492	69709	ATP-dependent DNA helicase II, 70 kDa subunit	2.8 (↓)	0.09	1	2.59%	39932.8	4.88
121	105629	Extracellular chitinase	2.4 (↑)	0.13	5	10.43%	48705.46	4.61
349	43693	Alpha-glucosidase precursor (Maltase)	2.4 (↓)	0.21	2	1.54%	111602.21	4.49
480	79318	3-phytase A precursor (Myo-inositol-hexaphosphate 3-phosphohydrolase A) (3 phytase A) (Myo-inositol hexakisphosphate phosphohydrolase A)	2.3 (↑)	0.42	1	2.08%	52879.6	4.82
315	107379	Beta-fructofuranosidase	2.0 (↓)	0.12	2	2.74%	62995.83	4.58
323	84745	Alpha amylase	2.0 (−)	0.08	2	3.17%	58630.95	4.19
327	99792	Extracellular cell wall glucanase	2.0 (↑)	0.36	2	9.92%	27691.24	4.11
165	67775	GPI-anchored cell wall beta-1,3-endoglucanase EglC	1.9 (↓)	0.07	4	18.16%	40242.32	4.41
233	107093	Endothiapepsin precursor (Aspartate protease)	1.6 (−)	0.13	3	6.63%	56426.06	4.43
305	103771	Survival protein sure-like phosphatase/nucleotidase-like protein	1.6 (↓)	0.08	2	7.79%	31859.39	4.05
304	65827	Polygalacturonase-3 precursor (Polygalacturonase III) (PG-III) (PGC) (Pectinase-3)	1.5 (−)	0.16	2	10.73%	36227.1	3.8
		**Oxidoreductase activity**						
474	39428	Short chain dehydrogenase/reductase family oxidoreductase	2.9 (↑)	0.2	1	2.75%	31682.42	6.53
26	106275	Alcohol dehydrogenase-1(ADH-1)	2.3 (↑)	0.1	12	38.70%	37278.81	6.15
66	39110	Cellobiose dehydrogenase	1.8 (−)	0.08	8	15.44%	61477.37	5.32
182	97515	Coproporphyrinogen III oxidase	1.8 (↓)	0.17	4	9.90%	46496.86	7.66
241	71516	Aspartate-semialdehyde dehydrogenase	1.7 (−)	0.2	3	7.12%	39149.09	6.2
10	101684	Glyceraldehyde-3-phosphate dehydrogenase (GAPDH)	1.5 (−)	0.03	18	59.76%	36191.85	6.46
101	64525	NADP-dependent leukotriene B4 12-hydroxydehydrogenase	1.5 (↑)	0.1	6	16.89%	31463.95	6.46
359	83754	Laccase	1.5 (↑)	0.09	2	3.99%	63159.75	4.36
388	104397	Putative oxidoreductase	1.5 (↑)	0.11	2	6.67%	39540.91	5.83
		**Transferase activity**						
345	48583	Serine hydroxymethyltransferase, cytosolic (Serine methylase) (Glycine hydroxymethyltransferase) (SHMT)	1.8 (−)	0.22	2	4.48%	51419.79	6.95
243	108828	1,3-beta-glucanosyltransferase gel2 precursor (Glucan elongating glucanosyltransferase 2)	1.9 (↓)	0.7	3	7.79%	48862	4
325	41127	Ribokinase-like protein	1.5 (↑)	0.17	2	6.80%	36364.05	5.1
		**Lypase/Esterase activity**						
119	105554	Extracellular lipase	2.5 (↑)	0.2	5	19.08%	37288.15	4.17
202	95432	Arylsulfatase	2.3 (−)	0.07	4	5.56%	69618.99	4.82
279	97118	Carboxylesterase	3.6 (↑)	0.19	3	8.23%	60638.58	4.36
		**Isomerase activity**						
56	55987	Peptidyl-prolyl cis-trans isomerase, mitochondrial precursor (PPIase) (Rotamase) (Cyclophilin) (Cyclosporin A-binding protein) (CPH)	2.1 (−)	0.07	9	43.41%	20099.5	7.81
244	58361	Peptidyl-prolyl cis-trans isomerase B precursor (PPIase B) (Rotamase B)	1.7 (−)	0.29	3	17.92%	18877.67	6.98
		**Lyase activity**						
75	104720	fructose bisphosphate aldolase	1.8 (↓)	0.24	7	22.71%	39353.73	5.52
		**Regulation factors**						
133	93073	mRNA binding post-transcriptional regulator	1.7 (−)	0.18	5	14.66%	41167.13	8.6
151	102966	14-3-3 protein	1.6 (↑)	0.02	5	20.51%	30396.05	4.9
142	40411	RHO protein GDP dissociation inhibitor(RDI) regulation of growth rate	1.5 (↑)	0.01	5	26.87%	22567.15	5.49
		**Ribosomal proteins**						
261	86511	Ribosomal protein L28e	2.4 (↑)	0.26	3	16.45%	16509.76	11
55	103787	Ubiquitin-40S ribosomal protein	1.8 (−)	0.02	9	45.81%	17687.54	9.85
		**Cytoskeleton**						
263	76520	Microtubule-associated protein RP/EB family member 1	1.9 (↑)	0.37	3	12.70%	27527.94	4.76
311	102150	Profilin	1.7 (−)	0.24	2	15.91%	13559.13	5.73
		**Unknown**						
322	107212	uncharacterized protein	2.9 (↓)	0.36	2	9.18%	29454.98	5.74
313	67753	uncharacterized protein	2.6 (↓)	0.16	2	8.33%	25705.21	4.28
232	74613	uncharacterized protein	2.5 (↓)	0.17	3	20.33%	18468.21	4.43
234	103448	uncharacterized protein	2.3 (↓)	0.16	3	13.83%	40306.61	4.8
100	95286	uncharacterized protein	1.9 (↓)	0.14	6	11.96%	51317.18	9.71
308	74957	uncharacterized protein	1.9 (−)	0.35	2	9.14%	36457.53	4.08
301	97441	uncharacterized protein	1.8 (−)	0.02	2	8.14%	35189.2	3.77
148	82357	uncharacterized protein	1.7 (↑)	0.25	5	17.65%	23990.76	6.65
312	104348	uncharacterized protein	1.7 (↓)	0.2	2	3.49%	74787.31	4.62
199	107223	uncharacterized protein	1.6 (↑)	0.22	4	6.58%	73713.63	4.49
207	70844	uncharacterized protein	1.5 (−)	0.23	4	20.20%	20542.29	4.92

^a^Accession number from *Cryphonectria parasitica* database v1.0.

^b^Hypovirus-free strain EP155 was set as reference. The average change (EP155/EP713) were from three independent experimental data. ‘(−)’, ‘(↑)’ and ‘(↓)’ mean mRNA expression level of the corresponding gene was unchanged, up-regulated, down-regulated respectively upon hypovirus infection using high-throughput sequencing by Genome Analyzer IIx (Illumina).

^c^The total number of unique matched peptides to the identified protein.

^d^The cover percentage of matched peptides to the protein sequence.

^e^Theoretical molecular weight.

^f^Theoretical pI.
