# Supplementary material for: Comparative Secretome Analysis Reveals Perturbation of Host Secretion Pathways by a Hypovirus
Source: Sci Rep. 2016 Oct 4;6:34308. doi: 10.1038/srep34308 (PMC5048421; doi:10.1038/srep34308)
Supplement: Supplementary Information [file srep34308-s1.doc]

**Comparative Secretome Analysis Reveals Perturbation of Host Secretion Pathways by a Hypovirus**

Jinzi Wang, Liming Shi, Xipu He, Lidan Lu, Xiaoping Li^§^ and Baoshan Chen*

State Key Laboratory for Conservation and Utilization of Subtropical Agro-bioresources and Key Laboratory for Microbial and Plant Genetic Engineering, Ministry of Education, College of Life Science and Technology, Guangxi University, Nanning 530004, China

^§^ Present address: Department of Biology, New Mexico State University, Las Cruces, NM 88003, USA

*Corresponding author. Address: State Key Laboratory for Conservation and Utilization of Subtropical Agro-bioresources, Guangxi University, Nanning, 530004, PR China

Tel: +86-771-3239566; Fax: +86-771-3237873; E-mail: [chenyaoj@gxu.edu.cn](mailto:chenyaoj@gxu.edu.cn)

Supplementary Figure S1 2-DE analysis of fungal secreted proteins.


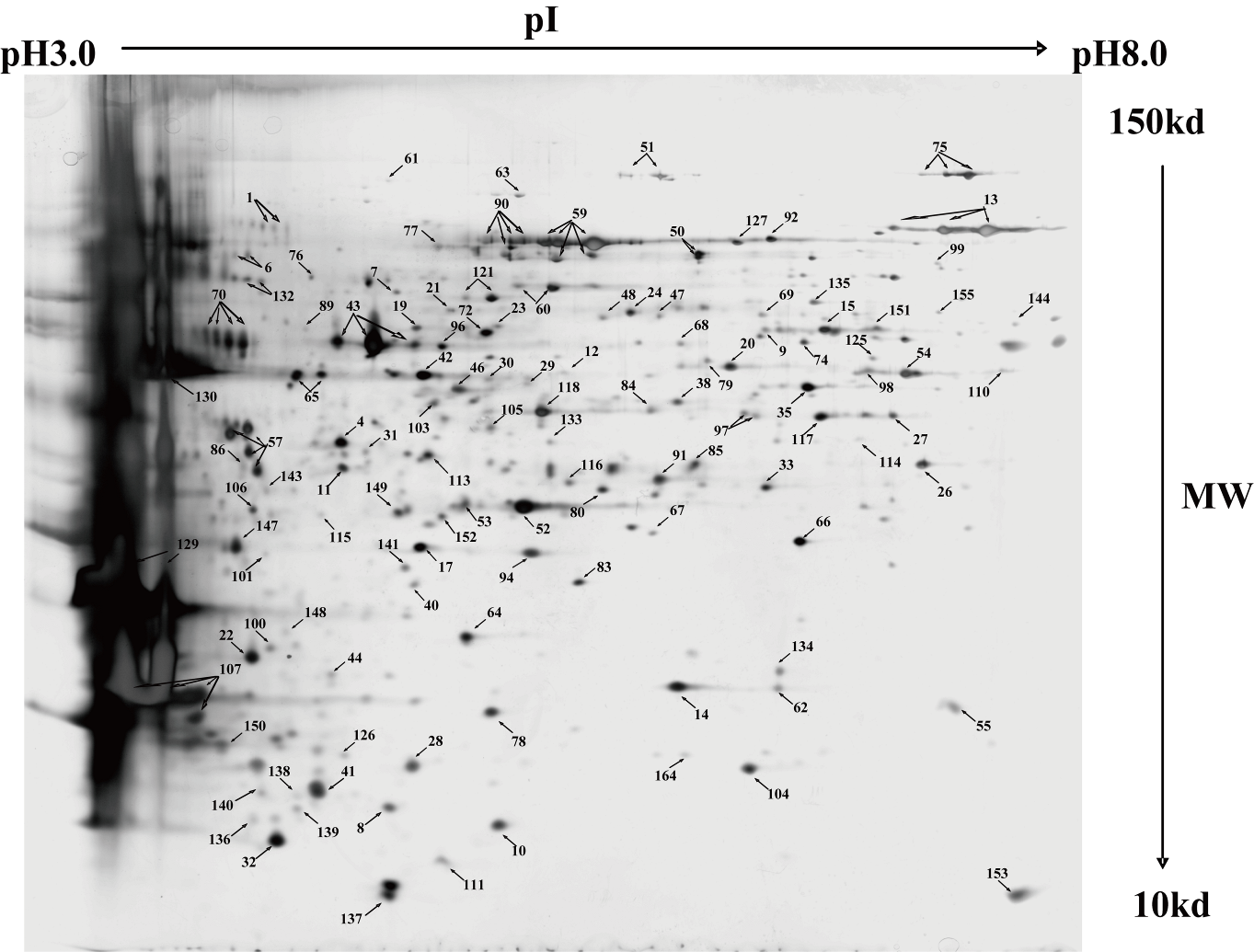


Supplementary Figure S2 2-DE analysis of fungal secretome with knocking out the gene encoding 22 kDa glycoprotein on day 3.


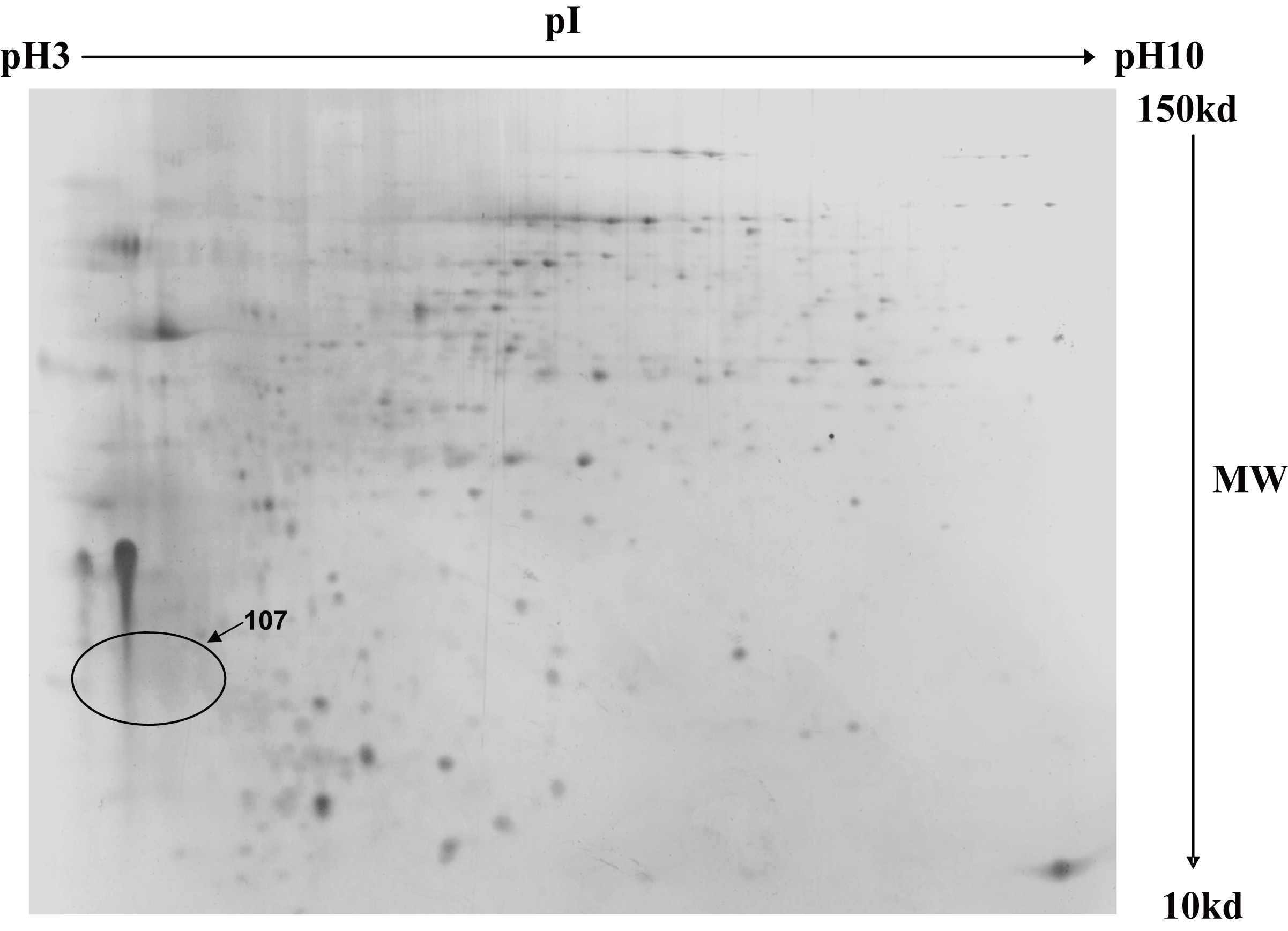


Supplementary Table S1 Identified secreted proteins in 2-DE analysis by MALDI-TOF/TOF.

a Spot numbers were marked on the 2D map (Supplemental Fig. 1).

b Accession number from Cryphonectria parasitica database v1.0.

c Predicted MW. The observed MW refered to Figure S-1.

d Predicted pI. The observed pI refered to Figure S-1.

e The criteria for acceptable identified result was greater than 95%.

* Laccase A was discovered in Day 5.

** The identified protein was just observed on 2D map and not included in iTRAQ identification.

| **Spot No.^a^** | **Protein Name** | **Accession No.^b^** | | **MW(kDa)^c^** | **pI^d^** | **Observed MW(kDa)** | **Observed pI** | **Peptide**  **Count** | **Protein Score**  **C. I. %^e^** |
| --- | --- | --- | --- | --- | --- | --- | --- | --- | --- |
| 1 | Aminopeptidase Y | | 99702 | 55 | 4.9 | 74 | 4.1 | 4 | 99.8 |
| 4 | Acetyltransferase, CysE/LacA/LpxA/NodL family | | 34384** | 26 | 5.0 | 40 | 4.5 | 11 | 100 |
| 6 | Dipeptidase | | 35302 | 46 | 5.0 | 68 | 4.0 | 12 | 99.9 |
| 7 | Thiamin pyrophosphokinase | | 36141 | 39 | 5.1 | 61 | 4.7 | 16 | 100 |
| 8 | uncharacterized protein | | 36895 | 13 | 5.1 | 14 | 4.8 | 7 | 100 |
| 9 151 | Alanine--glyoxylate aminotransferase 1 | | 38338 | 42 | 6.0 | 55, 55 | 6.7, 7.3 | 8 | 100 |
| 10 | Thioredoxin | | 39961 | 13 | 5.4 | 13 | 5.4 | 7 | 100 |
| 11 | uncharacterized protein | | 40356 | 24 | 4.8 | 37 | 4.5 | 15 | 100 |
| 12 | Methyltransferase | | 42151 | 36 | 5.9 | 49 | 5.7 | 14 | 100 |
| 13 | Pyruvate kinase | | 42922 | 58 | 6.5 | 71 | 8.0 | 20 | 100 |
| 14 | Peroxiredoxin | | 43719 | 18 | 6.1 | 19 | 6.3 | 6 | 99.8 |
| 15 | uncharacterized protein | | 43898 | 41 | 5.9 | 55 | 7.1 | 15 | 100 |
| 17 | O-methyltransferase | | 45332 | 24 | 5.6 | 29 | 5.0 | 8 | 100 |
| 19 | Aldose 1-epimerase | | 49385 | 36 | 5.2 | 55 | 4.9 | 9 | 100 |
| 20 | 2,3-dihydroxybenzoic acid decarboxylase | | 49665 | 40 | 5.8 | 49 | 6.5 | 13 | 100 |
| 21 148 | Peptidase family M20/M25/M40 | | 49897 | 65 | 5.2 | 58, 22 | 5.1, 4.2 | 4 | 99.9 |
| 22 | Endo-1,4-beta-xylanase A | | 50593** | 23 | 4.9 | 21 | 4.0 | 2 | 100 |
| 23 | uncharacterized protein | | 50954 | 39 | 5.4 | 56 | 5.4 | 11 | 100 |
| 24 | NADH:flavin oxidoreductase/NADH oxidase family protein | | 50968 | 44 | 5.6 | 59 | 6.3 | 23 | 100 |
| 26 | Ribose 5-phosphate isomerase-like protein | | 55273 | 29 | 6.5 | 37 | 7.5 | 6 | 99.5 |
| 27 | NADPH-dependent D-xylose reductase II,III | | 55348 | 36 | 6.2 | 43 | 7.4 | 8 | 100 |
| 28 | Protein HMF1 | | 55727 | 13 | 5.4 | 16 | 4.9 | 3 | 99.1 |
| 29 30 31 | MYG1 protein | | 57041 | 40 | 5.5 | 48, 48, 39 | 5.5, 5.3, 4.6 | 18 | 100 |
| 32 | Nuclear transport factor 2 (NTF-2) | | 57583 | 14 | 5.0 | 13 | 4.1 | 4 | 100 |
| 33 | S-formylglutathione hydrolase | | 59013 | 30 | 5.5 | 35 | 6.7 | 8 | 100 |
| 35 | Aldo/keto reductase | | 63094 | 37 | 6.0 | 47 | 6.9 | 7 | 100 |
| 40 41 136 138 140 | Peroxiredoxin (AsPrx) (Thioredoxin peroxidase) | | 68536 | 22 | 5.3 | 26, 14, 13, 14, 14 | 4.9, 4.3, 4.0, 4.2, 4.0 | 6 | 100 |
| 42 43 | Pectin lyase A | | 69046 | 40 | 5.3 | 48, 53 | 5.0, 4.7 | 3 | 100 |
| 44 | uncharacterized protein | | 70589 | 17 | 5.0 | 20 | 4.4 | 9 | 100 |
| 46 | Thioredoxin reductase | | 72159 | 36 | 5.3 | 46 | 5.2 | 11 | 100 |
| 47 48 | 2-ketoglutarate-dependent dioxygenase | | 74162 | 47 | 5.9 | 58, 57 | 6.2, 5.9 | 14 | 99.5 |
| 150 | hypothetical protein | | 74613 | 18 | 4.4 | 16 | 3.7 | 1 | 100 |
| 50 51 135 155 | Catalase/peroxidase | | 76127 | 83 | 5.6 | 68, 86, 60, 61 | 6.4, 6.2, 6.9, 7.7 | 13 | 100 |
| 52 53 149 152 | Triosephosphate isomerase (TPI) | | 77119 | 27 | 5.4 | 33, 33, 32, 32 | 5.6, 5.2, 4.8, 5.1 | 5 | 99.3 |
| 54 | NADPH:quinone reductase and related Zn-dependent oxidoreductases | | 77940 | 36 | 6.5 | 48 | 7.4 | 13 | 100 |
| 55 | Nucleoside diphosphate kinase-1 (NDK-1) | | 78037 | 17 | 6.8 | 18 | 7.7 | 6 | 100 |
| 57 | HSP70-1 | | 79019 | 71 | 5.0 | 39 | 4.0 | 5 | 100 |
| 59 | Pyruvate decarboxylase (8-10 nm cytoplasmic filament-associated protein) | | 79606 | 62 | 5.6 | 71 | 5.7 | 13 | 100 |
| 60 | Rab GDP-dissociation inhibitor | | 80153 | 51 | 5.6 | 62 | 5.7 | 17 | 100 |
| 61 | Heat shock protein Hsp88 | | 80500 | 80 | 5.1 | 85 | 4.8 | 20 | 100 |
| 62 | superoxide dismutase [Cu-Zn] SOD | | 80754 | 16 | 5.9 | 19 | 6.8 | 7 | 100 |
| 63 | Dipeptidyl peptidase | | 81065 | 78 | 5.4 | 81 | 5.5 | 25 | 100 |
| 64 | uncharacterized protein | | 81074** | 20 | 5.4 | 22 | 5.2 | 9 | 100 |
| 65 | Aldose-1-epimerase | | 81476 | 34 | 4.9 | 48 | 4.2 | 15 | 100 |
| 66 67 | 6,7-dimethyl-8-ribityllumazine synthase (DMRL synthase) (Lumazine synthase) (Riboflavin synthase beta chain) | | 82588 | 21 | 5.4 | 30, 30 | 6.9, 6.1 | 3 | 97.2 |
| 68 | Aminopeptidase 2 | | 82725 | 98 | 5.3 | 53 | 6.3 | 26 | 100 |
| 69 | Transketolase | | 83897 | 75 | 5.7 | 57 | 6.7 | 13 | 100 |
| 70 | Vacuolar protease A precursor (PEP-4) | | 83961 | 43 | 4.8 | 53 | 3.9 | 13 | 100 |
| 72 | Sec14 cytosolic factor | | 85234 | 39 | 5.4 | 54 | 5.3 | 16 | 100 |
| 74 | formate dehydrogenase | | 87808 | 48 | 8.5 | 53 | 6.9 | 12 | 100 |
| 75 144 | Methionine synthase | | 88329 | 86 | 6.3 | 86, 56 | 7.8, 8.2 | 18 | 100 |
| 76 | ATP synthase subunit beta, mitochondrial precursor | | 88762 | 55 | 5.3 | 64 | 4.3 | 13 | 100 |
| 77 | Heat shock protein 60 | | 88770 | 61 | 5.6 | 70 | 5.0 | 15 | 100 |
| 78 | Cofilin (Actin-depolymerizing factor 1) | | 88774 | 17 | 5.5 | 18 | 5.4 | 4 | 100 |
| 79 | UDP-glucose 4-epimerase | | 89596 | 41 | 5.8 | 50 | 6.4 | 15 | 100 |
| 80 | Cyanamide hydratase | | 89696** | 27 | 5.6 | 35 | 5.9 | 9 | 100 |
| 83 | 1,4-benzoquinone reductase | | 91352 | 22 | 5.5 | 26 | 5.8 | 6 | 100 |
| 84 | uncharacterized protein | | 91966 | 37 | 5.7 | 44 | 6.1 | 20 | 100 |
| 85 | uncharacterized protein | | 92044 | 25 | 5.8 | 37 | 6.3 | 9 | 100 |
| 86 | Glutamate carboxypeptidase | | 93457 | 52 | 5.2 | 37 | 3.9 | 12 | 100 |
| 89 | eukaryotic translation initiation factor 3 subunit F | | 93992 | 40 | 4.8 | 56 | 4.2 | 9 | 100 |
| 90 133 | Phosphoglycerate mutase, 2,3-bisphosphoglycerate-independent | | 94119 | 58 | 5.5 | 69, 40 | 5.3, 5.6 | 10 | 99.7 |
| 91 | L-xylulose reductase (XR) | | 94341 | 28 | 5.7 | 36 | 6.2 | 8 | 100 |
| 92 | Amidohydrolase | | 94542 | 57 | 6.0 | 72 | 6.2 | 20 | 100 |
| 94 | Elongation factor 1-gamma | | 95810 | 47 | 6.1 | 29 | 5.5 | 5 | 99.4 |
| 96 | 3-isopropylmalate dehydrogenase (Beta-IPM dehydrogenase) (IMDH) (3-IPM-DH) | | 96560 | 39 | 5.3 | 52 | 5.1 | 8 | 100 |
| 97 | probable coproporphyrinogen III oxidase | | 97515 | 46 | 7.7 | 43 | 6.7 | 15 | 100 |
| 98 | Alcohol dehydrogenase | | 97558 | 38 | 6.2 | 49 | 7.2 | 16 | 100 |
| 99 | Inosine-5'-monophosphate dehydrogenase | | 98458 | 57 | 6.2 | 67 | 7.6 | 14 | 100 |
| 100 101 105 | Inorganic pyrophosphatase (Pyrophosphate phospho-hydrolase) (PPase) | | 98830 | 33 | 5.4 | 22, 29, 41 | 4.1, 4.1, 5.3 | 4 | 99.7 |
| 103 | Arginase | | 99673 | 38 | 5.6 | 44 | 5.0 | 8 | 100 |
| 104 | Ubiquitin-conjugating enzyme E2-16 kDa | | 99736** | 44 | 5.2 | 15 | 6.6 | 9 | 98.3 |
| 106 132 | Endothiapepsin precursor (Aspartate protease) | | 100383 | 43 | 4.8 | 33, 64 | 4.0, 4.0 | 5 | 100 |
| 107 | 22kDa glycoprotein | | 100735 | 14 | 4.4 | 19 | 3.7 | 2 | 100 |
| 111 | uncharacterized protein | | 101944 | 12 | 5.5 | 12 | 5.1 | 8 | 100 |
| 113 | Ubiquinone/menaquinone biosynthesis methyltransferase | | 102189 | 31 | 5.2 | 38 | 5.0 | 10 | 100 |
| 114 | uncharacterized protein | | 102261 | 35 | 6.1 | 40 | 7.2 | 23 | 100 |
| 115 139 | uncharacterized protein | | 102285 | 23 | 5.0 | 32, 14 | 4.4, 4.2 | 3 | 100 |
| 116 | Rossmann-fold NAD(P)(+)-binding protein | | 102665 | 28 | 5.5 | 35 | 5.7 | 13 | 100 |
| 117 | Subtilisin-like proteinase | | 102797 | 55 | 5.3 | 43 | 7.0 | 6 | 100 |
| 118 | NAD(P)H-dependent D-xylose reductase (XR) | | 103071 | 36 | 5.4 | 43 | 5.6 | 15 | 100 |
| 121 | Enolase | | 103155 | 47 | 5.3 | 60 | 5.3 | 17 | 100 |
| 125 | alcohol dehydrogenase 1 | | 106275 | 37 | 6.2 | 51 | 7.3 | 8 | 100 |
| 126 | uncharacterized protein | | 106534 | 23 | 6.0 | 16 | 4.5 | 4 | 100 |
| 127 | UDP-N-acetylglucosamine pyrophosphorylase | | 107213 | 56 | 5.9 | 71 | 6.5 | 13 | 100 |
| 129 | Cell wall protein PhiA | | 103814 | 19 | 4.1 | 25 | 3.2 | 1 | 100 |
| 130 | Cell wall glucanase | | 67775 | 40 | 4.4 | 49 | 3.6 | 3 | 98.2 |
| 130 | mono- and diacylglycerol lipase | | 105554 | 37 | 4.2 | 49 | 3.6 | 4 | 100 |
| 130 | Transaldolase | | 42419 | 36 | 5.3 | 49 | 3.6 | 7 | 99.2 |
| 132 | FAD binding domain-containing protein | | 109500 | 51 | 4.3 | 64 | 4.0 | 7 | 100 |
| 133 | uncharacterized protein | | 58481 | 57 | 5.4 | 40 | 5.6 | 11 | 99.8 |
| 134 153 | Rho GDP-dissociation inhibitor | | 40411 | 23 | 5.5 | 20, 11 | 6.8, 8.4 | 7 | 100 |
| 137 | 3-phytase A precursor | | 79318 | 58 | 4.3 | 11 | 4.8 | 8 | 100 |
| 141 | Nitroreductase | | 96894 | 22 | 5.4 | 28 | 4.9 | 5 | 98.3 |
| 143 | Vacuolar aminopeptidase 1 | | 34225 | 53 | 6.1 | 34 | 4.1 | 10 | 98.6 |
| 147 | Translationally-controlled tumor protein homolog (TCTP) | | 77315 | 19 | 4.5 | 29 | 3.9 | 8 | 100 |
| 147 | uncharacterized protein | | 105888 | 31 | 4.2 | 29 | 3.9 | 6 | 100 |
| 148 | uncharacterized protein | | 80289 | 36 | 5.6 | 22 | 4.2 | 4 | 100 |
| 150 | predicted protein | | 103448 | 40 | 4.8 | 16 | 3.9 | 3 | 99.8 |
| 164 | 14-3-3-like protein | | 102966 | 30 | 5.0 | 16 | 6.2 | 7 | 100 |
| * | Laccase A | | 69386** | 65 | 5.5 | 70 | 4.0-5.5 | 7 | 100 |

Supplemental Table 2 iTRAQ quantification of differentially expressed secreted proteins in *C. parasitica* strains

| CP Number | Pep Count | Unique Pep Count | Cover Percent  % | MW | PI | EP713:EP155 | | |  |  |
| --- | --- | --- | --- | --- | --- | --- | --- | --- | --- | --- |
|  |  |  |  |  |  | Exp 1 | Exp 2 | Exp 3 | x | SD |
| jgi|Crypa1|36288|e_gw1.1.966.1 | 26 | 4 | 9.46 | 37662.76 | 6.58 | 4.06 | 3.13 | 3.95 | 3.71 | 0.51 |
| jgi|Crypa1|73874|fgenesh1_pg.C_scaffold_8000231 | 11 | 2 | 3.55 | 59382.36 | 4.07 | 3.19 | 4.21 | 3.35 | 3.58 | 0.55 |
| jgi|Crypa1|97118|estExt_fgenesh1_pm.C_11207 | 8 | 3 | 8.23 | 60638.58 | 4.36 | 1.46 | 4.28 | 4.99 | 3.58 | 1.87 |
| jgi|Crypa1|95002|estExt_Genewise1Plus.C_90821 | 6 | 1 | 1.94 | 36058.34 | 4.34 | 3.02 | 3.58 | 2.49 | 3.03 | 0.55 |
| jgi|Crypa1|107212|estExt_fgenesh1_pg.C_40590 | 13 | 2 | 9.18 | 29454.98 | 5.74 | 4.10 | 2.75 | 1.91 | 2.92 | 1.11 |
| jgi|Crypa1|39428|e_gw1.3.894.1 | 11 | 1 | 2.75 | 31682.42 | 6.53 | 2.80 | 2.85 | 2.93 | 2.86 | 0.06 |
| jgi|Crypa1|108053|estExt_fgenesh1_pg.C_60669 | 242 | 3 | 18.42 | 29225.54 | 4.19 | 2.25 | 3.77 | 2.46 | 2.83 | 0.83 |
| jgi|Crypa1|69709|fgenesh1_pg.C_scaffold_3000775 | 5 | 1 | 2.59 | 39932.80 | 4.88 | 2.96 | 2.69 | 2.66 | 2.77 | 0.17 |
| jgi|Crypa1|67753|fgenesh1_pg.C_scaffold_2000028 | 22 | 2 | 8.33 | 25705.21 | 4.28 | 2.61 | 2.76 | 2.41 | 2.59 | 0.18 |
| jgi|Crypa1|105554|estExt_fgenesh1_pg.C_11349 | 98 | 5 | 19.08 | 37288.15 | 4.17 | 2.90 | 2.15 | 2.60 | 2.55 | 0.38 |
| jgi|Crypa1|74613|fgenesh1_pg.C_scaffold_9000328 | 205 | 3 | 20.33 | 18468.21 | 4.43 | 2.76 | 2.17 | 2.62 | 2.52 | 0.31 |
| jgi|Crypa1|86511|estExt_Genewise1Plus.C_10503 | 11 | 3 | 16.45 | 16509.76 | 11.00 | 1.91 | 3.11 | 2.28 | 2.43 | 0.61 |
| jgi|Crypa1|43693|e_gw1.4.1056.1 | 8 | 2 | 1.54 | 111602.21 | 4.49 | 1.46 | 3.71 | 2.07 | 2.41 | 1.17 |
| jgi|Crypa1|105629|estExt_fgenesh1_pg.C_11497 | 89 | 5 | 10.43 | 48705.46 | 4.61 | 2.48 | 2.18 | 2.45 | 2.37 | 0.16 |
| jgi|Crypa1|103448|estExt_fgenesh1_kg.C_70130 | 90 | 3 | 13.83 | 40306.61 | 4.80 | 2.67 | 2.38 | 1.94 | 2.33 | 0.37 |
| jgi|Crypa1|106275|estExt_fgenesh1_pg.C_21105 | 502 | 12 | 38.70 | 37278.81 | 6.15 | 2.19 | 2.34 | 2.42 | 2.32 | 0.12 |
| jgi|Crypa1|95432|estExt_Genewise1Plus.C_100336 | 14 | 4 | 5.56 | 69618.99 | 4.82 | 2.09 | 2.19 | 2.62 | 2.30 | 0.28 |
| jgi|Crypa1|79318|estExt_Genewise1.C_30066 | 7 | 1 | 2.08 | 52879.60 | 4.82 | 1.84 | 1.87 | 3.14 | 2.29 | 0.74 |
| jgi|Crypa1|55987|fgenesh1_kg.C_scaffold_4000446 | 127 | 9 | 43.41 | 20099.50 | 7.81 | 2.07 | 2.18 | 1.97 | 2.08 | 0.10 |
| jgi|Crypa1|107379|estExt_fgenesh1_pg.C_41002 | 18 | 2 | 2.74 | 62995.83 | 4.58 | 1.78 | 2.10 | 2.17 | 2.02 | 0.21 |
| jgi|Crypa1|99792|estExt_fgenesh1_pm.C_90078 | 12 | 2 | 9.92 | 27691.24 | 4.11 | 1.69 | 2.16 | 2.09 | 1.98 | 0.25 |
| jgi|Crypa1|84745|estExt_Genewise1.C_90496 | 13 | 2 | 3.17 | 58630.95 | 4.19 | 2.70 | 1.78 | 1.40 | 1.96 | 0.67 |
| jgi|Crypa1|76520|estExt_Genewise1.C_10605 | 11 | 3 | 12.70 | 27527.94 | 4.76 | 1.51 | 2.72 | 1.61 | 1.95 | 0.67 |
| jgi|Crypa1|74957|fgenesh1_pg.C_scaffold_9000672 | 30 | 2 | 9.14 | 36457.53 | 4.08 | 2.28 | 1.44 | 1.98 | 1.90 | 0.43 |
| jgi|Crypa1|108828|estExt_fgenesh1_pg.C_90241 | 16 | 3 | 7.79 | 48862.00 | 4.00 | 1.27 | 1.51 | 2.80 | 1.86 | 0.82 |
| jgi|Crypa1|95286|estExt_Genewise1Plus.C_100071 | 36 | 6 | 11.96 | 51317.18 | 9.71 | 1.80 | 1.72 | 2.05 | 1.86 | 0.17 |
| jgi|Crypa1|67775|fgenesh1_pg.C_scaffold_2000050 | 44 | 4 | 18.16 | 40242.32 | 4.41 | 1.88 | 1.75 | 1.93 | 1.85 | 0.09 |
| jgi|Crypa1|97441|estExt_fgenesh1_pm.C_20476 | 88 | 2 | 8.14 | 35189.20 | 3.77 | 2.21 | 1.54 | 1.78 | 1.85 | 0.34 |
| jgi|Crypa1|39110|e_gw1.3.408.1 | 84 | 8 | 15.44 | 61477.37 | 5.32 | 1.96 | 1.89 | 1.65 | 1.83 | 0.16 |
| jgi|Crypa1|97515|estExt_fgenesh1_pm.C_20606 | 18 | 4 | 9.90 | 46496.86 | 7.66 | 1.89 | 1.31 | 2.30 | 1.83 | 0.49 |
| jgi|Crypa1|103787|estExt_fgenesh1_kg.C_80170 | 276 | 9 | 45.81 | 17687.54 | 9.85 | 1.75 | 1.91 | 1.80 | 1.82 | 0.08 |
| jgi|Crypa1|48583|e_gw1.8.948.1 | 8 | 2 | 4.48 | 51419.79 | 6.95 | 2.12 | 1.82 | 1.39 | 1.78 | 0.37 |
| jgi|Crypa1|104720|estExt_fgenesh1_kg.C_140033 | 423 | 7 | 22.71 | 39353.73 | 5.52 | 2.37 | 1.76 | 1.17 | 1.77 | 0.60 |
| jgi|Crypa1|102150|estExt_fgenesh1_kg.C_30445 | 25 | 2 | 15.91 | 13559.13 | 5.73 | 1.55 | 2.11 | 1.59 | 1.75 | 0.32 |
| jgi|Crypa1|93073|estExt_Genewise1Plus.C_61164 | 23 | 5 | 14.66 | 41167.13 | 8.60 | 1.65 | 1.67 | 1.90 | 1.74 | 0.14 |
| jgi|Crypa1|58361|fgenesh1_kg.C_scaffold_13000012 | 16 | 3 | 17.92 | 18877.67 | 6.98 | 2.01 | 1.83 | 1.28 | 1.71 | 0.38 |
| jgi|Crypa1|71516|fgenesh1_pg.C_scaffold_5000139 | 18 | 3 | 7.12 | 39149.09 | 6.20 | 2.02 | 1.43 | 1.66 | 1.71 | 0.30 |
| jgi|Crypa1|104348|estExt_fgenesh1_kg.C_90466 | 22 | 2 | 3.49 | 74787.31 | 4.62 | 1.67 | 1.83 | 1.59 | 1.70 | 0.12 |
| jgi|Crypa1|82357|estExt_Genewise1.C_51242 | 16 | 5 | 17.65 | 23990.76 | 6.65 | 1.83 | 1.34 | 1.88 | 1.68 | 0.30 |
| jgi|Crypa1|107223|estExt_fgenesh1_pg.C_40620 | 14 | 4 | 6.58 | 73713.63 | 4.49 | 1.30 | 1.57 | 2.03 | 1.63 | 0.37 |
| jgi|Crypa1|103771|estExt_fgenesh1_kg.C_80154 | 51 | 2 | 7.79 | 31859.39 | 4.05 | 1.44 | 1.81 | 1.66 | 1.63 | 0.19 |
| jgi|Crypa1|107093|estExt_fgenesh1_pg.C_40368 | 132 | 3 | 6.63 | 56426.06 | 4.43 | 1.80 | 1.58 | 1.33 | 1.57 | 0.24 |
| jgi|Crypa1|102966|estExt_fgenesh1_kg.C_50321 | 15 | 5 | 20.51 | 30396.05 | 4.90 | 1.32 | 1.79 | 1.59 | 1.57 | 0.24 |
| jgi|Crypa1|83754|estExt_Genewise1.C_71148 | 7 | 2 | 3.99 | 63159.75 | 4.36 | 1.70 | 1.54 | 1.40 | 1.55 | 0.15 |
| jgi|Crypa1|101684|estExt_fgenesh1_kg.C_20528 | 1239 | 18 | 59.76 | 36191.85 | 6.46 | 1.46 | 1.52 | 1.63 | 1.54 | 0.09 |
| jgi|Crypa1|70844|fgenesh1_pg.C_scaffold_4000585 | 11 | 4 | 20.20 | 20542.29 | 4.92 | 1.06 | 1.91 | 1.60 | 1.52 | 0.43 |
| jgi|Crypa1|64525|fgenesh1_pm.C_scaffold_8000312 | 34 | 6 | 16.89 | 31463.95 | 6.46 | 1.45 | 1.51 | 1.53 | 1.50 | 0.04 |
| jgi|Crypa1|104397|estExt_fgenesh1_kg.C_90522 | 5 | 2 | 6.67 | 39540.91 | 5.83 | 1.14 | 1.79 | 1.54 | 1.49 | 0.33 |
| jgi|Crypa1|41127|e_gw1.3.813.1 | 12 | 2 | 6.80 | 36364.05 | 5.10 | 1.32 | 1.77 | 1.30 | 1.46 | 0.27 |
| jgi|Crypa1|65827|fgenesh1_pm.C_scaffold_17000019 | 58 | 2 | 10.73 | 36227.10 | 3.80 | 1.29 | 1.82 | 1.26 | 1.46 | 0.32 |
| jgi|Crypa1|40411|e_gw1.3.1006.1 | 19 | 5 | 26.87 | 22567.15 | 5.49 | 1.43 | 1.39 | 1.55 | 1.45 | 0.08 |
| jgi|Crypa1|82952|estExt_Genewise1.C_61103 | 10 | 5 | 4.14 | 130792.42 | 6.15 | 0.73 | 0.70 | 0.62 | 0.69 | 0.06 |
| jgi|Crypa1|107257|estExt_fgenesh1_pg.C_40699 | 6 | 4 | 13.49 | 37374.71 | 9.00 | 0.60 | 0.87 | 0.59 | 0.68 | 0.16 |
| jgi|Crypa1|103579|estExt_fgenesh1_kg.C_70279 | 9 | 2 | 7.26 | 35040.34 | 6.55 | 0.76 | 0.65 | 0.64 | 0.68 | 0.07 |
| jgi|Crypa1|93992|estExt_Genewise1Plus.C_71390 | 9 | 2 | 6.68 | 39992.56 | 4.79 | 0.75 | 0.74 | 0.55 | 0.68 | 0.11 |
| jgi|Crypa1|70319|fgenesh1_pg.C_scaffold_4000060 | 19 | 3 | 11.01 | 23424.90 | 4.45 | 0.70 | 0.76 | 0.58 | 0.68 | 0.09 |
| jgi|Crypa1|87102|estExt_Genewise1Plus.C_11634 | 15 | 4 | 15.49 | 33212.42 | 6.01 | 0.50 | 0.76 | 0.77 | 0.68 | 0.15 |
| jgi|Crypa1|88657|estExt_Genewise1Plus.C_21031 | 14 | 5 | 19.03 | 23687.24 | 9.25 | 0.90 | 0.40 | 0.73 | 0.68 | 0.26 |
| jgi|Crypa1|84988|estExt_Genewise1.C_91076 | 14 | 4 | 17.41 | 24885.25 | 5.05 | 0.92 | 0.38 | 0.71 | 0.67 | 0.27 |
| jgi|Crypa1|102329|estExt_fgenesh1_kg.C_40095 | 8 | 2 | 14.74 | 16945.14 | 5.81 | 0.59 | 0.75 | 0.66 | 0.67 | 0.08 |
| jgi|Crypa1|89402|estExt_Genewise1Plus.C_30081 | 26 | 10 | 11.92 | 98880.94 | 5.24 | 0.62 | 0.62 | 0.76 | 0.67 | 0.08 |
| jgi|Crypa1|40356|e_gw1.3.2793.1 | 17 | 6 | 31.88 | 24510.37 | 4.83 | 0.80 | 0.76 | 0.41 | 0.66 | 0.22 |
| jgi|Crypa1|108972|estExt_fgenesh1_pg.C_90583 | 76 | 2 | 3.72 | 52979.56 | 4.09 | 0.79 | 0.61 | 0.57 | 0.65 | 0.12 |
| jgi|Crypa1|42588|e_gw1.4.376.1 | 39 | 12 | 20.70 | 57401.52 | 6.19 | 0.86 | 0.49 | 0.57 | 0.64 | 0.20 |
| jgi|Crypa1|99412|estExt_fgenesh1_pm.C_70308 | 61 | 13 | 29.49 | 57358.20 | 6.38 | 0.56 | 0.81 | 0.56 | 0.64 | 0.15 |
| jgi|Crypa1|80754|estExt_Genewise1.C_40211 | 143 | 5 | 37.42 | 15945.45 | 5.85 | 0.58 | 0.70 | 0.63 | 0.63 | 0.06 |
| jgi|Crypa1|87808|estExt_Genewise1Plus.C_13013 | 70 | 10 | 21.56 | 48658.25 | 8.65 | 0.56 | 0.70 | 0.63 | 0.63 | 0.07 |
| jgi|Crypa1|103140|estExt_fgenesh1_kg.C_60111 | 13 | 3 | 9.55 | 33639.19 | 4.62 | 0.84 | 0.54 | 0.51 | 0.63 | 0.18 |
| jgi|Crypa1|58903|fgenesh1_pm.C_scaffold_1000140 | 6 | 2 | 3.64 | 51778.91 | 5.94 | 0.59 | 0.57 | 0.71 | 0.63 | 0.08 |
| jgi|Crypa1|85870|estExt_Genewise1.C_140218 | 6 | 2 | 3.11 | 77900.66 | 6.19 | 0.60 | 0.43 | 0.85 | 0.62 | 0.21 |
| jgi|Crypa1|74162|fgenesh1_pg.C_scaffold_8000519 | 9 | 5 | 12.85 | 46952.71 | 5.87 | 0.64 | 0.55 | 0.65 | 0.61 | 0.06 |
| jgi|Crypa1|54024|fgenesh1_kg.C_scaffold_1000609 | 8 | 2 | 31.34 | 7608.81 | 10.37 | 0.47 | 0.79 | 0.57 | 0.61 | 0.16 |
| jgi|Crypa1|102698|estExt_fgenesh1_kg.C_50011 | 8 | 1 | 14.46 | 9204.46 | 6.17 | 0.58 | 0.61 | 0.65 | 0.61 | 0.03 |
| jgi|Crypa1|103361|estExt_fgenesh1_kg.C_70037 | 66 | 2 | 8.08 | 30918.72 | 3.75 | 0.66 | 0.27 | 0.90 | 0.61 | 0.32 |
| jgi|Crypa1|99052|estExt_fgenesh1_pm.C_60226 | 29 | 11 | 26.88 | 55317.38 | 4.69 | 0.60 | 0.67 | 0.55 | 0.60 | 0.06 |
| jgi|Crypa1|91352|estExt_Genewise1Plus.C_41216 | 27 | 4 | 19.51 | 21569.41 | 5.46 | 0.60 | 0.65 | 0.55 | 0.60 | 0.05 |
| jgi|Crypa1|90536|estExt_Genewise1Plus.C_32373 | 43 | 13 | 14.03 | 93491.05 | 6.43 | 0.59 | 0.50 | 0.69 | 0.60 | 0.09 |
| jgi|Crypa1|82931|estExt_Genewise1.C_61046 | 49 | 6 | 13.04 | 59510.29 | 9.21 | 0.51 | 0.55 | 0.72 | 0.60 | 0.11 |
| jgi|Crypa1|101628|estExt_fgenesh1_kg.C_20460 | 35 | 5 | 19.30 | 31041.90 | 4.76 | 0.64 | 0.54 | 0.60 | 0.59 | 0.05 |
| jgi|Crypa1|103417|estExt_fgenesh1_kg.C_70096 | 25 | 4 | 29.09 | 11647.55 | 9.77 | 0.73 | 0.40 | 0.64 | 0.59 | 0.17 |
| jgi|Crypa1|104299|estExt_fgenesh1_kg.C_90404 | 10 | 3 | 21.43 | 17129.33 | 5.87 | 0.68 | 0.30 | 0.77 | 0.59 | 0.25 |
| jgi|Crypa1|76370|estExt_Genewise1.C_10308 | 9 | 3 | 8.87 | 31368.02 | 7.20 | 0.57 | 0.51 | 0.68 | 0.58 | 0.09 |
| jgi|Crypa1|55348|fgenesh1_kg.C_scaffold_3000361 | 7 | 1 | 3.07 | 36430.60 | 6.15 | 0.35 | 0.88 | 0.46 | 0.56 | 0.28 |
| jgi|Crypa1|90732|estExt_Genewise1Plus.C_40039 | 4 | 2 | 13.16 | 16681.79 | 9.18 | 0.57 | 0.51 | 0.61 | 0.56 | 0.05 |
| jgi|Crypa1|100785|estExt_fgenesh1_kg.C_10390 | 13 | 5 | 39.74 | 16045.36 | 10.68 | 0.61 | 0.61 | 0.46 | 0.56 | 0.09 |
| jgi|Crypa1|79283|estExt_Genewise1.C_22318 | 19 | 6 | 28.84 | 23791.27 | 9.28 | 0.67 | 0.43 | 0.59 | 0.56 | 0.12 |
| jgi|Crypa1|90079|estExt_Genewise1Plus.C_31506 | 11 | 5 | 14.90 | 28492.17 | 4.90 | 0.43 | 0.73 | 0.51 | 0.55 | 0.15 |
| jgi|Crypa1|93693|estExt_Genewise1Plus.C_70815 | 36 | 5 | 20.90 | 25408.27 | 5.40 | 0.44 | 0.61 | 0.59 | 0.55 | 0.09 |
| jgi|Crypa1|93265|estExt_Genewise1Plus.C_61603 | 415 | 4 | 13.42 | 31364.56 | 4.19 | 0.45 | 0.54 | 0.49 | 0.50 | 0.05 |
| jgi|Crypa1|96317|estExt_Genewise1Plus.C_170130 | 30 | 4 | 24.51 | 22018.90 | 5.58 | 0.59 | 0.66 | 0.14 | 0.46 | 0.28 |
| jgi|Crypa1|75598|fgenesh1_pg.C_scaffold_13000052 | 26 | 5 | 34.34 | 17645.42 | 9.41 | 0.49 | 0.46 | 0.43 | 0.46 | 0.03 |
| jgi|Crypa1|86663|estExt_Genewise1Plus.C_10815 | 6 | 2 | 2.31 | 79759.42 | 6.22 | 0.47 | 0.51 | 0.39 | 0.46 | 0.06 |
| jgi|Crypa1|61750|fgenesh1_pm.C_scaffold_3000844 | 3 | 2 | 7.35 | 29515.04 | 6.01 | 0.60 | 0.34 | 0.42 | 0.46 | 0.14 |
| jgi|Crypa1|81890|estExt_Genewise1.C_50315 | 36 | 4 | 22.33 | 22291.55 | 4.72 | 0.44 | 0.49 | 0.38 | 0.44 | 0.05 |
| jgi|Crypa1|105888|estExt_fgenesh1_pg.C_20285 | 16 | 4 | 14.29 | 31420.14 | 4.17 | 0.37 | 0.44 | 0.41 | 0.40 | 0.03 |
| jgi|Crypa1|102227|estExt_fgenesh1_kg.C_30531 | 29 | 3 | 14.85 | 25127.97 | 4.40 | 0.29 | 0.25 | 0.56 | 0.37 | 0.17 |
| jgi|Crypa1|93945|estExt_Genewise1Plus.C_71306 | 38 | 9 | 16.60 | 55419.63 | 5.43 | 0.28 | 0.43 | 0.38 | 0.36 | 0.08 |
| jgi|Crypa1|81491|estExt_Genewise1.C_41667 | 9 | 2 | 2.03 | 110226.61 | 4.95 | 0.35 | 0.43 | 0.21 | 0.33 | 0.11 |
| jgi|Crypa1|54955|fgenesh1_kg.C_scaffold_2000597 | 6 | 1 | 9.89 | 10327.75 | 5.30 | 0.29 | 0.11 | 0.52 | 0.31 | 0.21 |

Supplementary Table S-3 Identified secreted proteins with no significant change upon hypovirus infection by HPLC-ESI-OrbiTrap MSMS.

a Accession number from Cryphonectria parasitica database v1.0.

b Hypovirus-free strain EP155 was set as reference. The average change (EP713/EP155) were from three independent experimental data. #N/A means the changing tendencies in three independent experiments were not entirely consistent and the protein was considered no significant change.

c The total number of unique matched peptides to the identified protein.

d The cover percentage of matched peptides to the protein sequence.

e Theoretical molecular weight.

f Theoretical pI.

| **No.** | **Accession No.^a^** | **Protein Name** | **Average change**  **(EP713/EP155)^b^** | **Standard**  **error** | **Unique PepCount^c^** | **Cover Percent^d^** | **MW^e^** | **pI^f^** |
| --- | --- | --- | --- | --- | --- | --- | --- | --- |
| 1 | 79019 | HSP70-1 | 1.0 | 0.02 | 27 | 42.44% | 70548.46 | 5.04 |
| 2 | 88329 | Methionine synthase | 0.9 | 0.06 | 27 | 35.72% | 85646.78 | 6.28 |
| 3 | 94119 | Phosphoglycerate mutase, 2,3-bisphosphoglycerate-independent | 1.2 | 0.02 | 26 | 45.85% | 57977.75 | 5.47 |
| 4 | 101915 | Phosphoglycerate kinase | 0.8 | 0.05 | 24 | 55.74% | 44150.61 | 6.57 |
| 5 | 76127 | Catalase/peroxidase | 1.2 | 0.04 | 22 | 29.66% | 83551.56 | 5.63 |
| 6 | 103155 | Enolase | 0.9 | 0.04 | 19 | 46.92% | 47359.65 | 5.25 |
| 7 | 102434 | Phosphoglucomutase | 1.2 | 0.06 | 19 | 36.53% | 59866.56 | 5.52 |
| 8 | 91280 | Hsp70 chaperone | 1.2 | 0.06 | 19 | 35.06% | 67123.01 | 5.18 |
| 9 | 107213 | UDP-N-acetylglucosamine pyrophosphorylase | 1.3 | 0.06 | 19 | 31.63% | 55667.41 | 5.89 |
| 11 | 61733 | Hsp70-like protein | 0.9 | 0.09 | 18 | 29.21% | 72412.78 | 5.8 |
| 12 | 88770 | Heat shock protein 60 | 1.2 | 0.07 | 17 | 28.03% | 61392.1 | 5.6 |
| 13 | 42419 | Transaldolase | 0.7 | 0.08 | 15 | 44.04% | 35786.94 | 5.29 |
| 14 | 98281 | N-acetylglucosamine-phosphate mutase | 1.2 | 0.08 | 15 | 27.93% | 59954.9 | 6.1 |
| 15 | 89617 | Malate dehydrogenase | 0.9 | 0.02 | 14 | 44.21% | 35208.47 | 8.66 |
| 16 | 77086 | Cystathionine beta-synthase | 1.3 | 0.02 | 14 | 25.84% | 57431.37 | 6.31 |
| 17 | 92891 | 6-phosphogluconate dehydrogenase | 0.8 | 0.09 | 14 | 25.67% | 57027.73 | 5.84 |
| 18 | 42922 | Pyruvate kinase | 1.2 | 0.04 | 13 | 25.85% | 58056.21 | 6.54 |
| 19 | 93457 | Glutamate carboxypeptidase | 1.4 | 0.05 | 13 | 32.99% | 52338.42 | 5.22 |
| 20 | 84573 | 78 kDa glucose-regulated protein homolog precursor (GRP 78) | 1.1 | 0.00 | 13 | 17.24% | 73042.35 | 4.8 |
| 21 | 89763 | Glucose-6-phosphate isomerase (GPI) | 1.0 | 0.05 | 13 | 23.76% | 61516.52 | 5.9 |
| 23 | 104856 | D-lactate dehydrogenase | 1.3 | 0.03 | 13 | 27.65% | 48549.36 | 5.97 |
| 25 | 77211 | Cell division control protein Cdc48 | 1.2 | 0.04 | 13 | 17.05% | 89913.29 | 4.95 |
| 27 | 79606 | Pyruvate decarboxylase (8-10 nm cytoplasmic filament-associated protein) | 0.7 | 0.03 | 12 | 21.19% | 62124.84 | 5.55 |
| 28 | 96560 | 3-isopropylmalate dehydrogenase (Beta-IPM dehydrogenase) (IMDH) (3-IPM-DH) | 1.1 | 0.02 | 12 | 29.46% | 39119.83 | 5.31 |
| 29 | 82725 | Aminopeptidase 2 | 0.7 | 0.02 | 12 | 14.82% | 98211.9 | 5.33 |
| 30 | 64909 | Polyadenylate-binding protein, cytoplasmic and nuclear (Poly(A)-binding protein) (PABP) (Polyadenylate tail-binding protein) | 0.7 | 0.05 | 12 | 15.11% | 79755.21 | 5.65 |
| 31 | 83897 | Transketolase | 1.0 | 0.04 | 12 | 16.06% | 74766.49 | 5.74 |
| 33 | 97558 | Alcohol dehydrogenase | 1.4 | 0.07 | 11 | 33.24% | 37915.61 | 6.22 |
| 34 | 80289 | uncharacterized protein | 0.7 | 0.11 | 11 | 36.84% | 36362.44 | 5.63 |
| 35 | 104024 | Actin cytoskeleton protein (VIP1) | 0.8 | 0.04 | 11 | 36.33% | 30790.84 | 5.67 |
| 36 | 94802 | F-actin-capping protein subunit alpha | 1.0 | 0.01 | 11 | 45.26% | 29891.22 | 5.81 |
| 37 | 99283 | Outer mitochondrial membrane protein porin | 1.0 | 0.05 | 11 | 38.52% | 30225.05 | 9.3 |
| 38 | 80153 | Rab GDP-dissociation inhibitor | 1.3 | 0.02 | 11 | 25.65% | 51068.18 | 5.59 |
| 39 | 108086 | uncharacterized protein | 1.0 | 0.03 | 11 | 14.11% | 87585.15 | 5.9 |
| 40 | 80500 | Heat shock protein Hsp88 | 0.8 | 0.04 | 11 | 15.84% | 79987.84 | 5.09 |
| 42 | 42151 | Methyltransferase | 1.2 | 0.02 | 11 | 35.94% | 35755.09 | 5.87 |
| 43 | 102916 | 14-3-3-like protein | 0.8 | 0.09 | 11 | 38.01% | 29977.65 | 4.79 |
| 44 | 99714 | Aconitate hydratase | 1.0 | 0.12 | 11 | 12.56% | 84730.07 | 6.35 |
| 45 | 82471 | Mitochondrial peroxiredoxin PRX1 | 1.1 | 0.04 | 10 | 51.30% | 25567.04 | 5.86 |
| 47 | 23873 | Similar to Osmotic growth protein 1 | 0.9 | 0.03 | 10 | 21.36% | 51043.36 | 6.12 |
| 48 | 85234 | Sec14 cytosolic factor | 0.9 | 0.10 | 10 | 26.63% | 39090.43 | 5.44 |
| 49 | 99702 | Aminopeptidase Y | 0.9 | 0.03 | 10 | 19.96% | 55153.77 | 4.94 |
| 50 | 95090 | Homoserine acetyltransferase | 1.2 | 0.04 | 10 | 26.05% | 39207.48 | 4.97 |
| 51 | 88910 | uncharacterized protein | 0.8 | 0.06 | 10 | 22.54% | 55533.21 | 6.4 |
| 52 | 87995 | heat shock protein 80(Suppressor of vegetative incompatibility MOD-E) | 0.8 | 0.06 | 10 | 15.84% | 79907.41 | 4.89 |
| 53 | 81065 | Dipeptidyl peptidase | 1.3 | 0.02 | 10 | 13.82% | 78001.3 | 5.42 |
| 57 | 47560 | 41 kDa peptidyl-prolyl cis-trans isomerase (PPIase) (Rotamase) (Cyclophilin-41) (CYP-41) | 1.3 | 0.07 | 9 | 24.17% | 42524.03 | 5.73 |
| 58 | 92811 | ATP-dependent RNA helicase eIF4A (Eukaryotic initiation factor 4A) (eIF-4A) (Translation initiation factor 1) | 1.3 | 0.11 | 9 | 23.37% | 44823.45 | 4.97 |
| 59 | 102261 | uncharacterized protein | 1.0 | 0.06 | 9 | 30.75% | 34859.64 | 6.1 |
| 60 | 100550 | Malate dehydrogenase | 1.1 | 0.07 | 9 | 29.85% | 34731.22 | 6.23 |
| 62 | 103985 | 3',5'-bisphosphate nucleotidase | 1.1 | 0.09 | 9 | 30.42% | 37348.32 | 5.19 |
| 63 | 35666 | uncharacterized protein | 1.2 | 0.44 | 9 | 13.94% | 86199.58 | 5.54 |
| 64 | 68536 | Peroxiredoxin (AsPrx) (Thioredoxin peroxidase) | 1.0 | 0.05 | 8 | 29.21% | 22319.47 | 5.3 |
| 65 | 102864 | Elongation factor 1-alpha (EF-1-alpha) | 1.1 | 0.02 | 8 | 15.70% | 50489.1 | 9.15 |
| 67 | 88045 | Actin-3(regulated by circadian rhythms/ vacuole inheritance) | 1.0 | 0.06 | 8 | 25.27% | 41593.54 | 5.45 |
| 68 | 91746 | Alcohol dehydrogenase | 1.0 | 0.09 | 8 | 26.18% | 35478.71 | 7.21 |
| 69 | 94542 | Amidohydrolase | 1.0 | 0.06 | 8 | 17.42% | 57241.07 | 5.95 |
| 70 | 66481 | uncharacterized protein | 1.3 | 0.09 | 8 | 52.86% | 15131.06 | 9.88 |
| 71 | 52735 | Aryl-alcohol oxidase | 1.2 | 0.11 | 8 | 14.58% | 67196.39 | 5.6 |
| 72 | 82249 | uncharacterized protein | 0.9 | 0.08 | 8 | 17.05% | 40761.65 | 7 |
| 73 | 80991 | uncharacterized protein | 0.9 | 0.04 | 8 | 11.04% | 82837.58 | 4.74 |
| 74 | 73267 | Related to stress protein p66 | 1.1 | 0.16 | 8 | 12.89% | 63324.17 | 6.76 |
| 76 | 78037 | Nucleoside diphosphate kinase-1 (NDK-1) | 1.0 | 0.04 | 7 | 50.32% | 16963.47 | 6.83 |
| 77 | 49385 | Aldose 1-epimerase | 1.1 | 0.00 | 7 | 26.38% | 36471.59 | 5.16 |
| 78 | 77119 | Triosephosphate isomerase (TPI) | 1.0 | 0.05 | 7 | 28.00% | 27081.83 | 5.43 |
| 79 | 98830 | Inorganic pyrophosphatase (Pyrophosphate phospho-hydrolase) (PPase) | 1.0 | 0.04 | 7 | 27.34% | 32549.04 | 5.39 |
| 80 | 105107 | uncharacterized protein | 0.8 | 0.13 | 7 | 5.28% | 132237.16 | 3.87 |
| 81 | 76097 | Adenosylhomocysteinase | 0.7 | 0.05 | 7 | 14.89% | 49085.47 | 5.72 |
| 82 | 55273 | Ribose 5-phosphate isomerase-like protein | 0.9 | 0.17 | 7 | 27.50% | 29418.59 | 6.53 |
| 83 | 109525 | FK506 resistant-2(FKR-2)(Peptidyl-prolyl cis-trans isomerase) (PPIase) (Rotamase) (FKBP-21) (NcFKBP22) | 1.2 | 0.08 | 7 | 28.80% | 20367.19 | 4.82 |
| 84 | 85769 | S-adenosylmethionine synthetase (Methionine adenosyltransferase) (AdoMet synthetase) | 0.7 | 0.18 | 7 | 16.79% | 43326.97 | 5.84 |
| 85 | 88762 | ATP synthase subunit beta, mitochondrial precursor | 1.1 | 0.08 | 7 | 17.15% | 55596.39 | 5.28 |
| 86 | 105502 | 60S ribosomal protein L4 | 1.0 | 0.15 | 7 | 22.31% | 39903.22 | 10.5 |
| 87 | 76626 | uncharacterized protein | 1.2 | 0.10 | 7 | 18.72% | 39513.61 | 6.28 |
| 88 | 85703 | Clathrin light chain (regulated by circadian rhythms) | 1.1 | 0.09 | 7 | 23.61% | 25100.05 | 4.46 |
| 89 | 63094 | Aldo/keto reductase | 1.4 | 0.03 | 7 | 15.06% | 36946.22 | 5.97 |
| 90 | 52030 | D-3-phosphoglycerate dehydrogenase | 0.7 | 0.15 | 7 | 14.90% | 50416.83 | 5.98 |
| 91 | 69531 | Glycogen phosphorylase | #N/A | #N/A | 7 | 7.57% | 101592.68 | 5.63 |
| 92 | 63427 | Proliferating cell nuclear antigen | 1.0 | 0.17 | 7 | 21.15% | 28492.45 | 4.5 |
| 93 | 53221 | uncharacterized protein | 1.0 | 0.04 | 6 | 14.46% | 54888.2 | 4.15 |
| 94 | 107233 | uncharacterized protein | #N/A | #N/A | 6 | 14.90% | 47042.85 | 4.84 |
| 95 | 43719 | Peroxiredoxin | 0.8 | 0.07 | 6 | 45.78% | 17817.31 | 6.13 |
| 96 | 101944 | uncharacterized protein | 1.0 | 0.04 | 6 | 52.83% | 11648.22 | 5.54 |
| 97 | 102797 | Subtilisin-like proteinase | 1.0 | 0.03 | 6 | 9.56% | 55266.38 | 5.31 |
| 98 | 102031 | uncharacterized protein | 0.9 | 0.01 | 6 | 25.00% | 31641.72 | 5.19 |
| 102 | 78256 | ATP-citrate synthase subunit 1 (ATP-citrate (pro-S-)-lyase 1) (Citrate cleavage enzyme subunit 1) | 0.9 | 0.10 | 6 | 9.68% | 71656.69 | 8.65 |
| 103 | 107307 | Acetyl-CoA acetyltransferase | 1.2 | 0.06 | 6 | 12.97% | 41234.26 | 6.67 |
| 104 | 84488 | Hydroxymethylglutaryl-CoA synthase | 0.9 | 0.07 | 6 | 11.04% | 50654.45 | 6.47 |
| 105 | 103071 | NAD(P)H-dependent D-xylose reductase (XR) | 1.3 | 0.05 | 6 | 20.12% | 36176.34 | 5.4 |
| 106 | 77940 | NADPH:quinone reductase and related Zn-dependent oxidoreductases | 0.8 | 0.07 | 6 | 17.23% | 36473.58 | 6.54 |
| 107 | 95810 | Elongation factor 1-gamma | 1.2 | 0.10 | 6 | 14.46% | 46658.73 | 6.08 |
| 108 | 64397 | uncharacterized protein | 1.0 | 0.06 | 6 | 9.88% | 63476.95 | 4.45 |
| 111 | 100991 | Protein disulfide-isomerase erp38 precursor (ERp38) | 1.1 | 0.05 | 6 | 16.49% | 39862.3 | 5.78 |
| 112 | 68371 | Aspartate aminotransferase | 0.7 | 0.13 | 6 | 14.45% | 45842.27 | 8.87 |
| 113 | 99253 | FAD dependent oxidoreductase | 1.3 | 0.04 | 6 | 14.01% | 50366.25 | 5.76 |
| 114 | 34543 | Porphobilinogen deaminase (PBG) (Hydroxymethylbilane synthase) (HMBS) (Pre-uroporphyrinogen synthase) | 1.4 | 0.05 | 6 | 14.88% | 38065.7 | 5.5 |
| 115 | 102565 | 40S ribosomal protein S11 | 1.3 | 0.08 | 6 | 35.00% | 18155.25 | 10.93 |
| 116 | 82721 | Valyl-tRNA synthetase, mitochondrial precursor (Valine--tRNA ligase) (ValRS) | 1.1 | 0.13 | 6 | 5.81% | 122787.49 | 5.9 |
| 117 | 102090 | uncharacterized protein | 0.8 | 0.04 | 5 | 58.75% | 8299.14 | 4.58 |
| 120 | 83961 | Vacuolar protease A precursor (PEP-4) | 1.0 | 0.04 | 5 | 16.28% | 42581.99 | 4.81 |
| 122 | 92473 | 60S acidic ribosomal protein P2 | 1.4 | 0.06 | 5 | 61.61% | 11365.51 | 4.32 |
| 123 | 89863 | GMC oxidoreductase | 0.8 | 0.05 | 5 | 9.42% | 67689.24 | 4.16 |
| 124 | 36435 | Aspartyl aminopeptidase | 1.4 | 0.08 | 5 | 11.13% | 54779.04 | 5.6 |
| 125 | 77315 | Translationally-controlled tumor protein homolog (TCTP) | 1.4 | 0.02 | 5 | 26.90% | 19104.61 | 4.49 |
| 127 | 81476 | Aldose-1-epimerase | 0.9 | 0.04 | 5 | 21.98% | 34448.5 | 4.9 |
| 129 | 94341 | L-xylulose reductase (XR) | 1.3 | 0.06 | 5 | 14.61% | 28188.84 | 5.65 |
| 130 | 104409 | Histone H2B | 1.2 | 0.09 | 5 | 30.94% | 14932.08 | 10.12 |
| 132 | 74392 | Tubulin beta chain (Beta-tubulin) | 0.7 | 0.1 | 5 | 9.60% | 49888.17 | 4.75 |
| 134 | 35264 | Adenosine kinase | 0.9 | 0.17 | 5 | 15.43% | 37969.23 | 5.43 |
| 135 | 89596 | UDP-glucose 4-epimerase | 1.0 | 0.05 | 5 | 13.71% | 40810.34 | 5.78 |
| 136 | 86878 | cytoplasmic ribosomal protein-(CRP-10)Cytoplasmic ribosomal protein subunit S3 | 1.3 | 0.09 | 5 | 20.99% | 28506.67 | 9.2 |
| 137 | 35302 | Dipeptidase | 1.0 | 0.05 | 5 | 12.44% | 45873.61 | 5.03 |
| 138 | 88593 | Tubulin specific chaperone cofactor B | 1.1 | 0.12 | 5 | 19.42% | 26537.96 | 4.87 |
| 139 | 107358 | uncharacterized protein | 0.8 | 0.10 | 5 | 11.61% | 55966.38 | 5.13 |
| 140 | 73563 | Dienelactone hydrolase | 1.2 | 0.05 | 5 | 13.68% | 35041.19 | 4.6 |
| 141 | 34049 | Proteasome subunit alpha type-1 | 0.9 | 0.11 | 5 | 15.30% | 30406.14 | 4.93 |
| 143 | 62138 | Sedoheptulose-1,7-bisphosphatase | 0.9 | 0.10 | 5 | 15.79% | 34329.21 | 5.7 |
| 144 | 92971 | Hexokinase | 0.9 | 0.12 | 5 | 8.10% | 54208.08 | 5.17 |
| 145 | 103805 | uncharacterized protein | 1.1 | 0.24 | 5 | 22.13% | 37507.9 | 4.79 |
| 146 | 40976 | Dihydrolipoyllysine-residue acetyltransferase component of pyruvate dehydrogenase complex, mitochondrial precursor (E2) (Dihydrolipoamide acetyltransferase component of pyruvate dehydrogenase complex) (PDC-E2) (MRP3) | 0.8 | 0.10 | 5 | 10.58% | 49074.93 | 5.71 |
| 147 | 63707 | 1,3-beta-glucanosyltransferase gel1 precursor (Glucan elongating glucanosyltransferase 1) | 0.9 | 0.07 | 5 | 13.33% | 46397.85 | 4.01 |
| 149 | 100169 | Saccharopine dehydrogenase [NADP+, L-glutamate-forming] (Saccharopine reductase) | 0.7 | 0.18 | 5 | 9.56% | 48931.26 | 6.19 |
| 150 | 92044 | uncharacterized protein | 1.2 | 0.07 | 5 | 16.28% | 28469.42 | 5.83 |
| 153 | 63766 | Carbon-nitrogen hydrolase | 1.0 | 0.04 | 5 | 16.12% | 33353.26 | 6.09 |
| 155 | 93017 | Saccharopine dehydrogenase [NAD+, L-lysine-forming] (Lysine--2-oxoglutarate reductase) (SDH) | 0.9 | 0.19 | 5 | 14.36% | 41482.31 | 5.52 |
| 156 | 106622 | Sulfite reductase | #N/A | #N/A | 5 | 3.33% | 172734.36 | 5.79 |
| 157 | 63408 | Argininosuccinate synthase | 0.9 | 0.09 | 5 | 9.09% | 46688.35 | 5.19 |
| 158 | 72159 | Thioredoxin reductase | 1.3 | 0.11 | 5 | 15.73% | 35988.39 | 5.26 |
| 159 | 86840 | Ornithine aminotransferase (Ornithine--oxo-acid aminotransferase) | 1.1 | 0.37 | 5 | 11.69% | 47807.79 | 6.26 |
| 164 | 101182 | uncharacterized protein | 1.0 | 0.05 | 4 | 31.71% | 13532.83 | 5.07 |
| 166 | 102097 | FK506-binding protein 1B (FKBP) (Peptidyl-prolyl cis-trans isomerase) (PPIase) (Rapamycin-binding protein) | 0.7 | 0.1 | 4 | 31.58% | 11975.33 | 6.82 |
| 168 | 102285 | uncharacterized protein | #N/A | #N/A | 4 | 26.34% | 23321.08 | 4.96 |
| 170 | 101854 | Myosin regulatory light chain cdc4 | 0.9 | 0.16 | 4 | 23.65% | 16420.18 | 4.43 |
| 174 | 63198 | Nuclear movement protein nudC | #N/A | #N/A | 4 | 16.32% | 21145.85 | 5.17 |
| 175 | 99754 | Homoserine dehydrogenase | 0.8 | 0.06 | 4 | 10.60% | 38826.25 | 6.04 |
| 176 | 103641 | NAD(P)-binding protein | 1.2 | 0.07 | 4 | 11.85% | 36598.73 | 6.09 |
| 177 | 85077 | uncharacterized protein | 1.1 | 0.06 | 4 | 24.56% | 18945.62 | 8.54 |
| 178 | 47978 | uncharacterized protein | 1.1 | 0.03 | 4 | 22.28% | 20332.03 | 4.81 |
| 179 | 106534 | uncharacterized protein | 0.9 | 0.09 | 4 | 25.85% | 22719.86 | 5.98 |
| 180 | 50968 | NADH:flavin oxidoreductase/NADH oxidase family protein | 0.8 | 0.10 | 4 | 8.67% | 43754.48 | 5.61 |
| 181 | 38338 | Alanine-glyoxylate aminotransferase | 1.1 | 0.03 | 4 | 9.84% | 41729.87 | 6.02 |
| 183 | 100587 | uncharacterized protein | 0.7 | 0.13 | 4 | 12.82% | 32722.31 | 9.27 |
| 184 | 80744 | Aspartyl-tRNA synthetase | 0.8 | 0.20 | 4 | 8.70% | 51191.66 | 5.37 |
| 185 | 101783 | uncharacterized protein | 0.9 | 0.08 | 4 | 37.17% | 12248.87 | 5.58 |
| 186 | 71911 | UMP-CMP kinase | 1.1 | 0.18 | 4 | 20.40% | 22118.3 | 5.47 |
| 187 | 88317 | ATP citrate lyase | 1.4 | 0.03 | 4 | 7.36% | 53002.53 | 5.71 |
| 189 | 99673 | Arginase | 1.2 | 0.48 | 4 | 10.00% | 37552.33 | 5.58 |
| 190 | 107162 | Histidine biosynthesis trifunctional protein [Includes: Phosphoribosyl-AMP cyclohydrolase ; Phosphoribosyl-ATP pyrophosphohydrolase ; Histidinol dehydrogenase (HDH)] | 1.2 | 0.08 | 4 | 5.18% | 93848.29 | 5.93 |
| 192 | 25948 | UV excision repair protein Rad23 | 1.0 | 0.18 | 4 | 9.44% | 40728.44 | 4.52 |
| 193 | 43908 | GTP-binding nuclear protein GSP1/Ran | 0.7 | 0.09 | 4 | 15.67% | 24459.81 | 6.11 |
| 194 | 89229 | uncharacterized protein | 1.0 | 0.17 | 4 | 10.48% | 39453.36 | 5.88 |
| 195 | 49897 | Peptidase family M20/M25/M40 | 0.9 | 0.05 | 4 | 6.53% | 65498.26 | 5.18 |
| 196 | 86899 | uncharacterized protein | 1.2 | 0.17 | 4 | 6.96% | 50966.28 | 6.46 |
| 197 | 43297 | 60S ribosomal protein L6 | 1.3 | 0.05 | 4 | 18.50% | 22470.06 | 10.12 |
| 200 | 57041 | MYG1 protein | 1.1 | 0.22 | 4 | 10.56% | 39678.56 | 5.48 |
| 201 | 66428 | Ribonucleoside-diphosphate reductase small chain (Ribonucleotide reductase small subunit) | 0.7 | 0.16 | 4 | 8.35% | 47645.2 | 5.03 |
| 203 | 43898 | uncharacterized protein | 1.4 | 0.64 | 4 | 13.32% | 41038.67 | 5.9 |
| 204 | 87233 | 60S ribosomal protein L2 | 1.1 | 0.09 | 4 | 15.69% | 27571.65 | 10.96 |
| 205 | 88774 | Cofilin (Actin-depolymerizing factor 1) | 1.2 | 0.04 | 4 | 26.62% | 16927.94 | 5.46 |
| 206 | 93998 | uncharacterized protein | 1.2 | 0.06 | 4 | 7.35% | 59082.02 | 4.74 |
| 209 | 98458 | Inosine-5'-monophosphate dehydrogenase | 0.9 | 0.21 | 4 | 7.75% | 57337.35 | 6.24 |
| 210 | 70070 | Fatty acid synthase subunit alpha [Includes: Acyl carrier; 3-oxoacyl-[acyl-carrier-protein] reductase (Beta-ketoacyl reductase); 3-oxoacyl-[acyl-carrier-protein] synthase (Beta-ketoacyl synthase)] | #N/A | #N/A | 4 | 1.85% | 202261.3 | 5.77 |
| 211 | 38677 | Diphosphomevalonate decarboxylase | 0.8 | 0.20 | 4 | 6.38% | 89234.45 | 5.59 |
| 212 | 59332 | uncharacterized protein | 1.1 | 0.08 | 4 | 16.60% | 28597.04 | 5.16 |
| 213 | 107004 | Chaperone protein dnaK | #N/A | #N/A | 4 | 5.80% | 64298.86 | 4.84 |
| 214 | 38710 | uncharacterized protein | 1.1 | 0.18 | 4 | 25.17% | 17220.1 | 4.8 |
| 215 | 106261 | uncharacterized protein | 1.2 | 0.32 | 4 | 8.35% | 49436.88 | 5.12 |
| 216 | 83235 | 60S ribosomal protein L5 (CPR4) | 1.1 | 0.33 | 4 | 11.30% | 34626.84 | 7.08 |
| 218 | 51266 | uncharacterized protein | #N/A | #N/A | 4 | 27.01% | 15100.54 | 6.65 |
| 219 | 69622 | Short chain dehydrogenase | 1.2 | 0.17 | 4 | 13.87% | 29265.67 | 6.65 |
| 220 | 94953 | 60S ribosomal protein L13 | 0.9 | 0.22 | 4 | 16.20% | 24240.08 | 10.78 |
| 222 | 107789 | Alanyl-tRNA synthetase | 0.9 | 0.18 | 4 | 4.26% | 108168.9 | 5.49 |
| 223 | 101333 | vacuolar membrane ATPase-2(VMA-2)Vacuolar ATP synthase subunit B (V-ATPase subunit B) (Vacuolar proton pump subunit B) (V-ATPase 57 kDa subunit) | 0.8 | 0.36 | 4 | 5.70% | 56255.91 | 5.44 |
| 224 | 76452 | uncharacterized protein | 1.5 | 0.35 | 4 | 5.85% | 66858.09 | 5.85 |
| 225 | 80484 | Eukaryotic translation initiation factor 3 subunit G (eIF3g) | 0.9 | 0.21 | 4 | 10.44% | 32313.18 | 8.24 |
| 228 | 104580 | uncharacterized protein | 1.0 | 0.36 | 4 | 16.77% | 35763.17 | 8.76 |
| 229 | 85799 | UTH1 | 0.9 | 0.02 | 3 | 6.64% | 44922.49 | 4.38 |
| 231 | 100735 | 22kDa glycoprotein | 1.2 | 0.02 | 3 | 30.50% | 14167.49 | 4.36 |
| 235 | 39961 | Thioredoxin | 0.9 | 0.04 | 3 | 27.12% | 13032.88 | 5.36 |
| 237 | 102068 | Eukaryotic translation initiation factor 5A-2 (eIF-5A-2) (eIF-4D) (Hypusine-containing protein HP2) | 1.0 | 0.1 | 3 | 12.73% | 17836 | 4.93 |
| 239 | 100503 | 40S ribosomal protein S7 | 1.3 | 0.09 | 3 | 11.27% | 22940.34 | 10.06 |
| 240 | 102665 | Rossmann-fold NAD(P)(+)-binding protein | 1.4 | 0.11 | 3 | 11.15% | 27751.48 | 5.45 |
| 242 | 65706 | Lactoylglutathione lyase | 0.8 | 0.05 | 3 | 7.48% | 36321.64 | 5.69 |
| 245 | 36895 | uncharacterized protein | 1.2 | 0.15 | 3 | 22.22% | 12568.24 | 5.12 |
| 246 | 100631 | Extracellular carboxypeptidase | 1.1 | 0.11 | 3 | 5.08% | 61053.42 | 4.75 |
| 247 | 100903 | Epoxide hydrolase | 0.9 | 0.08 | 3 | 8.50% | 37634.22 | 5.64 |
| 248 | 106144 | Cytochrome c | 0.9 | 0.11 | 3 | 26.61% | 12002.58 | 9.34 |
| 249 | 59925 | dihydroxy-acid dehydratase | 0.7 | 0.35 | 3 | 5.17% | 63901.75 | 6.19 |
| 250 | 96955 | Glutathione peroxidase | 0.9 | 0.07 | 3 | 19.30% | 19031.82 | 6.62 |
| 251 | 31781 | Aado/keto reductase | 1.4 | 0.06 | 3 | 11.41% | 28520.51 | 6.27 |
| 252 | 102482 | 60S ribosomal protein L31 | #N/A | #N/A | 3 | 21.95% | 14010.15 | 10.25 |
| 253 | 102577 | NAD dependent epimerase/dehydratase | 1.4 | 0.04 | 3 | 11.79% | 28513.55 | 6.37 |
| 255 | 108592 | uncharacterized protein | 1.0 | 0.06 | 3 | 13.30% | 25044.54 | 4.61 |
| 256 | 102630 | 40S ribosomal protein S21 (CRP7) | 1.3 | 0.17 | 3 | 25.00% | 9633.83 | 7.81 |
| 257 | 93941 | NAD(P)-binding protein | 1.4 | 0.10 | 3 | 9.45% | 28331.34 | 8.33 |
| 258 | 81789 | Eukaryotic translation initiation factor 1A (EIF-1A) (EIF-4C) | 1.0 | 0.14 | 3 | 20.89% | 17595.4 | 4.88 |
| 259 | 40873 | 3-isopropylmalate dehydratase | 1.1 | 0.18 | 3 | 3.98% | 84338.01 | 5.94 |
| 260 | 88636 | proteasome subunit alpha type-2 | 1.3 | 0.11 | 3 | 10.45% | 30688.06 | 6.04 |
| 262 | 100994 | uncharacterized protein | 0.9 | 0.12 | 3 | 12.45% | 27345.34 | 4.97 |
| 265 | 108594 | uncharacterized protein | 0.9 | 0.11 | 3 | 8.09% | 35367.69 | 5.99 |
| 266 | 99715 | Threonyl-tRNA synthetase-like protein | 1.0 | 0.25 | 3 | 3.98% | 88700.5 | 6.57 |
| 268 | 102078 | Phosphatidylglycerol/phosphatidylinositol transfer protein precursor (PG/PI-TP) | 1.0 | 0.15 | 3 | 14.77% | 18762.73 | 4.63 |
| 269 | 86554 | Xaa-Pro aminopeptidase | 1.0 | 0.07 | 3 | 5.65% | 51103.8 | 5.37 |
| 270 | 87599 | FAD binding domain containing protein | 1.4 | 0.32 | 3 | 8.38% | 56233.84 | 4.49 |
| 271 | 59013 | S-formylglutathione hydrolase | 0.8 | 0.22 | 3 | 9.63% | 29644.4 | 5.46 |
| 273 | 63966 | Isocitrate dehydrogenase [NADP] | 0.9 | 0.19 | 3 | 7.52% | 46314.07 | 6.24 |
| 274 | 108424 | uncharacterized protein | 1.0 | 0.04 | 3 | 2.90% | 112449 | 8.71 |
| 275 | 43065 | Cyclopentanol dehydrogenase | 1.0 | 0.21 | 3 | 12.68% | 29794.01 | 5.98 |
| 276 | 96894 | Nitroreductase | 1.2 | 0.16 | 3 | 18.04% | 21573.52 | 5.38 |
| 277 | 80224 | Ubiquitin-activating enzyme E1 1 | 1.1 | 0.15 | 3 | 3.03% | 112861.1 | 5 |
| 278 | 69628 | uncharacterized protein | 1.1 | 0.19 | 3 | 10.95% | 21823.89 | 6.61 |
| 282 | 91265 | high mobility group protein | 1.1 | 0.22 | 3 | 7.46% | 36094.1 | 9.11 |
| 284 | 79471 | Elongation factor 3 | 1.2 | 0.19 | 3 | 2.55% | 117223.46 | 6.07 |
| 287 | 107659 | Calnexin homolog precursor | 1.2 | 0.18 | 3 | 3.65% | 63513.21 | 4.69 |
| 288 | 105574 | 3-hydroxyacyl-CoA dehydrogenase | 1.1 | 0.12 | 3 | 9.81% | 33457.72 | 8.2 |
| 292 | 95427 | Alanine racemase TOXG | #N/A | #N/A | 3 | 5.97% | 41743.67 | 6.2 |
| 294 | 65476 | seryl-tRNA synthetase | 1.0 | 0.16 | 3 | 5.96% | 53286.26 | 5.97 |
| 300 | 100383 | Endothiapepsin precursor (Aspartate protease) | 1.3 | 0.03 | 2 | 6.19% | 43254.91 | 4.77 |
| 306 | 57583 | Nuclear transport factor 2 (NTF-2) | 0.9 | 0.04 | 2 | 12.60% | 14055.95 | 4.98 |
| 307 | 55727 | Protein HMF1 | 0.9 | 0.04 | 2 | 21.14% | 13031.07 | 5.41 |
| 309 | 94005 | Endothiapepsin precursor (Aspartate protease) | 0.7 | 0.14 | 2 | 6.53% | 36585.67 | 3.84 |
| 310 | 64622 | Tripeptidyl peptidase | #N/A | #N/A | 2 | 5.86% | 65075.12 | 4.43 |
| 314 | 45332 | O-methyltransferase | 1.0 | 0.09 | 2 | 8.60% | 23711.2 | 5.27 |
| 316 | 55968 | 60S ribosomal protein L23 | 1.0 | 0.08 | 2 | 16.43% | 14638.25 | 10.33 |
| 317 | 66294 | Galactoside O-acetyltransferase | 0.7 | 0.08 | 2 | 19.44% | 12134.83 | 5.57 |
| 318 | 94557 | BRT1 | 1.2 | 0.12 | 2 | 16.92% | 14124.02 | 5.08 |
| 319 | 102428 | uncharacterized protein | 1.4 | 0.05 | 2 | 8.98% | 27527.14 | 4.59 |
| 320 | 69130 | Cell wall protein AWA1 precursor | 1.0 | 0.07 | 2 | 2.41% | 74909.97 | 3.96 |
| 321 | 79008 | Perilipin MPL1-like protein | 1.2 | 0.03 | 2 | 10.87% | 19993.71 | 8.83 |
| 324 | 93442 | Methionine aminopeptidase | 1.2 | 0.16 | 2 | 5.31% | 48934.82 | 5.66 |
| 326 | 103050 | 40S ribosomal protein S2-like protein | 1.2 | 0.23 | 2 | 6.92% | 28351.86 | 10.4 |
| 328 | 106307 | Rnapii degradation factor | 0.9 | 0.05 | 2 | 2.77% | 104071.98 | 5.76 |
| 329 | 89436 | GPI-anchored cell wall beta-1,3-endoglucanase | #N/A | #N/A | 2 | 6.67% | 41017.25 | 4.03 |
| 330 | 101666 | 40S ribosomal protein S10 | 1.4 | 0.06 | 2 | 12.88% | 18476.96 | 9.73 |
| 332 | 99827 | Probable cytochrome b5 | 1.1 | 0.08 | 2 | 17.61% | 15126.72 | 4.44 |
| 333 | 103302 | 60S ribosomal protein L22 | 1.4 | 0.02 | 2 | 20.97% | 13972.97 | 9.22 |
| 334 | 102157 | Cytochrome b-c1 complex subunit Rieske, mitochondrial precursor (Ubiquinol-cytochrome c reductase iron-sulfur subunit) (Rieske iron-sulfur protein) (RISP) (Complex III subunit 5) | 1.1 | 0.14 | 2 | 7.69% | 25195.84 | 8.56 |
| 335 | 36141 | Thiamin pyrophosphokinase | 1.0 | 0.07 | 2 | 5.59% | 39395.98 | 5.09 |
| 336 | 45775 | Thioredoxin | 1.1 | 0.06 | 2 | 8.38% | 36493.14 | 4.81 |
| 341 | 90806 | Woronin body major protein precursor | 1.3 | 0.16 | 2 | 11.18% | 16837.2 | 6.54 |
| 342 | 72967 | uncharacterized protein | #N/A | #N/A | 2 | 22.22% | 14241.77 | 5.19 |
| 343 | 34225 | Vacuolar aminopeptidase 1 | 1.0 | 0.18 | 2 | 3.65% | 52673.94 | 6.07 |
| 344 | 82588 | 6,7-dimethyl-8-ribityllumazine synthase (DMRL synthase) (Lumazine synthase) (Riboflavin synthase beta chain) | 1.3 | 0.17 | 2 | 10.00% | 20701.42 | 5.84 |
| 347 | 109500 | FAD binding domain-containing protein | 0.7 | 0.05 | 2 | 3.18% | 50682.41 | 4.28 |
| 348 | 37540 | uncharacterized protein | 1.1 | 0.06 | 2 | 1.80% | 99254.69 | 5.22 |
| 351 | 94757 | uncharacterized protein | 1.1 | 0.23 | 2 | 2.37% | 87635.09 | 4.77 |
| 352 | 58417 | Calmodulin (CaM) | 0.8 | 0.08 | 2 | 16.00% | 16996.71 | 4.11 |
| 353 | 105126 | uncharacterized protein | 0.9 | 0.02 | 2 | 4.91% | 55067.83 | 4.83 |
| 354 | 70589 | uncharacterized protein | 1.4 | 0.11 | 2 | 8.72% | 16872.78 | 5.01 |
| 355 | 88903 | 60S ribosomal protein L27 | 0.7 | 0.03 | 2 | 16.18% | 15672.34 | 10.53 |
| 357 | 99093 | Deoxyuridine 5'-triphosphate nucleotidohydrolase (dUTPase) (dUTP pyrophosphatase) | 1.2 | 0.17 | 2 | 11.56% | 21070.92 | 9.09 |
| 358 | 74071 | Prenylcysteine oxidase | 0.8 | 0.14 | 2 | 5.82% | 60722.62 | 4.65 |
| 360 | 104818 | b-1,3-glucanosyltransferase; CBM43 module; GPI-anchor , secreted | 1.4 | 0.07 | 2 | 5.56% | 57472.4 | 4.12 |
| 361 | 72340 | Nuclear localization sequence binding protein | 1.2 | 0.03 | 2 | 3.75% | 53136.73 | 4.75 |
| 362 | 52977 | Exoglucanase 2 precursor (Exoglucanase II) (Exocellobiohydrolase II) (CBHII) (1,4-beta-cellobiohydrolase) | 1.2 | 0.26 | 2 | 5.46% | 42217.74 | 4.46 |
| 365 | 78120 | uncharacterized protein | 1.2 | 0.14 | 2 | 4.07% | 50912.9 | 7.07 |
| 366 | 94251 | Endothelin-converting enzyme | 1.3 | 0.16 | 2 | 2.33% | 86138.9 | 4.91 |
| 367 | 99084 | Serine/threonine protein kinase | 1.1 | 0.37 | 2 | 2.69% | 74236.94 | 5.53 |
| 370 | 107537 | uncharacterized protein | 1.4 | 0.13 | 2 | 2.80% | 68989.39 | 5.11 |
| 371 | 50954 | uncharacterized protein | 0.8 | 0.04 | 2 | 5.32% | 39359.53 | 5.42 |
| 372 | 80978 | Ubiquitin-like protein | 0.8 | 0.24 | 2 | 25.23% | 11885.07 | 4.83 |
| 374 | 55753 | Non-histone chromosomal protein 6 | 1.0 | 0.03 | 2 | 11.58% | 10585.03 | 9.91 |
| 377 | 33751 | Agmatinase | #N/A | #N/A | 2 | 6.89% | 42369.82 | 4.85 |
| 378 | 105464 | Leucine permease transcriptional regulator | 1.0 | 0.10 | 2 | 1.19% | 163605.45 | 8.91 |
| 379 | 64200 | uncharacterized protein | 1.2 | 0.30 | 2 | 3.73% | 50056.83 | 4.99 |
| 380 | 71110 | uncharacterized protein | 1.1 | 0.08 | 2 | 3.81% | 50164.77 | 5.87 |
| 382 | 69552 | Replication factor-A protein | 1.4 | 0.05 | 2 | 7.39% | 30482.38 | 4.76 |
| 384 | 95236 | uncharacterized protein | #N/A | #N/A | 2 | 11.61% | 15812.76 | 6.28 |
| 385 | 84041 | Argonaute-like protein. the catalytic components of the RNA-induced silencing complex (RISC), the protein complex responsible for the gene silencing phenomenon known as RNA interference (RNAi). | #N/A | #N/A | 2 | 1.67% | 99310.95 | 9.34 |
| 386 | 43362 | Peptidyl-prolyl cis-trans isomerase H (PPIase H) (Rotamase H) | 1.3 | 0.32 | 2 | 8.24% | 19979.64 | 6.9 |
| 387 | 90560 | Isocitrate dehydrogenase | 1.0 | 0.13 | 2 | 5.14% | 41619.74 | 6.72 |
| 390 | 100977 | importin beta-4(regulated by circadian rhythms/regulation of mitotic cell cycle) | 0.9 | 0.25 | 2 | 3.27% | 59926.98 | 4.96 |
| 396 | 61858 | Isocitrate lyase | 1.0 | 0.24 | 2 | 4.39% | 62673.81 | 5.97 |
| 397 | 58481 | uncharacterized protein | 1.1 | 0.05 | 2 | 9.09% | 25282.21 | 5.38 |
| 400 | 102464 | ADP-ribosylation factor | 1.2 | 0.11 | 2 | 9.19% | 20917.96 | 6.16 |
| 401 | 34291 | 60S ribosomal protein L23 | 0.9 | 0.26 | 2 | 15.49% | 15692.36 | 10.13 |
| 403 | 80629 | Cytochrome c oxidase polypeptide IV, mitochondrial precursor | #N/A | #N/A | 2 | 10.05% | 20836.56 | 6.45 |
| 405 | 91966 | uncharacterized protein | 1.0 | 0.13 | 2 | 4.57% | 36854.49 | 5.68 |
| 409 | 96214 | Formyltetrahydrofolate deformylase | #N/A | #N/A | 2 | 8.68% | 31786.71 | 6.67 |
| 418 | 55625 | 40S ribosomal protein S4 | 1.3 | 0.15 | 2 | 4.94% | 29460.15 | 10.32 |
| 467 | 103814 | Cell wall protein PhiA | 1.1 | 0.13 | 1 | 7.98% | 19355.57 | 4.13 |
| 468 | 91219 | Aorsin precursor, serine proteinase with trypsin-like specificity at acidic pH | 0.9 | 0.15 | 1 | 3.15% | 68512.16 | 4.6 |
| 469 | 70492 | uncharacterized protein | #N/A | #N/A | 1 | 6.96% | 24430.37 | 4.01 |
| 470 | 103631 | uncharacterized protein | 0.8 | 0.05 | 1 | 3.09% | 29673.31 | 4.57 |
| 471 | 33445 | 2-nitropropane dioxygenase | 1.2 | 0.02 | 1 | 4.23% | 34583.61 | 5.94 |
| 472 | 70476 | uncharacterized protein | 1.2 | 0.36 | 1 | 8.94% | 18731.55 | 3.94 |
| 473 | 93764 | Ribonuclease T2-like precursor (RNase T2-like) | 1.4 | 0.06 | 1 | 2.45% | 47835.95 | 3.77 |
| 475 | 70238 | Catalytic activity: deacetylation of xylans and xylo-oligosaccharides | 0.8 | 0.29 | 1 | 4.44% | 26696.79 | 4.53 |
| 476 | 49665 | 2,3-dihydroxybenzoic acid decarboxylase (DHBD) (o-pyrocatechuate decarboxylase) (2,3-DHBA decarboxylase) | 0.8 | 0.30 | 1 | 2.82% | 40279.95 | 5.76 |
| 479 | 63085 | Eukaryotic translation initiation factor 3 subunit D (eIF3d) | #N/A | #N/A | 1 | 1.55% | 63901.73 | 5.21 |
| 481 | 47193 | 5-hydroxyisourate hydrolase | 1.4 | 0.05 | 1 | 7.74% | 16844.89 | 5.44 |
| 483 | 83378 | uncharacterized protein | 1.1 | 0.11 | 1 | 7.20% | 28500.48 | 5.42 |
| 484 | 104400 | Histone H2A | 0.8 | 0.34 | 1 | 6.72% | 14070.19 | 10.55 |
| 485 | 84160 | Serine-type peptidase | 1.3 | 0.19 | 1 | 1.82% | 60747.31 | 4.3 |
| 486 | 69051 | uncharacterized protein | 0.8 | 0.25 | 1 | 1.17% | 158980.27 | 6.87 |
| 488 | 53829 | 60S ribosomal protein L34 | 1.0 | 0.1 | 1 | 6.84% | 13194.37 | 10.77 |
| 490 | 52243 | AP-1 complex subunit gamma-1 | #N/A | #N/A | 1 | 1.30% | 92127.1 | 6.27 |
| 494 | 105958 | EF hand domain-containing protein | 1.2 | 0.09 | 1 | 0.79% | 131076.68 | 4.65 |
| 495 | 73146 | Ubiquitin C-terminal hydrolase | 1.0 | 0.19 | 1 | 1.28% | 101794.33 | 6.66 |
| 497 | 102177 | uncharacterized protein | #N/A | #N/A | 1 | 3.08% | 35398.26 | 5.49 |
| 498 | 54042 | 40S ribosomal protein S29 | 1.0 | 0.03 | 1 | 14.04% | 6498.55 | 10.39 |
| 499 | 77821 | Glycine cleavage system H protein-like protein | 0.8 | 0.22 | 1 | 5.14% | 18578.98 | 4.58 |
| 510 | 84265 | Cyanide hydratase (Formamide hydrolyase) | 1.4 | 0.43 | 1 | 2.65% | 41900.38 | 5.69 |
| 514 | 79545 | uncharacterized protein | 1.0 | 0.19 | 1 | 3.27% | 37107.25 | 4.89 |

Supplementary Table S-4 The list of identified proteins which did not show in all three independent experiments by HPLC-ESI-OrbiTrap MSMS.

a Accession number from Cryphonectria parasitica database v1.0.

c The total number of unique matched peptides to the identified protein.

d The cover percentage of matched peptides to the protein sequence.

e Theoretical molecular weight.

f Theoretical pI.

| **No.** | **Accession No.^a^** | **Protein Name** | **Unique PepCount^c^** | **Cover Percent^d^** | **MW^e^** | **pI^f^** |
| --- | --- | --- | --- | --- | --- | --- |
| 208 | 103109 | 60S ribosomal protein L33-A | 4 | 30.00% | 12195.08 | 10.7 |
| 217 | 109325 | uncharacterized protein | 4 | 16.67% | 28794.31 | 9.33 |
| 221 | 79548 | galactokinase | 4 | 6.92% | 57068.39 | 5.4 |
| 227 | 96877 | metallopeptidase | 4 | 8.48% | 43981.44 | 8.07 |
| 264 | 98901 | delta-1-pyrroline-5-carboxylate dehydrogenase | 3 | 5.19% | 62556.95 | 8.37 |
| 280 | 69046 | pectin lyase | 3 | 10.99% | 39542.92 | 5.26 |
| 281 | 36978 | allantoicase | 3 | 7.24% | 39355.16 | 5.24 |
| 283 | 66059 | uncharacterized protein | 3 | 5.38% | 67717.44 | 8.45 |
| 285 | 104656 | nucleosome assembly protein | 3 | 7.80% | 46706.19 | 4.36 |
| 286 | 94681 | RNA binding effector protein Scp160 | 3 | 2.72% | 141076.31 | 6.67 |
| 289 | 64470 | uncharacterized protein | 3 | 12.07% | 30330.59 | 5.17 |
| 290 | 85066 | uncharacterized protein | 3 | 8.51% | 35276.3 | 6.14 |
| 291 | 76728 | ribosomal protein S18 | 3 | 17.83% | 17877.58 | 10.48 |
| 293 | 23480 | Molecular co-chaperone STI1 | 3 | 4.64% | 65027.47 | 5.44 |
| 295 | 83493 | myo-inositol-phosphate synthase | 3 | 4.46% | 60252.69 | 5.75 |
| 296 | 106685 | DNA damage-inducible protein 1 | 3 | 9.02% | 40158.68 | 4.92 |
| 297 | 93023 | proteasome subunit alpha type 3 | 3 | 11.26% | 31893.73 | 5.12 |
| 298 | 60556 | uncharacterized protein | 3 | 7.33% | 37523.47 | 4.92 |
| 299 | 34610 | pyruvate dehydrogenase complex dihydrolipoamide acetyltransferase | 3 | 8.41% | 43722.56 | 5.3 |
| 339 | 88888 | uncharacterized protein | 2 | 8.79% | 29103.25 | 6.19 |
| 356 | 85437 | 2-deoxy-D-gluconate 3-dehydrogenase | 2 | 6.84% | 27647.49 | 6.59 |
| 363 | 88555 | tubulin alpha-2 | 2 | 4.21% | 49998.49 | 4.93 |
| 368 | 100924 | ATP synthase delta chain, mitochondrial precursor | 2 | 13.86% | 17680.17 | 5.27 |
| 369 | 86006 | vacuolar membrane ATPase-1 | 2 | 1.98% | 116746.79 | 6.14 |
| 375 | 95748 | transformer-SR ribonucleoprotein | 2 | 17.14% | 19068.79 | 6.18 |
| 381 | 76528 | metacaspase | 2 | 8.15% | 35575.56 | 4.64 |
| 383 | 78289 | Glutathione reductase | 2 | 3.82% | 50873.11 | 6.1 |
| 389 | 40323 | small nuclear ribonucleoprotein | 2 | 21.80% | 13986.53 | 9.99 |
| 391 | 74508 | helicase-like transcription factor HLTF/DNA helicase RAD5, DEAD-box superfamily | 2 | 2.68% | 116155.15 | 5.95 |
| 392 | 87412 | Glycyl-tRNA synthetase and related class II tRNA synthetase | 2 | 3.92% | 74380.71 | 5.98 |
| 393 | 75077 | uncharacterized protein | 2 | 8.59% | 28213.08 | 5.51 |
| 394 | 102269 | glutathione S-transferase | 2 | 7.82% | 27334.24 | 5.57 |
| 395 | 53103 | acetyltransferase | 2 | 8.50% | 22088.3 | 5.4 |
| 399 | 76821 | threonine dehydrogenase and related Zn-dependent dehydrogenases | 2 | 5.63% | 38576.55 | 5.95 |
| 402 | 93643 | leukotriene A4 hydrolase | 2 | 3.13% | 68459.56 | 5.17 |
| 404 | 89198 | 40S ribosomal protein S23 | 2 | 12.33% | 15855.45 | 10.62 |
| 406 | 85006 | peroxisomal copper amine oxidase | 2 | 2.46% | 77386.46 | 5.71 |
| 407 | 93182 | peptidase | 2 | 4.50% | 45147.14 | 4.97 |
| 408 | 93056 | 60S ribosomal protein L11 | 2 | 12.14% | 19845.92 | 10.36 |
| 410 | 74434 | uncharacterized protein | 2 | 2.18% | 126194.76 | 5.07 |
| 411 | 90424 | Lysophospholipase NTE1 | 2 | 1.81% | 163957 | 8.71 |
| 412 | 91056 | calcium P-type ATPase-2 | 2 | 6.70% | 23294.36 | 6.24 |
| 413 | 102189 | uncharacterized protein | 2 | 6.71% | 30894.2 | 5.21 |
| 414 | 103522 | uncharacterized protein | 2 | 3.44% | 54290.02 | 4.57 |
| 415 | 79472 | Short-chain dehydrogenase/reductase SDR | 2 | 11.33% | 26664.11 | 6.2 |
| 416 | 78615 | 60S ribosomal protein L3 | 2 | 4.08% | 44492.4 | 10.12 |
| 417 | 91022 | ATP phosphoribosyltransferase | 2 | 5.11% | 35767.04 | 5.52 |
| 419 | 78182 | aldo-keto reductase | 2 | 5.52% | 38161.66 | 5.48 |
| 420 | 97529 | uncharacterized protein | 2 | 3.48% | 60287.39 | 9.31 |
| 421 | 82322 | uncharacterized protein | 2 | 6.98% | 37299 | 3.83 |
| 422 | 101959 | 2-keto-4-pentenoate hydratase/2-oxohepta-3-ene-1,7-dioic acid hydratase | 2 | 10.31% | 31223.84 | 5.2 |
| 424 | 103600 | uncharacterized protein | 2 | 13.33% | 18103.26 | 5.18 |
| 425 | 93464 | 60S ribosomal protein L10-A-like protein | 2 | 6.36% | 25098.19 | 10.33 |
| 426 | 45843 | phosphatidylinositol 4-kinase | 2 | 0.77% | 216401.89 | 6.13 |
| 427 | 77366 | synaptic vesicle protein EHS-1 and related EH domain proteins | 2 | 2.01% | 159784.34 | 5.66 |
| 428 | 50713 | uncharacterized protein | 2 | 5.45% | 45925.96 | 4.75 |
| 429 | 60508 | tryptophan synthetase | 2 | 3.35% | 77207.22 | 5.86 |
| 430 | 92679 | 20S proteasome, regulatory subunit beta | 2 | 6.42% | 29035.96 | 6.24 |
| 431 | 104570 | Voltage-gated potassium channel beta-2 subunit | 2 | 5.04% | 39582.91 | 6.28 |
| 432 | 78195 | manganese superoxide dismutase | 2 | 9.40% | 25251.37 | 7.98 |
| 433 | 42705 | phosphoribosylaminoimidazolecarboxamide formyltransferase/IMP cyclohydrolase | 2 | 3.83% | 65418.48 | 5.93 |
| 434 | 78867 | Ankyrin repeat protein nuc-2 | 2 | 1.28% | 154518.25 | 5.32 |
| 435 | 84731 | Class E vacuolar protein-sorting machinery protein HSE1 | 2 | 4.44% | 66219.02 | 5.75 |
| 436 | 89477 | NADH oxidase | 2 | 4.67% | 49688.68 | 5.97 |
| 437 | 62595 | malate dehydrogenase | 2 | 2.98% | 66864.15 | 5.26 |
| 438 | 95965 | dipeptidyl peptidase | 2 | 3.15% | 76571.25 | 5.97 |
| 439 | 102411 | Tubulin alpha chain (Alpha-tubulin) | 2 | 4.17% | 50228.53 | 4.95 |
| 440 | 72073 | uncharacterized protein | 2 | 7.64% | 33985.59 | 7.54 |
| 441 | 105743 | glutamyl-tRNA synthetase | 2 | 2.34% | 72826.92 | 6.57 |
| 442 | 52580 | 40S ribosomal protein S13 | 2 | 8.55% | 16809.71 | 10.35 |
| 443 | 87479 | 60S ribosomal protein L17 | 2 | 10.16% | 20937.1 | 10.76 |
| 444 | 98228 | uncharacterized protein | 2 | 6.79% | 28907.27 | 5.81 |
| 445 | 108860 | 3-deoxy-D-arabino-heptulosonate 7-phosphate (DAHP) synthase | 2 | 6.90% | 40500.16 | 5.9 |
| 446 | 98393 | threonyl-tRNA synthetase | 2 | 7.81% | 53368.11 | 8.93 |
| 447 | 33193 | uncharacterized protein | 2 | 1.92% | 121810.24 | 5.89 |
| 448 | 107026 | Chitinase | 2 | 1.03% | 241101.62 | 7.13 |
| 449 | 97535 | uncharacterized protein | 2 | 3.40% | 84914.59 | 8.81 |
| 450 | 53732 | 40S ribosomal protein S22 | 2 | 12.21% | 14807.26 | 10.1 |
| 451 | 37664 | heat shock protein 70-like protein | 2 | 3.46% | 110670.37 | 5.26 |
| 452 | 48675 | DNA polymerase epsilon, catalytic subunit A/POL2 | 2 | 0.96% | 249547.13 | 6.51 |
| 453 | 88187 | uncharacterized protein | 2 | 6.61% | 35509.08 | 5.92 |
| 454 | 109515 | isoamyl alcohol oxidase | 2 | 4.08% | 63446.37 | 4.55 |
| 455 | 63274 | regulatory particle ATPase-like-10 | 2 | 5.12% | 31701.49 | 5.13 |
| 456 | 69333 | uncharacterized protein | 2 | 6.31% | 55484.64 | 9.58 |
| 457 | 42468 | Secreted enzyme, contains two amidohydrolase related domains | 2 | 2.41% | 106890.59 | 5.47 |
| 458 | 79225 | related to gamma-glutamyltransferase | 2 | 3.68% | 61706.95 | 5.46 |
| 459 | 37583 | hybrid polyketide synthase/nonribosomal peptide synthase | 2 | 1.38% | 278166.63 | 5.58 |
| 460 | 79314 | inositol monophosphatase | 2 | 4.73% | 33415.67 | 4.95 |
| 461 | 99601 | alanyl-tRNA synthetase | 2 | 7.67% | 33723.14 | 6 |
| 462 | 45469 | manganese peroxidase | 2 | 11.65% | 25811.62 | 4.19 |
| 463 | 74131 | aldehyde reductase | 2 | 7.78% | 27997.14 | 6.4 |
| 464 | 94653 | related to AP-1-like transcription factor | 2 | 4.17% | 62056.4 | 5.01 |
| 465 | 88134 | uncharacterized protein | 2 | 4.84% | 54446.23 | 5.77 |
| 466 | 87187 | dehydrogenase complex subunit alpha | 2 | 4.62% | 45397.6 | 8.15 |
| 478 | 109096 | extracellular lipase | 1 | 1.22% | 61869.34 | 4.49 |
| 489 | 65110 | ngg1-interacting factor 3 | 1 | 2.88% | 33470.13 | 5.32 |
| 493 | 19348 | uncharacterized protein | 1 | 6.45% | 16369.7 | 3.79 |
| 496 | 107760 | ribulose-phosphate 3-epimerase | 1 | 3.17% | 27282.21 | 5.49 |
| 500 | 96428 | peptidylprolyl isomerase | 1 | 4.47% | 19555.79 | 6.32 |
| 501 | 100900 | proteasome component PRE6 | 1 | 4.41% | 29585.66 | 8.63 |
| 502 | 96843 | autophagy related lipase Atg15 | 1 | 1.43% | 68028.36 | 5.38 |
| 503 | 93970 | single-stranded DNA-binding replication protein A (RPA), large (70 kD) subunit and related ssDNA-binding proteins | 1 | 1.81% | 67545.13 | 5.98 |
| 504 | 50662 | 26S proteasome regulatory subunit | 1 | 2.03% | 54421.97 | 6.39 |
| 505 | 97972 | Acyl-CoA desaturase | 1 | 1.89% | 53771.47 | 9.26 |
| 506 | 69352 | sodium P-type ATPase | 1 | 0.63% | 121081.67 | 7.26 |
| 507 | 27117 | uncharacterized protein | 1 | 3.68% | 29220.97 | 4.22 |
| 508 | 64901 | GTPase-activating protein | 1 | 3.07% | 56621.2 | 9.11 |
| 509 | 60154 | nitrilase | 1 | 2.13% | 45400.91 | 5.29 |
| 511 | 95284 | dihydrolipoamide dehydrogenase | 1 | 1.98% | 53761.69 | 6.62 |
| 512 | 74826 | Endo-1,6-beta-D-glucanase | 1 | 1.86% | 50986.76 | 3.99 |
| 513 | 88018 | GTPase activating protein | 1 | 0.49% | 180357.93 | 5.64 |
| 515 | 86693 | multidrug/pheromone exporter, ABC superfamily | 1 | 0.91% | 143591.92 | 5.46 |
| 516 | 106187 | Cytochrome b-c1 complex subunit 2 | 1 | 1.95% | 48543.11 | 8.93 |
| 517 | 47867 | ATP-dependent RNA helicase | 1 | 0.79% | 168511.25 | 6.54 |
| 518 | 78153 | Short-chain dehydrogenase/reductase SDR | 1 | 3.02% | 31956.26 | 6.76 |
| 519 | 104977 | O-methyltransferase | 1 | 3.61% | 27292.12 | 5.79 |
| 520 | 45028 | laccase | 1 | 1.71% | 63298.42 | 5.6 |
| 521 | 69033 | HAD-superfamily hydrolase subfamily IIB | 1 | 3.60% | 27630.4 | 5.34 |
| 522 | 100822 | 60S ribosomal protein L32 | 1 | 6.06% | 15062.62 | 11.36 |
| 523 | 84883 | aldo-keto reductase | 1 | 2.61% | 38149.42 | 5.67 |
| 524 | 86909 | protein phosphatase 2A regulatory subunit A and related proteins | 1 | 1.44% | 69253.18 | 4.8 |
| 525 | 35539 | oxidoreductase | 1 | 4.53% | 33049.57 | 8.98 |
| 526 | 77754 | Serine/threonine-protein phosphatase 2B catalytic subunit | 1 | 2.11% | 65085.05 | 5.36 |
| 527 | 105253 | Protein VTS1 | 1 | 1.45% | 65722.52 | 8.54 |
| 528 | 91736 | aldo-keto reductase | 1 | 2.64% | 32947.07 | 5.2 |
| 529 | 100780 | 60S ribosomal protein L7 | 1 | 3.60% | 28724.57 | 9.98 |
| 530 | 102251 | aldo-keto reductase | 1 | 3.42% | 38892.13 | 6.28 |
| 531 | 74707 | nucleoside hydrolase | 1 | 2.60% | 41866.11 | 4.34 |
| 532 | 105955 | Leucyl-tRNA synthetase | 1 | 0.80% | 125958.55 | 6.24 |
| 533 | 54469 | NADPH:quinone reductase and related Zn-dependent oxidoreductases | 1 | 4.29% | 34565.63 | 7.08 |
| 534 | 101909 | Pyridoxine biosynthesis protein PDX1 | 1 | 6.30% | 24838.3 | 6.12 |

Supplementary Table S-5 The identified proteins from fresh EP medium by HPLC-ESI-OrbiTrap MSMS .

| **No.** | **Accession No.^a^** | **Protein Name** | **Unique PepCount^c^** | **Cover Percent^d^** | **MW(kDa)^e^** | **pI^f^** |
| --- | --- | --- | --- | --- | --- | --- |
| 1 | 81935 | Rho guanyl nucleotide exchange factor | 2 | 10.20% | 140.7 | 8.92 |
| 2 | 88574 | Rho guanyl nucleotide exchange factor | 1 | 4.51% | 112.8 | 7.03 |
| 3 | 102864* | Elongation factor 1-alpha (EF-1-alpha) | 1 | 4.74% | 50.5 | 9.15 |

a Accession number from *Cryphonectria parasitica* database v1.0.

c The total number of unique matched peptides to the identified protein.

d The cover percentage of matched peptides to the protein sequence.

e Theoretical molecular weight.

f Theoretical pI.

* This protein was also identified in secreted samples with no significantly change upon hypovirus infection.

Supplementary Table S-6 Annotation of fungal secretomes by Fungal Secretome Database (FSD).

a Accession number from Cryphonectria parasitica database v1.0.

b There were five groups were described according to FSD platform. Proteins (87) which appeared in Class SP were predicted by SignalP 3.0. In other words, these proteins all got a signal peptide. Proteins (197) which appeared in Class SP3 were predicted by SigPred, SigCleave, or RPSP. Proteins (22) which appeared in Class SL were predicted by PSort II or TargetP 1.1, but were not predicted by SignalP 3.0, SigPred, SigCleave or RPSP. Proteins (23) predicted by SecretomeP 1.0 which was summarized as Class NS in FSD platform. Noneffective predicted proteins (74) were summarized in the class of unknown.

| **Protein Name** | **Accession No.^a^** | **Class ^b^** |
| --- | --- | --- |
| 3-isopropylmalate dehydratase | 40873 | NS |
| 3-isopropylmalate dehydrogenase (Beta-IPM dehydrogenase) (IMDH) (3-IPM-DH) | 96560 | NS |
| 40S ribosomal protein S0 (Ribosome-associated protein 1) | 101628 | NS |
| 40S ribosomal protein S2-like protein | 103050 | NS |
| 60S ribosomal protein L2 | 87233 | NS |
| Cytochrome c | 106144 | NS |
| Elongation factor 1-beta | 102227 | NS |
| Hydroxymethylglutaryl-CoA synthase | 84488 | NS |
| nucleoside diphosphate kinase-1(NDK-1) | 78037 | NS |
| Outer mitochondrial membrane protein porin | 99283 | NS |
| Replication factor-A protein | 69552 | NS |
| RHO protein GDP dissociation inhibitor(RDI) regulation of growth rate | 40411 | NS |
| Ribose 5-phosphate isomerase-like protein | 55273 | NS |
| Rnapii degradation factor | 106307 | NS |
| Rossmann-fold NAD(P)(+)-binding protein | 102665 | NS |
| Thiamin pyrophosphokinase | 36141 | NS |
| uncharacterized protein | 40356 | NS |
| uncharacterized protein | 96317 | NS |
| uncharacterized protein | 102261 | NS |
| uncharacterized protein | 105107 | NS |
| uncharacterized protein | 105126 | NS |
| uncharacterized protein | 108086 | NS |
| uncharacterized protein | 108594 | NS |
| 2-nitropropane dioxygenase | 33445 | SL |
| 40S ribosomal protein S4 | 55625 | SL |
| 60S ribosomal protein L5 (CPR4) | 83235 | SL |
| 6-phosphogluconate dehydrogenase | 92891 | SL |
| Adenosylhomocysteinase | 76097 | SL |
| Aldose 1-epimerase | 49385 | SL |
| Argininosuccinate synthase | 63408 | SL |
| Calmodulin (CaM) | 58417 | SL |
| Carbon-nitrogen hydrolase | 63766 | SL |
| Cystathionine beta-synthase | 77086 | SL |
| Glucosamine-fructose-6-phosphate aminotransferase | 85870 | SL |
| L-xylulose reductase (XR) | 94341 | SL |
| Microtubule-associated protein RP/EB family member 1 | 76520 | SL |
| NAD dependent epimerase/dehydratase | 102577 | SL |
| phosphoglycerate kinase | 101915 | SL |
| Ribokinase-like protein | 41127 | SL |
| Serine hydroxymethyltransferase, cytosolic (Serine methylase) (Glycine hydroxymethyltransferase) (SHMT) | 48583 | SL |
| uncharacterized protein | 58481 | SL |
| uncharacterized protein | 64397 | SL |
| uncharacterized protein | 82249 | SL |
| uncharacterized protein | 100994 | SL |
| uncharacterized protein | 102031 | SL |
| 1,3-beta-glucanosyltransferase gel1 precursor (Glucan elongating glucanosyltransferase 1) | 63707 | SP |
| 1,3-beta-glucanosyltransferase gel2 precursor (Glucan elongating glucanosyltransferase 2) | 108828 | SP |
| 22kDa glycoprotein | 100735 | SP |
| 3-phytase A precursor (Myo-inositol-hexaphosphate 3-phosphohydrolase A) (3 phytase A) (Myo-inositol hexakisphosphate phosphohydrolase A) | 79318 | SP |
| 60S ribosomal protein L13 | 94953 | SP |
| 60S ribosomal protein L23 | 55968 | SP |
| 78 kDa glucose-regulated protein homolog precursor (GRP 78) | 84573 | SP |
| Aconitate hydratase | 99714 | SP |
| actin-3(regulated by circadian rhythms/ vacuole inheritance) | 88045 | SP |
| Agmatinase | 33751 | SP |
| Aldo/keto reductase | 63094 | SP |
| Alpha amylase | 84745 | SP |
| Alpha-glucosidase precursor (Maltase) | 43693 | SP |
| Amidohydrolase | 94542 | SP |
| Aminopeptidase Y | 99702 | SP |
| Aorsin precursor, serine proteinase with trypsin-like specificity at acidic pH | 91219 | SP |
| Arylsulfatase | 95432 | SP |
| Aspergillopepsin | 103361 | SP |
| ATP synthase subunit 5, mitochondrial precursor (Oligomycin sensitivity conferral protein) (OSCP) (ATP synthase chain 5) | 88657 | SP |
| ATP-dependent DNA helicase II, 70 kDa subunit | 69709 | SP |
| b-1,3-glucanosyltransferase; CBM43 module; GPI-anchor , secreted | 104818 | SP |
| Beta-fructofuranosidase | 107379 | SP |
| Calnexin homolog precursor | 107659 | SP |
| Carboxylesterase | 97118 | SP |
| Carboxypeptidase S1 | 108972 | SP |
| catalase/peroxidase | 76127 | SP |
| Catalytic activity: deacetylation of xylans and xylo-oligosaccharides | 70238 | SP |
| Cell wall protein PhiA | 103814 | SP |
| Cellobiose dehydrogenase | 39110 | SP |
| Cutinase precursor (Cutin hydrolase) | 108053 | SP |
| Endoglucanase-4 precursor (Endoglucanase IV) (Endo-1,4-beta-glucanase IV) (Cellulase IV) (EGIV) | 95002 | SP |
| Endopolygalacturonase 1 precursor (Pectinase) (Clpg1) | 36288 | SP |
| Endothiapepsin precursor (Aspartate protease) | 94005 | SP |
| Endothiapepsin precursor (Aspartate protease) | 100383 | SP |
| Endothiapepsin precursor (Aspartate protease) | 107093 | SP |
| enolase | 103155 | SP |
| Exoglucanase 2 precursor (Exoglucanase II) (Exocellobiohydrolase II) (CBHII) (1,4-beta-cellobiohydrolase) | 52977 | SP |
| Extracellular carboxypeptidase | 100631 | SP |
| Extracellular cell wall glucanase | 99792 | SP |
| Extracellular chitinase | 105629 | SP |
| Extracellular lipase | 105554 | SP |
| F-actin-capping protein subunit alpha | 94802 | SP |
| FAD binding domain containing protein | 87599 | SP |
| FAD binding domain-containing protein | 109500 | SP |
| FK506 resistant-2(FKR-2)(Peptidyl-prolyl cis-trans isomerase) (PPIase) (Rotamase) (FKBP-21) (NcFKBP22) | 109525 | SP |
| GPI-anchored cell wall beta-1,3-endoglucanase | 89436 | SP |
| GPI-anchored cell wall beta-1,3-endoglucanase EglC | 67775 | SP |
| heat shock protein 80(Suppressor of vegetative incompatibility MOD-E) | 87995 | SP |
| HSP70-1 | 79019 | SP |
| Laccase | 83754 | SP |
| Malate dehydrogenase | 89617 | SP |
| Peptidyl-prolyl cis-trans isomerase B precursor (PPIase B) (Rotamase B) | 58361 | SP |
| Phosphatidylglycerol/phosphatidylinositol transfer protein precursor (PG/PI-TP) | 102078 | SP |
| Prenylcysteine oxidase | 74071 | SP |
| Protein disulfide-isomerase erp38 precursor (ERp38) | 100991 | SP |
| Protein disulfide-isomerase precursor (PDI) | 99052 | SP |
| Ribonuclease T2-like precursor (RNase T2-like) | 93764 | SP |
| Serine-type peptidase | 84160 | SP |
| Similar to Osmotic growth protein 1 | 23873 | SP |
| Subtilisin-like proteinase | 102797 | SP |
| Superoxide dismutase-1(SOD-1)[Cu-Zn] | 80754 | SP |
| Survival protein sure-like phosphatase/nucleotidase-like protein | 103771 | SP |
| Tripeptidyl peptidase | 64622 | SP |
| uncharacterized protein | 35666 | SP |
| uncharacterized protein | 51266 | SP |
| uncharacterized protein | 53221 | SP |
| uncharacterized protein | 59332 | SP |
| uncharacterized protein | 67753 | SP |
| uncharacterized protein | 70476 | SP |
| uncharacterized protein | 70492 | SP |
| uncharacterized protein | 74613 | SP |
| uncharacterized protein | 74957 | SP |
| uncharacterized protein | 91966 | SP |
| uncharacterized protein | 92044 | SP |
| uncharacterized protein | 93265 | SP |
| uncharacterized protein | 93693 | SP |
| uncharacterized protein | 93998 | SP |
| uncharacterized protein | 97441 | SP |
| uncharacterized protein | 103448 | SP |
| uncharacterized protein | 103631 | SP |
| uncharacterized protein | 104348 | SP |
| uncharacterized protein | 107212 | SP |
| uncharacterized protein | 107223 | SP |
| uncharacterized protein | 107233 | SP |
| uncharacterized protein | 107537 | SP |
| UTH1 | 85799 | SP |
| vacuolar protease A precursor(PEP-4) | 83961 | SP |
| 14-3-3 protein | 102966 | SP^3^ |
| 14-3-3-like protein | 102916 | SP^3^ |
| 2,3-dihydroxybenzoic acid decarboxylase (DHBD) (o-pyrocatechuate decarboxylase) (2,3-DHBA decarboxylase) | 49665 | SP^3^ |
| 3',5'-bisphosphate nucleotidase | 103985 | SP^3^ |
| 3-hydroxyacyl-CoA dehydrogenase | 105574 | SP^3^ |
| 40S ribosomal protein S10 | 101666 | SP^3^ |
| 40S ribosomal protein S14 (CRP2) | 100785 | SP^3^ |
| 40S ribosomal protein S5 | 79283 | SP^3^ |
| 40S ribosomal protein S7 | 100503 | SP^3^ |
| 41 kDa peptidyl-prolyl cis-trans isomerase (PPIase) (Rotamase) (Cyclophilin-41) (CYP-41) | 47560 | SP^3^ |
| 4-aminobutyrate aminotransferase | 58903 | SP^3^ |
| 60S acidic ribosomal protein P0 | 103140 | SP^3^ |
| 60S ribosomal protein L12 | 75598 | SP^3^ |
| 60S ribosomal protein L4 | 105502 | SP^3^ |
| 60S ribosomal protein L6 | 43297 | SP^3^ |
| Aado/keto reductase | 31781 | SP^3^ |
| Acetyl-CoA acetyltransferase | 107307 | SP^3^ |
| Actin cytoskeleton protein (VIP1) | 104024 | SP^3^ |
| Adenosine kinase | 35264 | SP^3^ |
| ADP-ribosylation factor | 102464 | SP^3^ |
| Alanine racemase TOXG | 95427 | SP^3^ |
| Alanine-glyoxylate aminotransferase | 38338 | SP^3^ |
| Alanyl-tRNA synthetase | 107789 | SP^3^ |
| Alcohol dehydrogenase | 91746 | SP^3^ |
| alcohol dehydrogenase | 97558 | SP^3^ |
| Alcohol dehydrogenase-1(ADH-1) | 106275 | SP^3^ |
| Aldose-1-epimerase | 81476 | SP^3^ |
| Aminopeptidase 2 | 82725 | SP^3^ |
| AP-1 complex subunit gamma-1 | 52243 | SP^3^ |
| Arginase | 99673 | SP^3^ |
| Argonaute-like protein. the catalytic components of the RNA-induced silencing complex (RISC), the protein complex responsible for the gene silencing phenomenon known as RNA interference (RNAi). | 84041 | SP^3^ |
| Aryl-alcohol oxidase | 52735 | SP^3^ |
| Aspartate aminotransferase | 68371 | SP^3^ |
| Aspartate-semialdehyde dehydrogenase | 71516 | SP^3^ |
| Aspartyl aminopeptidase | 36435 | SP^3^ |
| Aspartyl-tRNA synthetase | 80744 | SP^3^ |
| ATP citrate lyase | 88317 | SP^3^ |
| ATP synthase subunit alpha, mitochondrial precursor | 82931 | SP^3^ |
| ATP synthase subunit beta, mitochondrial precursor | 88762 | SP^3^ |
| ATP-citrate synthase subunit 1 (ATP-citrate (pro-S-)-lyase 1) (Citrate cleavage enzyme subunit 1) | 78256 | SP^3^ |
| ATP-dependent RNA helicase eIF4A (Eukaryotic initiation factor 4A) (eIF-4A) (Translation initiation factor 1) | 92811 | SP^3^ |
| cell division control protein Cdc48 | 77211 | SP^3^ |
| Cell wall protein AWA1 precursor | 69130 | SP^3^ |
| Chaperone protein dnaK | 107004 | SP^3^ |
| Coproporphyrinogen III oxidase | 97515 | SP^3^ |
| Cyanide hydratase (Formamide hydrolyase) | 84265 | SP^3^ |
| Cyclopentanol dehydrogenase | 43065 | SP^3^ |
| Cytochrome b-c1 complex subunit Rieske, mitochondrial precursor (Ubiquinol-cytochrome c reductase iron-sulfur subunit) (Rieske iron-sulfur protein) (RISP) (Complex III subunit 5) | 102157 | SP^3^ |
| Cytochrome c oxidase polypeptide IV, mitochondrial precursor | 80629 | SP^3^ |
| cytoplasmic ribosomal protein-(CRP-10)Cytoplasmic ribosomal protein subunit S3 | 86878 | SP^3^ |
| D-3-phosphoglycerate dehydrogenase | 52030 | SP^3^ |
| Deoxyuridine 5'-triphosphate nucleotidohydrolase (dUTPase) (dUTP pyrophosphatase) | 99093 | SP^3^ |
| Dienelactone hydrolase | 73563 | SP^3^ |
| Dihydrolipoyllysine-residue acetyltransferase component of pyruvate dehydrogenase complex, mitochondrial precursor (E2) (Dihydrolipoamide acetyltransferase component of pyruvate dehydrogenase complex) (PDC-E2) (MRP3) | 40976 | SP^3^ |
| dihydroxy-acid dehydratase | 59925 | SP^3^ |
| Dipeptidase | 35302 | SP^3^ |
| Dipeptidyl peptidase | 81065 | SP^3^ |
| Diphosphomevalonate decarboxylase | 38677 | SP^3^ |
| D-lactate dehydrogenase | 104856 | SP^3^ |
| EF hand domain-containing protein | 105958 | SP^3^ |
| Elongation factor 1-alpha (EF-1-alpha) | 102864 | SP^3^ |
| Elongation factor 1-gamma | 95810 | SP^3^ |
| Elongation factor 2 (EF-2) | 90536 | SP^3^ |
| Elongation factor 3 | 79471 | SP^3^ |
| Endothelin-converting enzyme | 94251 | SP^3^ |
| Epoxide hydrolase | 100903 | SP^3^ |
| Eukaryotic translation initiation factor 3 subunit D (eIF3d) | 63085 | SP^3^ |
| Eukaryotic translation initiation factor 3 subunit F (eIF3f) | 93992 | SP^3^ |
| Eukaryotic translation initiation factor 5A-2 (eIF-5A-2) (eIF-4D) (Hypusine-containing protein HP2) | 102068 | SP^3^ |
| FAD dependent oxidoreductase | 99253 | SP^3^ |
| Fatty acid synthase subunit alpha [Includes: Acyl carrier; 3-oxoacyl-[acyl-carrier-protein] reductase (Beta-ketoacyl reductase); 3-oxoacyl-[acyl-carrier-protein] synthase (Beta-ketoacyl synthase)] | 70070 | SP^3^ |
| Formate dehydrogenase (NAD-dependent formate dehydrogenase) (FDH) | 87808 | SP^3^ |
| Formyltetrahydrofolate deformylase | 96214 | SP^3^ |
| Glucose-6-phosphate isomerase (GPI) | 89763 | SP^3^ |
| Glutamate carboxypeptidase | 93457 | SP^3^ |
| Glyceraldehyde-3-phosphate dehydrogenase (GAPDH) | 101684 | SP^3^ |
| glycogen phosphorylase | 69531 | SP^3^ |
| GMC oxidoreductase | 89863 | SP^3^ |
| GTP-binding nuclear protein GSP1/Ran | 43908 | SP^3^ |
| Guanine nucleotide-binding protein subunit beta-like protein (Cross-pathway control WD-repeat protein cpc-2) | 103579 | SP^3^ |
| Heat shock protein 60 | 88770 | SP^3^ |
| Heat shock protein Hsp88 | 80500 | SP^3^ |
| Hexokinase | 92971 | SP^3^ |
| high mobility group protein | 91265 | SP^3^ |
| Histidine biosynthesis trifunctional protein [Includes: Phosphoribosyl-AMP cyclohydrolase ; Phosphoribosyl-ATP pyrophosphohydrolase ; Histidinol dehydrogenase (HDH)] | 107162 | SP^3^ |
| Homoserine acetyltransferase | 95090 | SP^3^ |
| Homoserine dehydrogenase | 99754 | SP^3^ |
| Hsp70 chaperone | 91280 | SP^3^ |
| Hsp70-like protein | 61733 | SP^3^ |
| importin beta-4(regulated by circadian rhythms/regulation of mitotic cell cycle) | 100977 | SP^3^ |
| Inorganic pyrophosphatase (Pyrophosphate phospho-hydrolase) (PPase) | 98830 | SP^3^ |
| Inosine-5'-monophosphate dehydrogenase | 98458 | SP^3^ |
| Isocitrate dehydrogenase | 90560 | SP^3^ |
| Isocitrate dehydrogenase [NADP] | 63966 | SP^3^ |
| Isocitrate lyase | 61858 | SP^3^ |
| Lactoylglutathione lyase | 65706 | SP^3^ |
| Leucine permease transcriptional regulator | 105464 | SP^3^ |
| Malate dehydrogenase | 100550 | SP^3^ |
| Methionine aminopeptidase | 93442 | SP^3^ |
| methionine synthase | 88329 | SP^3^ |
| Mitochondrial peroxiredoxin PRX1 | 82471 | SP^3^ |
| Monodehydroascorbate reductase | 99412 | SP^3^ |
| mRNA binding post-transcriptional regulator | 93073 | SP^3^ |
| MYG1 protein | 57041 | SP^3^ |
| N-acetylglucosamine-phosphate mutase | 98281 | SP^3^ |
| NAD(P)-binding protein | 93941 | SP^3^ |
| NAD(P)-binding protein | 103641 | SP^3^ |
| NADH:flavin oxidoreductase/NADH oxidase family protein | 50968 | SP^3^ |
| NADH-cytochrome b5 reductase | 107257 | SP^3^ |
| NADP-dependent leukotriene B4 12-hydroxydehydrogenase | 64525 | SP^3^ |
| NADPH:quinone reductase and related Zn-dependent oxidoreductases | 77940 | SP^3^ |
| Nascent polypeptide-associated complex subunit beta (NAC-beta) (Beta-NAC) | 102329 | SP^3^ |
| Nitroreductase | 96894 | SP^3^ |
| Nuclear localization sequence binding protein | 72340 | SP^3^ |
| Nuclear movement protein nudC | 63198 | SP^3^ |
| O-methyltransferase | 45332 | SP^3^ |
| Ornithine aminotransferase (Ornithine--oxo-acid aminotransferase) | 86840 | SP^3^ |
| Peptidase family M20/M25/M40 | 49897 | SP^3^ |
| Peptidyl-prolyl cis-trans isomerase H (PPIase H) (Rotamase H) | 43362 | SP^3^ |
| Peptidyl-prolyl cis-trans isomerase, mitochondrial precursor (PPIase) (Rotamase) (Cyclophilin) (Cyclosporin A-binding protein) (CPH) | 55987 | SP^3^ |
| Peroxiredoxin | 43719 | SP^3^ |
| Phosphatidyl inositol-specific phospholipase C, cplc1 (delta-type PLC) | 86663 | SP^3^ |
| phosphoglucomutase | 102434 | SP^3^ |
| phosphoglycerate mutase, 2,3-bisphosphoglycerate-independent | 94119 | SP^3^ |
| Polyadenylate-binding protein, cytoplasmic and nuclear (Poly(A)-binding protein) (PABP) (Polyadenylate tail-binding protein) | 64909 | SP^3^ |
| Polygalacturonase-3 precursor (Polygalacturonase III) (PG-III) (PGC) (Pectinase-3) | 65827 | SP^3^ |
| Porphobilinogen deaminase (PBG) (Hydroxymethylbilane synthase) (HMBS) (Pre-uroporphyrinogen synthase) | 34543 | SP^3^ |
| Proliferating cell nuclear antigen | 63427 | SP^3^ |
| Proteasome subunit alpha type-1 | 34049 | SP^3^ |
| proteasome subunit alpha type-2 | 88636 | SP^3^ |
| Protein HMF1 | 55727 | SP^3^ |
| Protein kinase C(CPKC) | 89402 | SP^3^ |
| Pyruvate carboxylase (Pyruvic carboxylase) (PCB) | 82952 | SP^3^ |
| Pyruvate decarboxylase (8-10 nm cytoplasmic filament-associated protein) | 79606 | SP^3^ |
| Pyruvate kinase | 42922 | SP^3^ |
| Quinone oxidoreductase | 91352 | SP^3^ |
| Rab GDP-dissociation inhibitor | 80153 | SP^3^ |
| Related to stress protein p66 | 73267 | SP^3^ |
| Ribonucleoside-diphosphate reductase small chain (Ribonucleotide reductase small subunit) | 66428 | SP^3^ |
| Saccharopine dehydrogenase [NADP+, L-glutamate-forming] (Saccharopine reductase) | 100169 | SP^3^ |
| S-adenosylmethionine synthetase (Methionine adenosyltransferase) (AdoMet synthetase) | 85769 | SP^3^ |
| Sec14 cytosolic factor | 85234 | SP^3^ |
| Sedoheptulose-1,7-bisphosphatase | 62138 | SP^3^ |
| Serine/threonine protein kinase | 99084 | SP^3^ |
| seryl-tRNA synthetase | 65476 | SP^3^ |
| S-formylglutathione hydrolase | 59013 | SP^3^ |
| Short chain dehydrogenase | 69622 | SP^3^ |
| Sulfite reductase | 106622 | SP^3^ |
| Thioredoxin | 45775 | SP^3^ |
| Thioredoxin reductase | 72159 | SP^3^ |
| Threonyl-tRNA synthetase-like protein | 99715 | SP^3^ |
| transaldolase | 42419 | SP^3^ |
| Transketolase | 83897 | SP^3^ |
| triosephosphate isomerase(TPI) | 77119 | SP^3^ |
| Tubulin beta chain (Beta-tubulin) | 74392 | SP^3^ |
| Tubulin specific chaperone cofactor B | 88593 | SP^3^ |
| Ubiquitin C-terminal hydrolase | 73146 | SP^3^ |
| Ubiquitin-activating enzyme E1 1 | 80224 | SP^3^ |
| UDP-glucose 4-epimerase | 89596 | SP^3^ |
| UDP-N-acetylglucosamine pyrophosphorylase | 107213 | SP^3^ |
| UMP-CMP kinase | 71911 | SP^3^ |
| uncharacterized protein | 37540 | SP^3^ |
| uncharacterized protein | 38710 | SP^3^ |
| uncharacterized protein | 43898 | SP^3^ |
| uncharacterized protein | 47978 | SP^3^ |
| uncharacterized protein | 50954 | SP^3^ |
| uncharacterized protein | 64200 | SP^3^ |
| uncharacterized protein | 69051 | SP^3^ |
| uncharacterized protein | 69628 | SP^3^ |
| uncharacterized protein | 71110 | SP^3^ |
| uncharacterized protein | 74162 | SP^3^ |
| uncharacterized protein | 76452 | SP^3^ |
| uncharacterized protein | 76626 | SP^3^ |
| uncharacterized protein | 78120 | SP^3^ |
| uncharacterized protein | 79545 | SP^3^ |
| uncharacterized protein | 80991 | SP^3^ |
| uncharacterized protein | 81491 | SP^3^ |
| uncharacterized protein | 82357 | SP^3^ |
| uncharacterized protein | 83378 | SP^3^ |
| uncharacterized protein | 86899 | SP^3^ |
| uncharacterized protein | 88910 | SP^3^ |
| uncharacterized protein | 89229 | SP^3^ |
| uncharacterized protein | 90079 | SP^3^ |
| uncharacterized protein | 94757 | SP^3^ |
| uncharacterized protein | 95286 | SP^3^ |
| uncharacterized protein | 102177 | SP^3^ |
| uncharacterized protein | 102428 | SP^3^ |
| uncharacterized protein | 106534 | SP^3^ |
| uncharacterized protein | 107358 | SP^3^ |
| uncharacterized protein | 108424 | SP^3^ |
| UTP-glucose-1-phosphate uridylyltransferase | 42588 | SP^3^ |
| UV excision repair protein Rad23 | 25948 | SP^3^ |
| Vacuolar aminopeptidase 1 | 34225 | SP^3^ |
| vacuolar membrane ATPase-2(VMA-2)Vacuolar ATP synthase subunit B (V-ATPase subunit B) (Vacuolar proton pump subunit B) (V-ATPase 57 kDa subunit) | 101333 | SP^3^ |
| Valyl-tRNA synthetase, mitochondrial precursor (Valine--tRNA ligase) (ValRS) | 82721 | SP^3^ |
| Woronin body major protein precursor | 90806 | SP^3^ |
| Xaa-Pro aminopeptidase | 86554 | SP^3^ |
| 40S ribosomal protein S11 | 102565 | Unknown |
| 40S ribosomal protein S19 (S16) | 90732 | Unknown |
| 40S ribosomal protein S21 (CRP7) | 102630 | Unknown |
| 40S ribosomal protein S28 | 54024 | Unknown |
| 40S ribosomal protein S29 | 54042 | Unknown |
| 5-hydroxyisourate hydrolase | 47193 | Unknown |
| 6,7-dimethyl-8-ribityllumazine synthase (DMRL synthase) (Lumazine synthase) (Riboflavin synthase beta chain) | 82588 | Unknown |
| 60S acidic ribosomal protein P2 | 92473 | Unknown |
| 60S ribosomal protein L22 | 103302 | Unknown |
| 60S ribosomal protein L23 | 34291 | Unknown |
| 60S ribosomal protein L27 | 88903 | Unknown |
| 60S ribosomal protein L30 | 103417 | Unknown |
| 60S ribosomal protein L31 | 102482 | Unknown |
| 60S ribosomal protein L34 | 53829 | Unknown |
| BRT1 | 94557 | Unknown |
| Calmodulin (CaM) | 70319 | Unknown |
| clathrin light chain(regulated by circadian rhythms) | 85703 | Unknown |
| Cofilin (Actin-depolymerizing factor 1) | 88774 | Unknown |
| Eukaryotic translation initiation factor 1A (EIF-1A) (EIF-4C) | 81789 | Unknown |
| Eukaryotic translation initiation factor 3 subunit G (eIF3g) | 80484 | Unknown |
| FK506-binding protein 1B (FKBP) (Peptidyl-prolyl cis-trans isomerase) (PPIase) (Rapamycin-binding protein) | 102097 | Unknown |
| fructose bisphosphate aldolase | 104720 | Unknown |
| Galactoside O-acetyltransferase | 66294 | Unknown |
| Glutathione peroxidase | 96955 | Unknown |
| Glycine cleavage system H protein-like protein | 77821 | Unknown |
| Histone H2A | 104400 | Unknown |
| Histone H2B | 104409 | Unknown |
| Indoleamine 2,3-dioxygenase family protein | 93945 | Unknown |
| Methyltransferase | 42151 | Unknown |
| Myosin regulatory light chain cdc4 | 101854 | Unknown |
| NAD(P)H-dependent D-xylose reductase (XR) | 103071 | Unknown |
| NADPH-dependent D-xylose reductase II,III (XR) | 55348 | Unknown |
| Nascent polypeptide-associated complex subunit alpha (NAC-alpha) (Alpha-NAC) | 81890 | Unknown |
| Non-histone chromosomal protein 6 | 55753 | Unknown |
| Nuclear transport factor 2 (NTF-2) | 57583 | Unknown |
| Perilipin MPL1-like protein | 79008 | Unknown |
| Peroxiredoxin (AsPrx) (Thioredoxin peroxidase) | 68536 | Unknown |
| Probable cytochrome b5 | 99827 | Unknown |
| Profilin | 102150 | Unknown |
| Putative oxidoreductase | 104397 | Unknown |
| Ribosomal protein L28e | 86511 | Unknown |
| Saccharopine dehydrogenase [NAD+, L-lysine-forming] (Lysine--2-oxoglutarate reductase) (SDH) | 93017 | Unknown |
| Secreted aspartic proteinase | 73874 | Unknown |
| Short chain dehydrogenase/reductase family oxidoreductase | 39428 | Unknown |
| Small nuclear ribonucleoprotein F | 54955 | Unknown |
| thioredoxin | 39961 | Unknown |
| Translationally-controlled tumor protein homolog (TCTP) | 77315 | Unknown |
| Ubiquitin-40S ribosomal protein | 103787 | Unknown |
| Ubiquitin-conjugating enzyme E2 variant 1 | 104299 | Unknown |
| Ubiquitin-like protein | 80978 | Unknown |
| uncharacterized protein | 36895 | Unknown |
| uncharacterized protein | 61750 | Unknown |
| uncharacterized protein | 66481 | Unknown |
| uncharacterized protein | 70589 | Unknown |
| uncharacterized protein | 70844 | Unknown |
| uncharacterized protein | 72967 | Unknown |
| uncharacterized protein | 76370 | Unknown |
| uncharacterized protein | 80289 | Unknown |
| uncharacterized protein | 84988 | Unknown |
| uncharacterized protein | 85077 | Unknown |
| uncharacterized protein | 95236 | Unknown |
| uncharacterized protein | 100587 | Unknown |
| uncharacterized protein | 101182 | Unknown |
| uncharacterized protein | 101783 | Unknown |
| uncharacterized protein | 101944 | Unknown |
| uncharacterized protein | 102090 | Unknown |
| uncharacterized protein | 102285 | Unknown |
| uncharacterized protein | 102698 | Unknown |
| uncharacterized protein | 103805 | Unknown |
| uncharacterized protein | 104580 | Unknown |
| uncharacterized protein | 105888 | Unknown |
| uncharacterized protein | 106261 | Unknown |
| uncharacterized protein | 108592 | Unknown |
| Uricase | 87102 | Unknown |

Supplemental Table 7 Digital quantification of mRNA level of genes coding for differentially expressed secreted proteins by RNA-seqa

| Gene ID^b^ | Locus | EP713 (FPKM) | EP155 (FPKM) | Fold change |
| --- | --- | --- | --- | --- |
| 107223 | scaffold_5:1922991-1925273 | 54.31 | 4.79 | 11.34 |
| 105629 | scaffold_1:6173252-6174809 | 52.32 | 5.21 | 10.04 |
| 36288 | scaffold_1:6381615-6383071 | 87.27 | 11.03 | 7.91 |
| 39428 | scaffold_3:1818738-1820002 | 296.82 | 47.64 | 6.23 |
| 83754 | scaffold_9:2146986-2149104 | 10.16 | 2.12 | 4.80 |
| 105554 | scaffold_1:5631019-5632608 | 31.78 | 7.49 | 4.24 |
| 79318 | scaffold_3:562276-564355 | 240.27 | 64.92 | 3.70 |
| 58903 | scaffold_1:812440-814584 | 60.20 | 20.49 | 2.94 |
| 61750 | scaffold_3:4783517-4784588 | 11.38 | 4.05 | 2.81 |
| 74162 | scaffold_6:2156174-2157690 | 3.32 | 1.26 | 2.64 |
| 82357 | scaffold_2:4461500-4462635 | 191.81 | 73.42 | 2.61 |
| 73874 | scaffold_6:895909-899520 | 70.90 | 28.34 | 2.50 |
| 93693 | scaffold_9:1592717-1593882 | 79.48 | 33.23 | 2.39 |
| 70319 | scaffold_5:4113535-4114667 | 33.24 | 13.92 | 2.39 |
| 106275 | scaffold_4:4734266-4735889 | 327.37 | 149.64 | 2.19 |
| 97118 | scaffold_1:6834881-6837021 | 178.87 | 90.79 | 1.97 |
| 79283 | scaffold_4:4940264-4942942 | 601.15 | 318.69 | 1.89 |
| 76520 | scaffold_1:1108859-1110594 | 84.49 | 46.63 | 1.81 |
| 102966 | scaffold_2:4128359-4130406 | 414.58 | 230.27 | 1.80 |
| 64525 | scaffold_6:2220822-2222107 | 82.69 | 47.98 | 1.72 |
| 54024 | scaffold_1:4985965-4986373 | 954.86 | 559.42 | 1.71 |
| 105888 | scaffold_4:1265535-1267073 | 103.08 | 61.49 | 1.68 |
| 103417 | scaffold_9:702454-703381 | 835.96 | 504.82 | 1.66 |
| 101628 | scaffold_4:3839998-3841715 | 280.84 | 170.07 | 1.65 |
| 41127 | scaffold_3:3804784-3806367 | 15.69 | 9.57 | 1.64 |
| 104299 | scaffold_8:1977402-1978811 | 89.10 | 54.71 | 1.63 |
| 102227 | scaffold_3:4966855-4968443 | 750.87 | 461.35 | 1.63 |
| 81890 | scaffold_2:2603001-2604386 | 311.85 | 193.97 | 1.61 |
| 108972 | scaffold_8:2900807-2903875 | 229.96 | 144.51 | 1.59 |
| 104397 | scaffold_8:3125813-3127126 | 30.60 | 19.32 | 1.58 |
| 103140 | scaffold_7:1249525-1251025 | 508.46 | 322.84 | 1.57 |
| 103579 | scaffold_9:2067152-2069399 | 427.15 | 271.32 | 1.57 |
| 102329 | scaffold_5:3809800-3811227 | 277.99 | 176.62 | 1.57 |
| 90732 | scaffold_5:4229811-4231244 | 451.48 | 287.61 | 1.57 |
| 93992 | scaffold_9:2627999-2629670 | 81.51 | 52.72 | 1.55 |
| 40411 | scaffold_3:1589897-1592282 | 117.68 | 77.58 | 1.52 |
| 67753 | scaffold_4:187536-188469 | 5.98 | 4.02 | 1.49 |
| 100785 | scaffold_1:3032801-3033978 | 465.27 | 317.71 | 1.46 |
| 48583 | scaffold_6:194414-196628 | 97.46 | 68.01 | 1.43 |
| 86511 | scaffold_1:915313-916658 | 223.11 | 160.66 | 1.39 |
| 71516 | scaffold_2:2084950-2086784 | 25.06 | 19.05 | 1.32 |
| 107093 | scaffold_9:2682129-2684351 | 128.05 | 97.62 | 1.31 |
| 75598 | scaffold_10:480624-482535 | 291.90 | 224.15 | 1.30 |
| 54955 | scaffold_4:4838468-4839115 | 100.96 | 78.34 | 1.29 |
| 99052 | scaffold_7:1360530-1362781 | 125.34 | 100.51 | 1.25 |
| 74957 | scaffold_8:3196257-3197348 | 3.31 | 2.66 | 1.24 |
| 87808 | scaffold_1:5895319-5897746 | 166.18 | 136.06 | 1.22 |
| 39110 | scaffold_3:1034143-1035975 | 79.24 | 65.35 | 1.21 |
| 91352 | scaffold_5:2093526-2094661 | 222.44 | 184.15 | 1.21 |
| 55348 | scaffold_3:3652632-3654495 | 119.86 | 101.66 | 1.18 |
| 102150 | scaffold_3:4240011-4240795 | 344.34 | 292.59 | 1.18 |
| 97441 | scaffold_4:3001756-3003662 | 118.06 | 106.10 | 1.11 |
| 81491 | scaffold_5:1081585-1085760 | 20.08 | 18.19 | 1.10 |
| 58361 | scaffold_10:609614-611366 | 79.04 | 72.16 | 1.10 |
| 93073 | scaffold_7:2197342-2205057 | 50.24 | 48.20 | 1.04 |
| 55987 | scaffold_5:753043-755823 | 1826.34 | 1778.15 | 1.03 |
| 90536 | scaffold_3:4545318-4549157 | 396.55 | 407.81 | 0.97 |
| 102698 | scaffold_2:1171278-1171584 | 39.52 | 42.67 | 0.93 |
| 70844 | scaffold_5:2096351-2097773 | 272.33 | 300.70 | 0.91 |
| 84745 | scaffold_8:1502105-1504929 | 332.29 | 377.58 | 0.88 |
| 88657 | scaffold_4:2186791-2187787 | 157.06 | 183.63 | 0.86 |
| 101684 | scaffold_4:4321141-4322996 | 1368.62 | 1617.04 | 0.85 |
| 65827 | scaffold_3:307428-309090 | 24.20 | 28.86 | 0.84 |
| 86663 | scaffold_1:1487432-1489974 | 4.04 | 4.86 | 0.83 |
| 82931 | scaffold_7:1985056-1988233 | 346.40 | 417.32 | 0.83 |
| 108053 | scaffold_7:2725906-2727396 | 6.79 | 8.54 | 0.80 |
| 95432 | scaffold_6:3388358-3390331 | 0.73 | 0.92 | 0.79 |
| 89402 | scaffold_3:579152-586581 | 159.42 | 215.73 | 0.74 |
| 87102 | scaffold_1:3118589-3120266 | 58.64 | 79.64 | 0.74 |
| 97515 | scaffold_4:3789168-3791080 | 17.54 | 24.69 | 0.71 |
| 103787 | scaffold_8:305084-306351 | 2653.33 | 3858.53 | 0.69 |
| 99792 | scaffold_8:981295-983635 | 248.40 | 374.90 | 0.66 |
| 67775 | scaffold_4:261313-263296 | 408.92 | 715.83 | 0.57 |
| 99412 | scaffold_9:1692912-1695191 | 24.04 | 43.25 | 0.56 |
| 90079 | scaffold_3:3099851-3101115 | 45.86 | 83.57 | 0.55 |
| 103771 | scaffold_6:1172209-1173597 | 280.70 | 528.25 | 0.53 |
| 80754 | scaffold_5:3929286-3930815 | 559.96 | 1060.34 | 0.53 |
| 74613 | scaffold_8:1866322-1867221 | 12.00 | 22.94 | 0.52 |
| 96317 | scaffold_3:200515-201570 | 13.39 | 26.55 | 0.50 |
| 104348 | scaffold_8:2641085-2644904 | 576.90 | 1188.60 | 0.49 |
| 93945 | scaffold_9:2481064-2483311 | 30.17 | 63.32 | 0.48 |
| 104720 | scaffold_2:5036843-5038606 | 274.50 | 591.46 | 0.46 |
| 95002 | scaffold_8:2108648-2110210 | 30.10 | 64.95 | 0.46 |
| 95286 | scaffold_6:2922819-2928351 | 66.66 | 166.46 | 0.40 |
| 103448 | scaffold_9:871162-872377 | 232.09 | 612.28 | 0.38 |
| 69709 | scaffold_1:266108-267821 | 95.05 | 260.43 | 0.36 |
| 84988 | scaffold_8:2606969-2607947 | 8.66 | 23.98 | 0.36 |
| 85870 | scaffold_2:5307040-5310670 | 549.59 | 1577.00 | 0.35 |
| 103361 | scaffold_9:285343-286859 | 46.00 | 132.18 | 0.35 |
| 108828 | scaffold_8:1562946-1565093 | 295.86 | 872.59 | 0.34 |
| 107257 | scaffold_5:1573699-1574951 | 64.05 | 198.29 | 0.32 |
| 82952 | scaffold_7:2057242-2062242 | 87.71 | 327.92 | 0.27 |
| 42588 | scaffold_5:3218150-3221257 | 220.53 | 848.11 | 0.26 |
| 40356 | scaffold_3:2172880-2177294 | 31.03 | 142.23 | 0.22 |
| 93265 | scaffold_7:3243257-3245552 | 151.41 | 718.68 | 0.21 |
| 107379 | scaffold_5:388901-390920 | 35.98 | 178.89 | 0.20 |
| 107212 | scaffold_4:3985473-3987496 | 9.48 | 87.01 | 0.11 |
| 43693 | scaffold_5:1017987-1021346 | 28.31 | 295.68 | 0.10 |

^a^Sample was from one of the three replicates of iTRAQ experiments

^b^Accession number from *Cryphonectria parasitica* database v1.0.

Supplemental Table 8 List of differentially secreted proteins identified with single peptide

| File, Scan (s) | Sequence | MH+ | Difference (MH+) | Charge | Rank | XC | DeltaCn | Sp | RSp | Ions | Cover  % | Refenrence^a^ |
| --- | --- | --- | --- | --- | --- | --- | --- | --- | --- | --- | --- | --- |
| P11326M3_1,6798 | R.PFLQDLVNK.E | 1362.459 | -0.079 | 2 | 1 | 2.5049 | 0.3018 | 1030.7 | 3 | 13|16 | 9.89 | jgi|Crypa1|54955|fgenesh1_kg.C_scaffold_2000597  aa: 90  MW: 10327.75,  PI: 5.3  Functional annotation: small nuclear ribonucleoprotein |
| P11326M3_1,6810 | R.PFLQDLVNK.E | 1362.459 | -0.03 | 2 | 1 | 2.4216 | 0.2246 | 855.9 | 1 | 13|16 |
| P11326M3_1,6856 | R.PFLQDLVNK.E | 1362.459 | 1.058 | 2 | 1 | 2.1137 | 0.2019 | 867.5 | 1 | 12|16 |
| P11326M3_2,6912 | R.PFLQDLVNK.E | 1362.459 | 0.561 | 2 | 1 | 2.1881 | 0.2125 | 1039.3 | 1 | 13|16 |
| P11326M3_2,6919 | R.PFLQDLVNK.E | 1362.459 | 0.066 | 2 | 1 | 3.0055 | 0.3455 | 1610 | 1 | 15|16 |
| P11326M3_3,6935 | R.PFLQDLVNK.E | 1362.459 | -0.146 | 2 | 1 | 2.252 | 0.3329 | 978.9 | 2 | 12|16 |
| P11326M1_1,5679 | R.FNDPADFGIR.I | 1296.3431 | -0.1309 | 2 | 1 | 2.1658 | 0.4008 | 377.4 | 1 | 13|18 | 3.07 | jgi|Crypa1|55348|fgenesh1_kg.C_scaffold_3000361  aa: 325  MW: 36430.6,  PI: 6.15  Functional annotation: NADPH-dependent D-xylose reductase |
| P11326M1_1,5687 | R.FNDPADFGIR.I | 1296.3431 | -0.0879 | 2 | 1 | 2.3995 | 0.3586 | 601.1 | 1 | 16|18 |
| P11326M1_2,5801 | R.FNDPADFGIR.I | 1296.3431 | 0.3981 | 2 | 1 | 2.6586 | 0.4085 | 941.1 | 1 | 16|18 |
| P11326M1_2,5808 | R.FNDPADFGIR.I | 1296.3431 | 0.6121 | 2 | 1 | 2.2867 | 0.4059 | 732.8 | 1 | 15|18 |
| P11326M1_2,6101 | R.FNDPADFGIR.I | 1296.3431 | -0.0629 | 2 | 1 | 1.8502 | 0.1538 | 660.3 | 1 | 13|18 |
| P11326M1_3,5783 | R.FNDPADFGIR.I | 1296.3431 | 0.1591 | 2 | 1 | 1.8718 | 0.2874 | 497.7 | 1 | 13|18 |
| P11326M1_3,5811 | R.FNDPADFGIR.I | 1296.3431 | 0.0531 | 2 | 1 | 2.5229 | 0.3468 | 676.2 | 1 | 16|18 |
| P11326M1_1,5498 | K.QLSQGADFFDER.Q | 1557.5761 | 0.9291 | 2 | 1 | 2.8718 | 0.4425 | 1569.3 | 1 | 19|22 | 14.46 | jgi|Crypa1|102698|estExt_fgenesh1_kg.C_50011  aa: 82  MW: 9204.46,  PI: 6.17  Functional annotation: uncharacterized |
| P11326M1_1,5503 | K.QLSQGADFFDER.Q | 1557.5761 | 0.1861 | 2 | 1 | 2.8718 | 0.452 | 1276.3 | 1 | 18|22 |
| P11326M1_2,5593 | K.QLSQGADFFDER.Q | 1557.5761 | 0.1991 | 2 | 1 | 3.4769 | 0.51 | 1242.4 | 1 | 19|22 |
| P11326M1_2,5605 | K.QLSQGADFFDER.Q | 1557.5761 | -0.8949 | 2 | 1 | 3.9631 | 0.4518 | 2235.2 | 1 | 22|22 |
| P11326M1_2,5607 | K.QLSQGADFFDER.Q | 1557.5761 | 0.9291 | 2 | 1 | 3.2341 | 0.3852 | 1372.3 | 1 | 18|22 |
| P11326M1_2,5612 | K.QLSQGADFFDER.Q | 1557.5761 | 0.1591 | 2 | 1 | 3.2572 | 0.4945 | 1414.4 | 1 | 19|22 |
| P11326M1_3,5579 | K.QLSQGADFFDER.Q | 1557.5761 | 0.0231 | 2 | 1 | 3.0258 | 0.4493 | 1339.6 | 1 | 19|22 |
| P11326M1_3,5586 | K.QLSQGADFFDER.Q | 1557.5761 | -0.6159 | 2 | 1 | 3.6406 | 0.5125 | 1719.1 | 1 | 21|22 |
| P11326M2_1,6351 | K.TDLEFFK.I | 1188.2142 | -1.1798 | 2 | 1 | 2.6325 | 0.2833 | 1096.7 | 1 | 12|12 | 1.94 | jgi|Crypa1|95002|estExt_Genewise1Plus.C_90821  aa:359  MW: 36058.34,  PI: 4.34  Functional annotation: Endoglucanase |
| P11326M2_2,6300 | K.TDLEFFK.I | 1188.2142 | 2.1762 | 2 | 1 | 2.2843 | 0.281 | 943.5 | 1 | 11|12 |
| P11326M2_2,6303 | K.TDLEFFK.I | 1188.2142 | 1.9162 | 2 | 1 | 2.5871 | 0.3252 | 1306.5 | 1 | 12|12 |
| P11326M2_3,6227 | K.TDLEFFK.I | 1188.2142 | -1.3578 | 2 | 1 | 2.853 | 0.2886 | 1287.1 | 1 | 12|12 |
| P11326M2_3,6255 | K.TDLEFFK.I | 1188.2142 | -2.2568 | 2 | 1 | 2.723 | 0.2634 | 1443.6 | 1 | 12|12 |
| P11326M3_1,6200 | K.TDLEFFK.I | 1188.2142 | 0.8972 | 2 | 1 | 1.9363 | 0.2778 | 1085.4 | 1 | 12|12 |
| P11326M3_1,1692 | K.ASSGSGGLSK.G | 1139.1016 | 0.7266 | 2 | 1 | 2.4459 | 0.4055 | 820.8 | 1 | 16|18 | 2.59 | jgi|Crypa1|69709|fgenesh1_pg.C_scaffold_3000775  aa: 385  MW: 39932.8,  PI: 4.88  Functional  annotation: ATP-dependent DNA helicase |
| P11326M3_2,1663 | K.ASSGSGGLSK.G | 1139.1016 | 0.3616 | 2 | 1 | 2.6611 | 0.3801 | 1124.9 | 1 | 14|18 |
| P11326M3_2,1673 | K.ASSGSGGLSK.G | 1139.1016 | 0.2376 | 2 | 1 | 2.9076 | 0.454 | 1050.6 | 1 | 16|18 |
| P11326M3_3,1670 | K.ASSGSGGLSK.G | 1139.1016 | 0.1786 | 2 | 1 | 2.7719 | 0.4142 | 1189.9 | 1 | 17|18 |
| P11326M3_3,1682 | K.ASSGSGGLSK.G | 1139.1016 | -1.1934 | 2 | 1 | 2.991 | 0.4411 | 851.7 | 1 | 14|18 |
| P11326M2_1,5937 | K.SYDYLQSLNK.F | 1519.5406 | -1.0664 | 2 | 1 | 2.5351 | 0.3462 | 946.2 | 1 | 16|18 | 2.08 | jgi|Crypa1|79318|estExt_Genewise1.C_30066  aa: 480  MW: 52879.6,  PI: 4.82  Functional annotation: 3-phytase A |
| P11326M2_1,5945 | K.SYDYLQSLNK.F | 1519.5406 | 0.2646 | 2 | 1 | 2.7684 | 0.3771 | 1379.6 | 1 | 16|18 |
| P11326M2_1,5953 | K.SYDYLQSLNK.F | 1519.5406 | 0.0826 | 2 | 1 | 2.9472 | 0.4201 | 1448.6 | 1 | 17|18 |
| P11326M2_2,5876 | K.SYDYLQSLNK.F | 1519.5406 | 0.1676 | 2 | 1 | 2.9431 | 0.407 | 1081 | 1 | 15|18 |
| P11326M2_2,5883 | K.SYDYLQSLNK.F | 1519.5406 | 0.2416 | 2 | 1 | 2.9893 | 0.4384 | 1393 | 1 | 16|18 |
| P11326M2_3,5829 | K.SYDYLQSLNK.F | 1519.5406 | 0.2256 | 2 | 1 | 2.9015 | 0.4545 | 1796.8 | 1 | 18|18 |
| P11326M2_3,5836 | K.SYDYLQSLNK.F | 1519.5406 | 0.3116 | 2 | 1 | 2.7969 | 0.4319 | 1130.2 | 1 | 15|18 |
| P11326M2_1,3786 | K.NILAEEEK.L | 1234.2413 | -0.0187 | 2 | 1 | 2.6732 | 0.1639 | 1199.3 | 1 | 14|14 | 2.75 | jgi|Crypa1|39428|e_gw1.3.894.1  aa: 290  MW: 31682.42,  PI: 6.53  Functional annotation: oxidoreductase |
| P11326M2_1,3793 | K.NILAEEEK.L | 1234.2413 | 0.0013 | 2 | 1 | 2.5318 | 0.1829 | 1147.8 | 1 | 14|14 |
| P11326M2_1,3825 | K.NILAEEEK.L | 1234.2413 | 2.0113 | 2 | 1 | 2.8005 | 0.2528 | 1256.9 | 1 | 14|14 |
| P11326M2_1,3834 | K.NILAEEEK.L | 1234.2413 | 2.1243 | 2 | 1 | 2.5242 | 0.1899 | 1341.4 | 1 | 14|14 |
| P11326M2_2,3667 | K.NILAEEEK.L | 1234.2413 | 0.2073 | 2 | 1 | 2.2386 | 0.1721 | 1483.9 | 1 | 14|14 |
| P11326M2_2,3676 | K.NILAEEEK.L | 1234.2413 | 0.5573 | 2 | 1 | 2.7139 | 0.2352 | 1535.9 | 1 | 14|14 |
| P11326M2_2,3713 | K.NILAEEEK.L | 1234.2413 | 1.8193 | 2 | 1 | 2.6291 | 0.2485 | 1473.5 | 1 | 14|14 |
| P11326M2_3,3696 | K.NILAEEEK.L | 1234.2413 | -0.1427 | 2 | 1 | 2.3822 | 0.1967 | 1126.4 | 1 | 14|14 |
| P11326M2_3,3702 | K.NILAEEEK.L | 1234.2413 | 0.3823 | 2 | 1 | 2.4796 | 0.2058 | 1069 | 1 | 14|14 |
| P11326M2_3,3721 | K.NILAEEEK.L | 1234.2413 | 1.5123 | 2 | 1 | 2.2688 | 0.2246 | 1546.6 | 1 | 14|14 |
| P11326M2_3,3732 | K.NILAEEEK.L | 1234.2413 | 1.6103 | 2 | 1 | 2.5461 | 0.3119 | 1632.1 | 1 | 14|14 |
